# Checklist of the freshwater fishes of Colombia: a Darwin Core alternative to the updating problem

**DOI:** 10.3897/zookeys.708.13897

**Published:** 2017-10-13

**Authors:** Carlos DoNascimiento, Edgar Esteban Herrera-Collazos, Guido A. Herrera-R., Armando Ortega-Lara, Francisco A. Villa-Navarro, José Saulo Usma Oviedo, Javier A. Maldonado-Ocampo

**Affiliations:** 1 Instituto de Investigación de Recursos Biológicos Alexander von Humboldt, Villa de Leyva, Colombia; 2 Laboratorio de Ictiología, Unidad de Ecología y Sistemática (UNESIS), Departamento de Biología, Facultad de Ciencias, Pontificia Universidad Javeriana, Bogotá, DC, Colombia; 3 Grupo de Investigación en Peces Neotropicales, Fundación para la Investigación y el Desarrollo Sostenible (FUNINDES), Cali, Colombia; 4 Grupo de Investigación en Zoología, Facultad de Ciencias, Universidad del Tolima, Ibagué, Colombia; 5 Programa Ecosistemas de Agua Dulce de WWF Colombia, Cali, Colombia

**Keywords:** Aquatic ecosystems, conservation, endemic, richness, South America

## Abstract

The present work is part of a process to create a Catalogue of the Freshwater Fishes of Colombia and consisted in the depuration and updating of the taxonomic and geographic components of the checklist of the freshwater fishes of Colombia. An exhaustive revision of the 1435 species recorded in 2008 was necessary to: 1. Add new species described since 2009 and species originally described from Colombia but inadvertently omitted in 2008; 2. Add new records of already described species; 3. Delete species whose presence in Colombia was not supported by voucher specimens in ichthyological collections; and 4. Revise the geographic distribution of the species listed in 2008. This process resulted in the following numbers: 1. Total number of freshwater fish species in Colombia: 1494; 2. Number of species recorded by hydrographic region - Amazon: 706, Orinoco: 663, Caribbean: 223, Magdalena-Cauca: 220, Pacific: 130; and 3. Number of endemic species: 374 (76% from the trans-Andean region). Updating the current checklist is a fundamental requirement to ensure its incorporation in the decision-making process with regard to the conservation of Colombian aquatic species and ecosystems, which are facing transformation processes as a result of activities such as mining, construction of hydroelectric plants, expansion of the agricultural frontier and subsequent deforestation, industrial and domestic pollution, development of waterways, introduction of exotic species, and climate change.

## Abstract (es)

El presente trabajo es parte de un proceso para crear un Catálogo de Peces de Agua Dulce de Colombia y consistió en la depuración y actualización de los componentes taxonómico y geográfico del listado de peces de agua dulce de Colombia. Una revisión exhaustiva de las 1435 especies registradas en 2008 fue necesaria para: 1. Adicionar las especies nuevas descritas desde 2009 y especies descritas originalmente para Colombia pero omitidas inadvertidamente en 2008; 2. Adicionar nuevos registros de especies ya descritas; 3. Eliminar especies cuya presencia en Colombia no estaba soportada por especímenes en colecciones ictiológicas; y 4. Revisar la distribución geográfica de las especies listadas en 2008. Este proceso resultó en las siguientes cifras: 1. Número total de especies de peces de agua dulce en Colombia: 1494; 2. Número de especies registradas por región hidrográfica - Amazonas: 706, Orinoco: 663, Caribe: 223, Magdalena-Cauca: 220, Pacífico: 130; y 3. Número de especies endémicas 374 (76% distribuidas en la región transandina). La actualización del presente listado es un requerimiento fundamental para asegurar su incorporación en el proceso de toma de decisiones en lo concerniente a la conservación de las especies y ecosistemas acuáticos colombianos, los cuales están enfrentando procesos de transformación, como resultado de actividades como la minería, construcción de plantas hidroeléctricas, expansión de la frontera agropecuaria y subsecuente desforestación, polución industrial y doméstica, desarrollo de hidrovías, introducción de especies exóticas y cambio climático.

*This paper is in honour of the late Richard Peter Vari, who was a co-author of the checklist of the freshwater fishes of Colombia in 2008, a paper that highlighted the importance of the Colombian ichthyofauna in the Neotropical and global contexts, and addressed important issues in terms of the conservation challenges and needs to improve the sampling effort of the continental aquatic systems in Colombia. After almost a decade since that checklist was published, the challenges and tasks pointed out by Richard and co-authors have been attained in several ways in the present paper. He will always be remembered not just for his enormous contribution to the Colombian and Neotropical ichthyology, but most especially for his kindness and willing collaboration*.

## Introduction

Since Maldonado-Ocampo et al. (2008), important advances have been made to update the current number of fish species found in Colombian freshwater ecosystems. The first is new fieldwork, which has led to significant range expansions in known distribution of species and new species discoveries; the second is new revisions of existing ichthyological collections. Several checklists have been published at local or regional scales (e.g. Lasso et al. 2009, Villa-Navarro et al. 2011, Ortega-Lara et al. 2012); and an important amount of information has also been published in books that include aspects on ecology, fisheries, threats, and migratory status (e.g. Usma et al. 2009, 2013, Lasso et al. 2011a, Maldonado-Ocampo et al. 2012, Mojica et al. 2012, Zapata and Usma 2013).

During this time frame (nine years), fieldwork efforts have been conducted mainly in three of the five hydrographic Colombian regions as delimited by IDEAM (2015): Amazon (Putumayo, Caquetá, Guainía, and Vaupés rivers), Orinoco (piedmont rivers of the Meta and Guaviare basins, and rivers draining directly to the Orinoco as the Bita and Inírida), and Magdalena-Cauca (especially the upper and middle sectors). Some collections in other important rivers as the Catatumbo, Sinú, and Atrato (Caribbean system), San Juan and Dagua (Pacific system), have also been conducted. All fishes collected in these rivers are deposited in the following ichthyological collections of Colombia (acronyms follow Sabaj, 2016): CAR, CIACOL, CIUA, CP-UCO, CZUT-IC, IAvH-P, ICN-MHN, IMCN, and MPUJ.

Complementing these fieldwork and taxonomic efforts that improve the information quality of the current annotated checklist, an important project “Catalogue of the Freshwater Fishes of Colombia” is in progress. From mid-2014, all previously mentioned ichthyological collections began a data depuration process. First, and the most important task, all the catalogue data bases associated with these collections are being translated to the Darwin Core standard, allowing the data to be adequately formatted to make them freely accessible through facilitators such as GBIF and SiB Colombia (Colombia Biodiversity Information System: www.sibcolombia.net).

At the moment, all available records to mid-2017 in CAR, CIACOL, CICH, CIUA, CP-UCO, CZUT-IC, IAvH-P, ICN-MHN, IMCN, and MPUJ from the hydrographic regions of Orinoco (29000), Magdalena-Cauca (22000), Amazon (14000), Caribbean (5000), and Pacific (1000), have been updated in the following data fields: Occurrence, Organism, Event, Location, Identificacion, and Taxon. The project hopes to conclude this data depuration process for all available records in these collections (around 75000 in total) by December of 2017. The updated annotated checklist presented here and the data available in the collections is the basis for the “Catalogue of the Freshwater Fishes of Colombia” that will be hosted at the Catalogue of Species of the Colombia Biodiversity Information System (http://www.biodiversidad.co/#/), and linked to the website of the Colombian Ichthyological Association (ACICTIOS, http://acictios.org).

For the first time in Colombia, freshwater ecosystems are the focus of public attention and different strategies are being discussed by the academy and decision-makers to develop adequate conservation and sustainable use strategies for these ecosystems and their biota (Vilardi et al. 2014, Tognelli et al. 2016). The updated information presented here plus the continuous work in the collections to update and edit the data bases will be of extreme importance in terms of the quality and quantity of available information to facilitate evidence-based decision making that can help counter the numerous threats facing aquatic ecosystems (hydropower development, mining, pollution, deforestation, exotic species invasion, and climate change scenarios) that are affecting this enormous fish diversity.

## Methods

The starting point for the updating process and corrections to the checklist of the freshwater fishes of Colombia was the checklist of Maldonado-Ocampo et al. (2008). This revision incorporated four main aspects: 1. Addition of newly described species since 2009 and species originally described for Colombia but inadvertently omitted in Maldonado-Ocampo et al. (2008), 2. Addition of new records of already described species, 3. Deletion of nominal species whose presence in Colombia was not supported by voucher specimens in collections, and 4. Revision of the geographic distribution of those species previously listed in Maldonado-Ocampo et al. (2008). The main sources used for this revision consisted in research articles describing new species, taxonomic and systematic revisions of genera and families, direct examination of specimens catalogued at CZUT-IC, IAvH-P, IMCN, and MPUJ, and to a lesser extent, regional checklists explicitly supported by catalogued lots of specimens for each species entry, and lists of examined material of taxonomic and systematic works dealing with Neotropical groups.

The complete list of species was assembled using the Darwin Core standard (DwC) and is available both as Suppl. material 1 and through the Integrated Publishing Tool of the Colombian node of GBIF, the SiB Colombia (http://www.sibcolombia.net/), as version 2.5 at http://doi.org/10.15472/numrso. Future updates of the list will be published in the latter repository. Changes incorporated to each new version of the list will be summarized in the respective metadata section of the electronic resource. A curatorial team presented during the celebration of the biannual meeting of the “*Asociación Colombiana de Ictiólogos*” (ACICTIOS) will be in charge of the updating and revision process of the list and of its periodic publication (annually or semestrally, depending on the amount of changes accumulated every six months). Below we list fields for which we consider a further detailed explanation of the criteria adopted is needed in order to provide an unequivocal framework for the definitions used for each field.

**taxonID**. This field is defined as a unique and stable-through-time alphanumeric identifier (taxonomic identifier) provided by Life Science Identifier (LSID).

**namePublishedIn**. This field links each species name to its respective source (i.e., a bibliographic reference) and was restricted to include only those species originally described from Colombia, i.e., only the bibliographic reference of the original description of species that listed Colombian specimens (in some cases from border regions as well) as types, as well as descriptions that explicitly listed Colombian specimens as non-types.

**references**. This field is reserved for those species not described from Colombia, but whose presence in the country is supported by a bibliographic reference. In this case, we made a special effort to locate the first chronological reference that explicitly mentioned Colombian specimens collected and deposited in national or international collections or that provided images that allows unambiguous recognition of a determined species, for each main hydrographic system. In some cases, we complemented these primary bibliographic references with more recent references, where additional catalogued lots are provided. Therefore, this approach is somewhat different from the practice implemented in Maldonado-Ocampo et al. (2008), in order to avoid the inclusion of references that are merely lists solely based on previous published works. Future publications reporting new species or distribution extensions of those species already included in this checklist are encouraged to indicate cataloged lots that support them. This action allows traceability and verification of records whenever needed, and facilitates the implementation of new taxonomical arrangements when they need to be proposed.

**associatedOccurrences**. As in the previous field, here we supported the presence of species not originally described from Colombia. We listed catalogued lots (preferably available in Colombian collections) for species not supported by bibliographic references or to complement references that do not provide lots. Lots are provided for each main hydrographic system. In addition, we also listed here catalogued lots corresponding to types.

**locationRemarks**. Only includes species that are endemic to the Colombian territory.

**dynamicProperties**. Corresponds to the column CT (endangered and threatened species) used in Maldonado-Ocampo et al. (2008). The information in this field was based in Mojica et al. (2012) and since the scope of the present list is of national scale, threat categories are restricted to Colombia.

**occurrenceRemarks**. Indicates the type of commercial use of each species (i.e., Fisheries, Ornamental). The list of fish species included in Colombian inland fisheries is from Lasso et al. (2011a), while the list of ornamental species is from Ortega-Lara et al. (2015) and Ortega-Lara (2016).

**locality**. Type localities of species described from Colombia are listed as they are cited in their respective original descriptions or as they appear in the Catalogue of Fishes (http://researcharchive.calacademy.org/research/ichthyology/catalog/fishcatmain.asp).

Institutional acronyms follow Sabaj (2016). Species formerly indicated as indeterminate (sp) in Maldonado-Ocampo et al. (2008) are listed at the genus level, since they represent still undescribed species and account for the presence of genera not represented by already described species in Colombia. Classification of taxa of higher taxonomic ranks (Class to Suborder) and arrangement order of respective taxa followed the proposal of Betancur-R. et al. (2017). The following proposals of classification were adopted for the indicated groups: Tagliacollo et al. (2016): Gymnotiformes; Mirande (2009, 2010) and Oliveira et al. (2011): Characiformes, but in incongruent cases, we just adopted the latest published proposal (i.e., Oliveira et al. 2011), not implying in any form a validation over the former works of Mirande; Thomaz et al. (2015): Stevardiinae; Sullivan et al. (2006): Siluriformes; Lujan et al. (2015a): Hypostominae; Roxo et al. (2014): Hypoptopomatinae and Otothyrinae; Covain et al. (2016): Loricariinae; Birindelli (2014): Doradoidea; Sullivan et al. (2013a): Pimelodoidea; López-Fernández et al. (2010): Cichlidae. For remaining groups we followed Nelson et al. (2016).

## Results

### New species described from Colombia

Since the publication of the most recent checklist (Maldonado-Ocampo et al. 2008), 106 new species of freshwater fishes have been described from Colombia (Suppl. material 2), at a rate of approximately ten species per year. This places the total number of species described or recorded for Colombia at 1494 freshwater fishes (Table 1), a slightly higher figure than the last recorded number of 1435 species (Maldonado-Ocampo et al. 2008). This slight increment in the total number is especially noteworthy since our careful review of each species name in Maldonado-Ocampo et al. (2008) led to the deletion of 202 nominal species that lacked solid support for keeping them in the current version of the checklist (see below). The assessment process of some doubtful species led to the inclusion of most of the newly recorded species presented here (see also below), through the direct examination of lots of specimens that were formerly used to support those doubtful species. These figures together, still place Colombia as the second most-species rich country in the world in terms of its freshwater ichthyofauna. The number of species by hydrographic system is presented in the Table 2, where the Amazon accounts for the highest number of species. A remarkable total of 374 endemic species is now reported from Colombia; 76% of them are species from trans-Andean basins (286 species), and stand as testimony of the unique geological and climatic events that shaped the biogeographic history and taxonomic diversification of the Colombian ichthyological biota.

**Table 1.** Number and percentage of species of freshwater fishes by order in Maldonado-Ocampo et al. (2008) compared to the present checklist.

Order	Number of species in Maldonado-Ocampo et al. (2008)	%	Number of species in the present work	%
Characiformes	637	44.4	644	43.1
Siluriformes	524	36.5	588	39.3
Cichliformes	115	8.0	94	6.3
Gymnotiformes	74	5.2	78	5.2
Cyprinodontiformes	35	2.4	42	2.8
Clupeiformes	11	0.8	10	0.7
Myliobatiformes	9	0.6	11	0.7
Polycentridae and Sciaenidae (included in Perciformes in Maldonado-Ocampo et al. 2008)	7	0.5	8	0.5
Pleuronectiformes	7	0.5	5	0.3
Beloniformes	5	0.3	4	0.3
Batrachoidiformes	3	0.2	3	0.2
Osteoglossiformes	3	0.2	3	0.2
Eleotridae (included in Perciformes in Maldonado-Ocampo et al. 2008)	2	0.1	1	0.1
Synbranchiformes	1	0.1	1	0.1
Tetraodontiformes	1	0.1	1	0.1
Ceratodontiformes (Lepidosireniformes in Maldonado-Ocampo et al. 2008)	1	0.1	1	0.1
	1435	100	1494	100

**Table 2.** Number of species by hydrographic system (HS) in Maldonado-Ocampo et al. (2008) compared to the present work.

HS	Number of species in Maldonado-Ocampo et al. (2008)	Number of species in the present work	Number of species in Maldonado-Ocampo et al. (2008) with distribution changes
Amazon	788	706	58
Orinoco	658	663	54
Magdalena-Cauca	213	220	40
Caribbean	186	223	42
Pacific	151	130	30

### New records of species for Colombia

The high number of species already described from other countries but newly recorded for Colombia is a direct result of recent inventories in previously unsampled regions (e.g. Lasso et al. 2009, Villa-Navarro et al. 2011, Ortega-Lara et al. 2012) and perhaps equally or more important, from the verification of the taxonomic identity of records of doubtful species listed in Maldonado-Ocampo et al. (2008), as well as suspicious identifications (i.e., names of species distributed in river basins distant or isolated from the Colombian territory), found in the databases of the examined collections. Thus, the number of new records for the Colombian continental ichthyofauna reaches 114 species (see full list in Suppl. material 3, where also respective hydrographic zone and supporting lot and/or bibliographic reference are provided).

### Corrections and additions to the geographic distribution of species listed in Maldonado-Ocampo et al. (2008)

The original distribution of 175 species was modified (Suppl. material 4), with 39 species having wider distributions (i.e., encompassing at least one additional hydrographic system than previously recorded), 128 species with more restricted distributions, and 8 species with wrong distributions. At the end of the checklist are summarized the total number of each type of modification (addition or deletion) by hydrographic system.

A special treatment was implemented for the species of the genera *Astroblepus* and *Trichomycterus* that were originally described from Colombia. Most species of *Astroblepus* have restricted geographic distributions, being limited to portions of single river drainages at elevations above 1000 m (Schaefer et al. 2011). A similar scenario is also found for *Trichomycterus* species, which show high endemism and restricted geographic distributions to headwaters and streams of river systems (Fernández and Vari 2009, Ferrer and Malabarba 2013, DoNascimiento et al. 2014b). The taxonomic knowledge of the Colombian species of these genera and their respective geographic distributions is precarious. This uncertainty has resulted in misidentifications of specimens that ultimately result in misplacing species in different disjunct hydrographic zones, without any consideration of the ecological constraints for the geographic distribution of a certain taxonomic group or its biogeographic history. Based on these considerations, we circumscribed the geographic distribution of these species to the hydrographic zone that encompasses the river drainage from which the species were originally described.

### Species deleted from the Colombian continental ichthyofauna

A total of 202 nominal species originally listed in Maldonado-Ocampo et al. (2008) was removed from the updated checklist of the freshwater fishes of Colombia (numerical distribution of deleted species by orders and families is presented in the checklist). These species were found to be absent from Colombian freshwaters or were recently synonymized with other species. Each individual case of species removal is commented (Suppl. material 5) following the format of Lasso et al. (2004).

### Checklist of the native freshwater fishes of Colombia

Abbreviations: **DC** – described from Colombia. **EN** – endemic. **ET** – endangered and threatened species. **Amz** – Amazon River Basin. **Ori** – Orinoco River Basin. **Mag-Cau** – Magdalena-Cauca River Basin. **Pac** – Pacific slope rivers. **Car** – Caribbean slope rivers. Asterisk (*) denotes species newly recorded for Colombia after Maldonado-Ocampo et al. (2008). Two asterisks (**) indicate species inadvertently omitted in Maldonado-Ocampo et al. (2008). Plus (+) and minus (-) signs in the columns corresponding to individual hydrographic systems, indicate respectively, added or deleted presence in each hydrographic system, of species listed in Maldonado-Ocampo et al. (2008) with geographic distribution modified in the present checklist. **X** indicates unchanged presence in hydrographic systems already accounted in the above bibliographic reference.

**Table:** 

Taxa	DC	EN	ET	Amz	Ori	Mag-Cau	Pac	Car	References/Collections
**Myliobatiformes11 spp (3 added and 2 deleted from 2008)**									
**Potamotrygonidae**									
*Heliotrygon gomesi Carvalho & Lovejoy, 2011				X					Lasso et al. (2013a)
*Paratrygon aiereba* (Müller & Henle, 1841)			X	X	X				Maldonado-Ocampo et al. (2006a) | Lasso et al. (2013a)
*Plesiotrygon iwamae* Rosa, Castello & Thorson, 1987				X					Lasso et al. (2010)
*Plesiotrygon nana Carvalho & Ragno, 2011				X					Lasso et al. (2013a)
*Potamotrygon constellata* (Vaillant, 1880)				X					Bogotá-Gregory and Maldonado-Ocampo (2005)
*Potamotrygon magdalenae* (Duméril, 1865)	X	X	X			X		X	Duméril (1865) | Maldonado-Ocampo et al. (2006b) | MNHN A-2368
*Potamotrygon motoro* (Müller & Henle, 1841)			X	X	X				Mojica et al. (2005) | Maldonado-Ocampo et al. (2006a)
*Potamotrygon orbignyi* (Castelnau, 1855)			X	+	X				Lasso et al. (2013)
*Potamotrygon schroederi* Fernández-Yépez, 1958			X	X	X				Lasso et al. (2013)
*Potamotrygon scobina Garman, 1913			X	X	X				Lasso et al. (2013) | Acosta-Santos et al. (2016)
*Potamotrygon yepezi* Castex & Castello, 1970			X					X	Ortega-Lara et al. (2012)
**Osteoglossiformes3 spp**									
**Osteoglossidae**									
*Arapaima gigas* (Schinz 1822)			X	X					Mojica et al. (2005)
*Osteoglossum bicirrhosum* (Cuvier, 1829)			X	X	X				Mojica et al. (2005) | Maldonado-Ocampo et al. (2006a)
*Osteoglossum ferreirai* Kanazawa, 1966			X		X				Cala (1977)
**Clupeiformes10 spp (1 deleted from 2008)**									
**Pristigasteridae4 spp**									
*Ilisha amazonica* (Miranda Ribeiro, 1920)				X					Mojica et al. (2005)
*Pellona castelnaeana* Valenciennes, 1847				X	X				Correa (2003) | Maldonado-Ocampo (2001)
*Pellona flavipinnis* (Valenciennes, 1837)				X	X				Mojica et al. (2005) | Lasso et al. (2005) | ICN-MHN 733, 4144, 11309
*Pristigaster cayana* Cuvier, 1829				X					Mojica et al. (2005)
**Engraulidae6 spp (1 deleted from 2008)**									
*Amazonsprattus scintilla* Roberts, 1984					X				Maldonado-Ocampo and Bogotá-Gregory (2007)
*Anchoviella guianensis* (Eigenmann, 1912)				-	X				Galvis et al. (2007a)
*Anchoviella jamesi* (Jordan & Seale, 1926)				X	X				Kullander and Ferraris (2003)
*Jurengraulis juruensis* (Boulenger, 1898)				X					Mojica et al. (2005)
*Lycengraulis batesii* (Günther, 1868)				X	X				Mojica et al. (2005) | Kullander and Ferraris (2003)
*Pterengraulis atherinoides* (Linnaeus, 1766)					X				Galvis et al. (2007a)
**Characiformes644 spp (92 added and 93 deleted from 2008)**									
**Crenuchidae28 spp (2 added and 2 deleted from 2008)**									
*Crenuchus spilurus* Günther, 1863				X	X				Arbeláez et al. (2004) | Galvis et al. (2007a)
*Poecilocharax weitzmani* Géry, 1965				X	X				IAvH-P 10679
*Ammocryptocharax elegans* Weitzman & Kanazawa, 1976					X				Weitzman and Kanazawa (1976) | FMNH 80401 | USNM 210692, 214364
*Ammocryptocharax minutus* Buckup, 1993				X					Mojica et al. (2005)
*Characidium boavistae* Steindachner, 1915					+	X		+	Urbano-Bonilla et al. (2009); Ortega-Lara et al. (2012) | Villa-Navarro et al. (2006)
*Characidium caucanum* Eigenmann, 1912	X	X	X			X			Eigenmann (1912) | FMNH 56057 [ex CM 4847], 56058-60, 69546 | CAS 41275-77 [ex IU 12701-03] | USNM 79183
*Characidium chupa* Schultz, 1944					X			+	Urbano-Bonilla et al. (2009); Ortega-Lara et al. (2012)
*Characidium etheostoma* Cope, 1872				X					Mojica et al. (2005)
*Characidium longum* Taphorn, Montaña & Buckup, 2006					X				Lasso et al. (2009) | IAvH-P 2753, 13756, 13790
*Characidium pellucidum* Eigenmann, 1909				X	X				Mojica et al. (2005) | Maldonado-Ocampo et al. (2006a)
*Characidium phoxocephalum* Eigenmann, 1912	X	X	X			X			Eigenmann (1912) | FMNH 56061 [ex CM 4851] | CAS 60254 [ex IU 12704]
*Characidium pteroides* Eigenmann, 1909				X	X				Maldonado-Ocampo et al. (2008) | Maldonado-Ocampo et al. (2006a)
*Characidium roesseli* Géry, 1965				X					Mojica et al. (2005)
*Characidium sanctjohanni* Dahl, 1960	X	X					X	-	Dahl (1960c) | Agudelo-Zamora et al. (2014)
*Characidium steindachneri* Cope, 1878				X	X				Urbano-Bonilla et al. (2009). | Bogotá-Gregory and Maldonado-Ocampo (2006a) | CAS-SU 50488, 50655
*Characidium zebra* Eigenmann, 1909				X	X				Arbeláez et al. (2004) | Maldonado-Ocampo (2001)
*Elachocharax geryi* Weitzman & Kanazawa, 1978					X				Weitzman and Kanazawa (1978)
*Elachocharax mitopterus Weitzman, 1986				X					CZUT-IC 4071, 4086, 5106
*Elachocharax pulcher* Myers, 1927				X	X				Mojica et al. (2005) | Weitzman and Kanazawa (1978)
*Klausewitzia* Géry,1965				X					Bogotá-Gregory and Maldonado-Ocampo (2005)
*Leptocharacidium omospilus* Buckup, 1993					X				IAvH-P 13758
*Melanocharacidium dispilomma* Buckup, 1993				-	X				Buckup (1993)
*Melanocharacidium nigrum Buckup, 1993				X					IAvH-P 8660, 8700, 8765, 8796, 8859, 9421
*Melanocharacidium pectorale* Buckup, 1993				X	X				Buckup (1993) | Mojica et al. (2005)
*Microcharacidium eleotrioides* (Géry, 1960)					X				Maldonado-Ocampo et al. (2008)
*Microcharacidium gnomus* Buckup, 1993					X				Buckup (1993)
*Microcharacidium weitzmani* Buckup, 1993					X				Buckup (1993)
*Odontocharacidium aphanes* (Weitzman & Kanazawa, 1977)				X					Buckup (1993)
**Erythrinidae4 spp (1 added and 1 deleted from 2008)**									
*Erythrinus erythrinus* (Bloch & Schneider, 1801)				X	X				Eigenmann (1922) | Fowler (1945) | ICN-MHN 440, 3801
*Hoplerythrinus unitaeniatus* (Agassiz, 1829)				X	X				Fowler (1945) | Cala (1977)
*Hoplias curupira Oyakawa & Mattox 2009					X				IAvH-P 1349, 2274, 2407, 2743
*Hoplias malabaricus* (Bloch, 1794)				X	X	X	X	X	Fowler (1943) | Steindachner (1879a) | Eigenmann (1922) | Maldonado-Ocampo et al. (2013b) | Regan (1914)
**Parodontidae9 spp (3 added from 2008)**									
*Parodon alfonsoi* Londoño-Burbano, Román-Valencia & Taphorn, 2011	X	X				X			Londoño-Burbano et al. (2011)
*Parodon apolinari* Myers, 1930					X				Myers (1930)
*Parodon atratoensis* Londoño-Burbano, Román-Valencia & Taphorn, 2011	X	X						X	Londoño-Burbano et al. (2011)
*Parodon buckleyi* Boulenger, 1887				X					Londoño-Burbano et al. (2011)
*Parodon caliensis* Boulenger, 1895	X	X	X			X			Boulenger (1895)
*Parodon magdalenensis* Londoño-Burbano, Román-Valencia & Taphorn, 2011	X	X				X			Londoño-Burbano et al. (2011)
*Parodon pongoensis* (Allen, 1942)				X					Londoño-Burbano et al. (2011)
*Parodon suborbitalis* Valenciennes, 1850						-		X	Londoño-Burbano et al. (2011) | Eigenmann (1922)
*Saccodon dariensis* (Meek & Hildebrand, 1913)			X			X		X	Pavanelli and Starnes (2015)
**Cynodontidae7 spp**									
*Cynodon gibbus* Spix & Agassiz, 1829				X	X				Mojica et al. (2005) | Toledo-Piza (2000a)
*Cynodon septenarius* Toledo-Piza, 2000					X				Lasso et al. (2005) | IAvH-P 1043, 1053, 3237
*Hydrolycus armatus* (Jardine & Schomburgk, 1841)				X	X				Bogotá-Gregory and Maldonado-Ocampo (2005) | Maldonado-Ocampo et al. (2006a)
*Hydrolycus scomberoides* (Cuvier, 1816)				X					Mojica et al. (2005)
*Hydrolycus tatauaia* Toledo-Piza, Menezes & Santos, 1999					X				IAvH-P 9915
*Hydrolycus wallacei* Toledo-Piza, Menezes & Santos, 1999				X	X				Bogotá-Gregory and Maldonado-Ocampo (2005) | Maldonado-Ocampo et al. (2006a)
*Rhaphiodon vulpinus* Spix & Agassiz, 1829				X	X				Mojica et al. (2005) | Cala (1977)
**Serrasalmidae39 spp (1 added and 4 deleted from 2008)**									
*Catoprion mento* (Cuvier, 1819)				X	X				Bogotá-Gregory and Maldonado-Ocampo (2006a) | Lasso et al. (2005) | IAvH-P 751, 779,814, 961, 1130, 1173, 1385, 2436, 4911, 5026, 5917, 11272, 12771 | ICN-MHN 5246, 5664
*Colossoma macropomum* (Cuvier, 1818)			X	X	X				Bogotá-Gregory and Maldonado-Ocampo (2006a) | Cala (1977) | IAvH-P 2031, 5898, 10549, 11601, 11607
*Metynnis argenteus* Ahl, 1923				X	X				Galvis et al. (2007b) | IAvH-P 1399, IAvH-P 11260, IAvH-P 13864
*Metynnis hypsauchen* (Müller & Troschel, 1844)				X	X				Bogotá-Gregory and Maldonado-Ocampo (2005) | Maldonado-Ocampo and Bogotá-Gregory (2007)
*Metynnis lippincottianus* (Cope, 1870)				X	X				Bogotá-Gregory and Maldonado-Ocampo (2005) | Maldonado-Ocampo (2001)
*Metynnis luna* Cope, 1878				X	X				Correa (2003) | Maldonado-Ocampo (2001)
*Myleus rhomboidalis* (Cuvier, 1818)				X					Bogotá-Gregory and Maldonado-Ocampo (2005)
*Myleus setiger* Müller & Troschel, 1844				X	X				Bogotá-Gregory and Maldonado-Ocampo (2005) | Maldonado-Ocampo et al. (2006a)
*Myleus torquatus* (Kner, 1858)				X	X				Mojica (1999) | Maldonado-Ocampo (2001) | ICN-MHN 2557, 3426, 3838
*Myloplus asterias* (Müller & Troschel, 1844)				X	-				Correa (2003)
*Myloplus rubripinnis* (Müller & Troschel, 1844)				X	X				Mojica et al. (2005) | Cala (1977)
*Myloplus schomburgkii* (Jardine, 1841)				X	X				Bogotá-Gregory and Maldonado-Ocampo (2005) | Cala (1977)
*Mylossoma acanthogaster* (Valenciennes, 1850)			X					X	Usma et al. (2002)
*Mylossoma aureum* (Spix & Agassiz, 1829)				X	X				Mojica et al. (2005)
*Mylossoma duriventre* (Cuvier, 1818)				X	X				Mojica et al. (2005) | Cala (1977)
*Piaractus brachypomus* (Cuvier, 1818)				X	X				Mojica et al. (2005) | Cala (1977)
*Pristobrycon aureus* (Spix & Agassiz, 1829)				X					Bogotá-Gregory and Maldonado-Ocampo (2006a) | ICN-MHN 5251
*Pristobrycon calmoni* (Steindachner, 1908)				X	X				Mojica et al. (2005)
*Pristobrycon careospinus* Fink & Machado-Allison, 1992				X	X				Bogotá-Gregory and Maldonado-Ocampo (2005) | Maldonado-Ocampo et al. (2006a)
*Pristobrycon maculipinnis* Fink & Machado-Allison, 1992				X					Correa (2003)
*Pristobrycon striolatus* (Steindachner, 1908)				X	X				Correa (2003) | Maldonado-Ocampo et al. (2006a)
*Pygocentrus cariba* (Humboldt & Valenciennes, 1821)					X				Maldonado-Ocampo (2001)
*Pygocentrus nattereri* Kner, 1858				X	X				Mojica et al. (2005)
*Pygopristis denticulata* (Cuvier, 1819)					X				Maldonado-Ocampo et al. (2006a)
*Serrasalmus altuvei* Ramírez, 1965				X	X				Correa (2003) | Maldonado-Ocampo (2001)
*Serrasalmus compressus* Jégu, Leão & Santos, 1991				X					Galvis et al. (2007b)
*Serrasalmus eigenmanni Norman, 1929				X					CZUT-IC 7334
*Serrasalmus elongatus* Kner, 1858				X					Mojica et al. (2005)
*Serrasalmus gouldingi* (Fink & Machado-Allison, 1992)				X					Bogotá-Gregory and Maldonado-Ocampo (2006a) | IAvH-P 262
*Serrasalmus hollandi* Eigenmann, 1915				X					Mojica et al. (2005)
*Serrasalmus humeralis* Valenciennes, 1850				X					Bogotá-Gregory and Maldonado-Ocampo (2006a) | ICN-MHN 5255, 6108
*Serrasalmus irritans* Peters, 1877				-	X				Maldonado-Ocampo (2001)
*Serrasalmus maculatus* Kner, 1858				X					Jégu and Dos Santos (2001)
*Serrasalmus manueli* (Fernández-Yépez & Ramírez, 1967)				X	X				Galvis et al. (2007b) | Maldonado-Ocampo (2001)
*Serrasalmus medinai* Ramírez, 1965				X	X				Mojica et al. (2005)
*Serrasalmus nalseni* Fernández-Yépez, 1969				X					Correa (2003)
*Serrasalmus rhombeus* (Linnaeus, 1766)				X	X				Mojica et al. (2005)
*Serrasalmus sanchezi* Géry, 1964				X					IAvH-P 10526-10528, 10539, 10554-10556, 10559, 11543-11552, 11585
*Serrasalmus spilopleura* Kner, 1858				X	X				Mojica et al. (2005) | Galvis et al. (2007a)
**Hemiodontidae14 spp (1 added and 1 deleted from 2008)**									
*Anodus elongatus* Agassiz, 1829				X					Mojica et al. (2005)
*Anodus orinocensis* (Steindachner, 1887)					X				Zapata and Usma (2013) | CUZT-IC 3450, 4062
*Hemiodus amazonum* (Humboldt, 1821)				+	-				ICN-MHN 17168
*Hemiodus argenteus* Pellegrin, 1909				X	X				Galvis et al. (2007b) | Galvis et al. (2007a)
*Hemiodus atranalis (Fowler, 1940)				X					IAvH-P 2389
*Hemiodus gracilis* Günther, 1864				-	X				Maldonado-Ocampo et al. (2006a)
*Hemiodus immaculatus* Kner, 1858				X	X				Galvis et al. (2007b) | Maldonado-Ocampo et al. (2006a)
*Hemiodus microlepis* Kner, 1858				X	X				Arbeláez et al. (2004) | Lasso et al. (2005) | IMCN 606
*Hemiodus semitaeniatus* Kner, 1858				X	X				Galvis et al. (2007b) | Cala (1977)
*Hemiodus thayeria* Böhlke, 1955				X	+				Böhlke (1955) | Galvis et al. (2007b) | Ortega-Lara (2016)
*Hemiodus unimaculatus* (Bloch, 1794)				X	X				Galvis et al. (2007b) | Cala (1977)
*Hemiodus vorderwinkleri* (Géry, 1964)				X					Géry (1964b)
*Argonectes longiceps* (Kner, 1858)				X	X				Maldonado-Ocampo et al. (2006a) | IAvH-P 5746
*Bivibranchia fowleri* (Steindachner, 1908)				X	X				Galvis et al. (2007b) | Maldonado-Ocampo et al. (2006a)
**Anostomidae43 spp (4 added and 14 deleted from 2008)**									
*Abramites eques* (Steindachner, 1878)	X	X	X			X			Steindachner (1878) | BMNH 1895.5.17.155-156 | NMW 69549, 69548, 69550
*Abramites hypselonotus* (Günther, 1868)				X	X				Lasso et al. (2005) | Mojica et al. (2005) | IAvH-P 3411
*Anostomoides laticeps (Eigenmann, 1912)				X					XIII Congreso Colombiano de Ictiología
*Anostomus anostomus* (Linnaeus, 1758)				X	X				Fowler (1943) | Cala (1977) | IAvH-P 1014, 2830
*Anostomus ternetzi* Fernández-Yépez, 1949				-	X				Maldonado-Ocampo et al. (2006a)
*Gnathodolus bidens* Myers, 1927					X				Maldonado-Ocampo et al. (2006a)
*Laemolyta fernandezi* Myers, 1950					X				Galvis et al. (2007a)
*Laemolyta garmani* (Borodin, 1931)				X					Mojica et al. (2005)
*Laemolyta proxima* (Garman, 1890)				X					Mojica et al. (2005)
*Laemolyta taeniata* (Kner, 1858)				X	X				Mautari and Menezes (2006) | Mojica et al. (2005)
*Leporellus vittatus* (Valenciennes, 1850)					X	X		+	Steindachner (1880) | Garavello and Britski (2003) | Maldonado-Ocampo et al. (2005) | Dahl (1955)
*Leporinus affinis* Günther, 1864				X					Garavello and Britski (2003)
*Leporinus agassizi* Steindachner, 1876				X					Mojica et al. (2005)
*Leporinus amazonicus Santos & Zuanon, 2008				X					XIII Congreso Colombiano de Ictiología
*Leporinus aripuanensis* Garavello & Santos, 1981				X					Mojica et al. (2005)
*Leporinus bimaculatus* Castelnau, 1855				X					Mojica et al. (2005)
*Leporinus boehlkei* Garavello, 1988	X	X			X				Garavello (1988) | ANSP 136487, 135432-33, 136489, 138803, 140316 | MZUSP 28061
*Leporinus brunneus* Myers, 1950				X	X				Galvis et al. (2007b) | Maldonado-Ocampo et al. (2006a)
*Leporinus enyae* Burns, Chatfield, Birindelli & Sidlauskas, 2017					X				Burns et al. (2017)
*Leporinus fasciatus* (Bloch, 1794)				X	X				Correa (2003) | Maldonado-Ocampo et al. (2006a)
*Leporinus friderici* (Bloch, 1794)				X	X				Mojica et al. (2005) | Maldonado-Ocampo et al. (2006a)
*Leporinus granti* Eigenmann, 1912					X				Lasso et al. (2009) | ICN-MHN 948, 1301
*Leporinus jamesi* Garman, 1929				X					Garavello et al. (2014)
*Leporinus klausewitzi* Géry, 1960				X					Galvis et al. (2007b)
*Leporinus moralesi* Fowler, 1942				X					Bogotá-Gregory and Maldonado-Ocampo (2005)
*Leporinus nattereri* Steindachner, 1876				X	X				Maldonado-Ocampo et al. (2006a) | IAvH-P 8822
*Leporinus niceforoi* Fowler, 1943	X	X		X					Fowler (1943) | ANSP 70491, 70492
*Leporinus ortomaculatus* Garavello, 2000					X				IAvH-P 13843
*Leporinus parae Eigenmann, 1907				X	X				IAvH-P 1822, 12770, 13003, 13011
*Leporinus subniger* Fowler, 1943				X					Fowler (1943) | IAvH-P 3430-3432 | ANSP 70493, 70494
*Leporinus striatus* Kner, 1858				+	X	X	X	X	Galvis et al. (2007b) | Urbano-Bonilla et al. (2009). | Steindachner (1879a) | Eigenmann (1922) | Regan (1914)
*Leporinus wolfei* Fowler, 1940				X					Mojica et al. (2005)
*Leporinus y-ophorus* Eigenmann, 1922					X				Eigenmann (1922) | CAS 61680
*Megaleporinus muyscorum* (Steindachner, 1900)	X	X	X			X		X	Steindachner (1900) | Eigenmann (1922)
*Megaleporinus trifasciatus* (Steindachner, 1876)				X					Mojica et al. (2005)
*Pseudanos trimaculatus* Kner, 1858				X					Mojica et al. (2005)
*Pseudanos winterbottomi* Sidlauskas & Santos, 2005					X				Maldonado-Ocampo et al. (2006a)
*Rhytiodus argenteofuscus* Kner, 1858				X					Mojica et al. (2005)
*Rhytiodus microlepis* Kner, 1858				X					Mojica et al. (2005)
*Schizodon corti* Schultz, 1944								X	Ortega-Lara et al. (2012)
*Schizodon fasciatus* Spix & Agassiz, 1829				X					Mojica et al. (2005)
*Schizodon scotorhabdotus* Sidlauskas, Garavello & Jellen, 2007					X				Sidlauskas et al. (2007)
*Synaptolaemus latofasciatus* (Steindachner, 1910)				X	X				Maldonado-Ocampo et al. (2006a) | CZUT-IC 4092
**Chilodontidae5 spp (1 added and 1 deleted from 2008)**									
*Caenotropus labyrinthicus* (Kner, 1858)				X	X				Cala (1977) | Vari et al. (1995)
*Caenotropus mestomorgmatos* Vari, Castro & Raredon, 1995				X	+				Bogotá-Gregory and Maldonado-Ocampo (2006a) | IAvH-P 281, 4098, 4101
*Caenotropus schizodon Scharcansky & Lucena, 2007				X					CZUT-IC 12280
*Chilodus gracilis* Isbrücker & Nijssen, 1988				X	+				Bogotá-Gregory and Maldonado-Ocampo (2006a) | USNM 190300, 232361, 263288, 431461
*Chilodus punctatus* Müller & Troschel, 1844				X	X				Mojica et al. (2005) | Cala (1977)
**Curimatidae47 spp (2 added and 2 deleted from 2008)**									
*Curimata aspera* (Günther, 1868)				X					Mojica et al. (2005)
*Curimata cerasina* Vari, 1984				-	X				CZUT-IC 11494
*Curimata cisandina* (Allen, 1942)				X					Mojica et al. (2005)
*Curimata cyprinoides* (Linnaeus, 1766)				X					Bogotá-Gregory and Maldonado-Ocampo (2006a)
*Curimata incompta* Vari, 1984				X	X				Mojica et al. (2005) | Maldonado-Ocampo (2001) | IAvH-P 4370
*Curimata mivartii* (Steindachner, 1878)	X	X	X			X		+	Steindachner (1878) | Vari (1989a)
*Curimata ocellata* (Eigenmann & Eigenmann, 1889)				X					Mojica et al. (2005)
*Curimata roseni* Vari, 1989				X					Bogotá-Gregory and Maldonado-Ocampo (2006a)
*Curimata vittata* (Kner, 1858)				X	X				Vari (1989a) | Maldonado-Ocampo (2001)
*Curimatella alburna* (Müller & Troschel, 1844)				X					Arbeláez et al. (2004)
*Curimatella dorsalis* (Eigenmann & Eigenmann, 1889)				X	X				Mojica et al. (2005); Maldonado-Ocampo (2001)
*Curimatella immaculata* (Fernández-Yépez, 1948)				X	X				Vari (1992b) | Bogotá-Gregory and Maldonado-Ocampo (2006a)
*Curimatella meyeri* (Steindachner, 1882)				X					Mojica et al. (2005)
*Curimatopsis crypticus* Vari, 1982					X				IAvH-P 13266
*Curimatopsis evelynae* Géry, 1964					X				Géry (1964a)
*Curimatopsis macrolepis* (Steindachner, 1876)				X	X				Vari (1982)
*Curimatopsis microlepis Eigenmann & Eigenmann, 1889				X					CZUT-IC 4073, 4075
*Cyphocharax abramoides* (Kner, 1858)					X				Vari (1992a)
*Cyphocharax aspilos* Vari, 1992								X	Ortega-Lara et al. (2012)
*Cyphocharax festivus* Vari, 1992				X	X				Bogotá-Gregory and Maldonado-Ocampo (2006a) | Maldonado-Ocampo et al. (2006a)
*Cyphocharax leucostictus* (Eigenmann & Eigenmann, 1889)				X					Galvis et al. (2007b)
*Cyphocharax magdalenae* (Steindachner, 1878)						X		X	Steindachner (1878) | Eigenmann (1922) | Vari (1992a)
*Cyphocharax multilineatus* (Myers, 1927)				X	X				Galvis et al. (2007b) | Galvis et al. (2007a)
*Cyphocharax nigripinnis* Vari, 1992				X					Vari (1992a)
*Cyphocharax notatus (Steindachner, 1908)				X					CZUT-IC 10874
*Cyphocharax oenas* Vari, 1992					X				Maldonado-Ocampo et al. (2006a)
*Cyphocharax pantostictos* Vari & Barriga, 1990				X					IAvH-P 8354-8357
*Cyphocharax spiluropsis* (Eigenmann & Eigenmann, 1889)				X					Arbeláez et al. (2004)
*Cyphocharax spilurus* (Günther, 1864)				X	X				Mojica et al. (2005) | Cala (1977)
*Potamorhina altamazonica* (Cope, 1878)				X	X				Vari (1984)
*Potamorhina laticeps* (Valenciennes, 1850)								X	Ortega-Lara et al. (2012)
*Potamorhina latior* (Spix & Agassiz, 1829)				X					Vari (1984)
*Potamorhina pristigaster* (Steindachner, 1876)				X					Bogotá-Gregory and Maldonado-Ocampo (2006a)
*Psectrogaster amazonica* Eigenmann & Eigenmann, 1889				X					Mojica et al. (2005)
*Psectrogaster ciliata* (Müller & Troschel, 1844)					X				Vari (1989b)
*Psectrogaster essequibensis* (Günther, 1864)				X					Mojica et al. (2005)
*Psectrogaster rhomboides* Eigenmann & Eigenmann, 1889				X					Mojica et al. (2005)
*Psectrogaster rutiloides* (Kner, 1858)				X					Vari (1989b)
*Pseudocurimata lineopunctata* (Boulenger, 1911)							X	X	Boulenger (1911) | Eigenmann (1922)
*Pseudocurimata patiae* (Eigenmann, 1914)	X	X	X				X		Eigenmann et al. (1914) | FMNH 56554, 56555 | CAS 60622 [ex IU 13055]
*Steindachnerina argentea* (Gill, 1858)				-	X				Eigenmann (1922)
*Steindachnerina atratoensis* (Eigenmann, 1912)	X	X						X	Eigenmann (1912) | FMNH 56024 [ex CM 4814a], 56043, 71308 | CAS 44218 [ex IU 12676] | MCZ 30933 | USNM 79192
*Steindachnerina bimaculata* (Steindachner, 1876)				X					Vari (1991)
*Steindachnerina dobula* (Günther, 1868)				X					Vari (1991)
*Steindachnerina guentheri* (Eigenmann & Eigenmann, 1889)				X	X				Arbeláez et al. (2004) | Vari (1991)
*Steindachnerina hypostoma* (Boulenger, 1887)				X					Mojica et al. (2005)
*Steindachnerina pupula* Vari, 1991					X				Vari (1991)
**Prochilodontidae10 spp**									
*Ichthyoelephas longirostris* (Steindachner, 1879)	X	X	X			X		+	Steindachner (1879b) | Castro and Vari (2004)
*Prochilodus magdalenae* Steindachner, 1879	X	X	X			X		X	Steindachner (1879a) | Eigenmann (1922)
*Prochilodus mariae* Eigenmann, 1922					X				Eigenmann (1922)
*Prochilodus nigricans* Spix & Agassiz, 1829				X					Fowler (1943)
*Prochilodus reticulatus* Valenciennes, 1850			X					X	Ortega-Lara et al. (2012)
*Prochilodus rubrotaeniatus* Jardine & Schomburgk, 1841				X	X				Bogotá-Gregory and Maldonado-Ocampo (2006a) | Urbano-Bonilla et al. (2009). | IAvH-P 5844, 6195, 10879, 10880, 12764
*Semaprochilodus insignis* (Jardine & Schomburgk, 1841)				X	X				Mojica et al. (2005) | Castro and Vari (2004)
*Semaprochilodus kneri* (Pellegrin, 1909)				X	X				Correa (2003) | Maldonado-Ocampo et al. (2006a)
*Semaprochiloduslaticeps (Steindachner, 1879)*					X				Cala (1977) | Castro and Vari (2004)
*Semaprochilodus taeniurus* (Valenciennes, 1821)				X					Correa (2003)
**Lebiasinidae30 spp (3 added and 10 deleted from 2008)**									
*Lebiasina astrigata* Regan, 1913								X	Maldonado-Ocampo et al. (2006b)
*Lebiasina chocoensis* Ardila Rodríguez, 2010	X	X						X	Ardila Rodríguez (2010)
*Lebiasina chucuriensis* Ardila Rodríguez, 2001	X	X				X			Ardila Rodríguez (2001) | CAR 150424, 150423, 150425
*Lebiasina colombia* Ardila Rodríguez, 2008	X	X						X	Ardila Rodríguez (2008b) | CAR 190, 191, 192 | ICN-MHN 5314
*Lebiasina elongata* (Boulenger, 1887)				X					Galvis et al. (2007b)
*Lebiasina erythrinoides* (Valenciennes, 1850)				X	X	X	X	+	Maldonado-Ocampo et al. (2005) | Lasso et al. (2005) | Bogotá-Gregory and Maldonado-Ocampo (2005) | Ortega-Lara et al. (2012) | IAvH-P 573
*Lebiasina festae* Boulenger, 1899							X	X	Maldonado-Ocampo et al. (2013b) | Fowler (1939)
*Lebiasina floridablancaensis* Ardila Rodríguez, 1994	X	X				X			Ardila Rodríguez (1994) | CAR 15-04-01 | MBUCV-V-26713 [ex CAR 15-04-02 and 03] | MHNUNC 1837
*Lebiasina multimaculata* Boulenger, 1911	X	X					X		Boulenger (1911) | BMNH 1910.7.11.167-169
*Lebiasina narinensis* Ardila Rodríguez, 2002	X	X				-	X		Ardila Rodríguez (2002) | ICN-MHN 2340, 2340-1
*Lebiasina ortegai* Ardila Rodríguez, 2008	X	X				X			Ardila Rodríguez (2008a) | CAR 157, 265 | CZUT-IC 2586 | IAvH-P 9875 | IMCN 4200, 4201
*Lebiasina panamensis* Gill, 1877							X	X	Eigenmann (1922) | Fowler (1944)
*Copeina guttata* (Steindachner, 1876)				X					Steindachner (1876)
*Copella eigenmanni* (Regan, 1912)					X				Regan (1912d)
*Copella nattereri* (Steindachner, 1876)				X	X				Arbeláez et al. (2004) | Galvis et al. (2007a)
*Copella vilmae* Géry, 1963				X					Mojica et al. (2005)
*Pyrrhulina brevis* Steindachner, 1876				X	X				Correa (2003) | Urbano-Bonilla et al. (2009).
*Pyrrhulina eleanorae* Fowler, 1940					X				Maldonado-Ocampo et al. (2008)
*Pyrrhulina filamentosa* Valenciennes, 1847					X				Lasso et al. (2005) | IAvH-P 1589, 3405
*Pyrrhulina laeta* (Cope, 1872)				X					Arbeláez et al. (2004)
*Pyrrhulina lugubris* Eigenmann, 1922				-	X				Eigenmann (1922) | CAS 78888 [ex IU 15041a], 78888 [ex IU 15041]
*Pyrrhulina obermulleri* Myers, 1926				X					Mojica et al. (2005)
*Pyrrhulina semifasciata* Steindachner, 1876				X					Bogotá-Gregory and Maldonado-Ocampo (2006a) | USNM 311003
*Nannostomus digrammus (Fowler, 1913)				X					IAvH-P 10688, 10692, 10694
*Nannostomus eques* Steindachner, 1876				X	X				Mojica et al. (2005) | Maldonado-Ocampo et al. (2006a)
*Nannostomus harrisoni* (Eigenmann, 1909)				X	X				Bogotá-Gregory and Maldonado-Ocampo (2005) | Maldonado-Ocampo et al. (2006a)
*Nannostomus marginatus* Eigenmann, 1909				X	X				Weitzman (1966) | Lasso et al. (2005)
*Nannostomus marilynae* Weitzman & Cobb, 1975				X	X				Weitzman and Cobb (1975)
*Nannostomus trifasciatus* Steindachner, 1876				X	X				Arbeláez et al. (2004) | Galvis et al. (2007a)
*Nannostomus unifasciatus* Steindachner, 1876				X	X				Arbeláez et al. (2004) Maldonado-Ocampo et al. (2006a)
**Ctenoluciidae7 spp**									
*Boulengerella cuvieri* (Agassiz, 1829)				X	X				Vari (1995)
*Boulengerella lateristriga* (Boulenger, 1895)				X	X				Correa (2003) | Maldonado-Ocampo et al. (2006a)
*Boulengerella lucius* (Cuvier, 1816)				-	X				Maldonado-Ocampo et al. (2006a)
*Boulengerella maculata* (Valenciennes, 1850)				X	X				Vari (1995) | Cala (1977)
*Boulengerella xyrekes* Vari, 1995				X	X				Bogotá-Gregory and Maldonado-Ocampo (2006a) | Maldonado-Ocampo et al. (2006a) | IAvH-P 1789, 2044, 2851
*Ctenolucius beani* (Fowler, 1907)							X	X	Fowler (1907) | Vari (1995)
*Ctenolucius hujeta* (Valenciennes, 1850)						X		X	Vari (1995) | Bean (1908)
**Acestrorhynchidae18 spp (1 added and 1 deleted from 2008)**									
*Acestrorhynchus abbreviatus* (Cope, 1878)				X					Galvis et al. (2007b)
*Acestrorhynchus falcatus* (Bloch, 1794)				X	X				Galvis et al. (2007b) | Eigenmann (1922)
*Acestrorhynchus falcirostris* (Cuvier, 1819)				X	X				Mojica et al. (2005) | Maldonado-Ocampo et al. (2006a)
*Acestrorhynchus grandoculis* Menezes & Géry, 1983					X				Maldonado-Ocampo et al. (2006a)
*Acestrorhynchus heterolepis* (Cope, 1878)				X	X				Lasso et al. (2005) | Galvis et al. (2007b) | IAvH-P 834, 918, 2157, 10772
*Acestrorhynchus microlepis* (Schomburgk, 1841)				X	X				Mojica et al. (2005) | Toledo-Piza and Menezes (1996) | Cala (1977)
*Acestrorhynchus minimus* Menezes, 1969					X				Maldonado-Ocampo et al. (2006a)
*Acestrorhynchus nasutus* Eigenmann, 1912					X				Lasso et al. (2009) | IAvH-P 1124, 9908, 9909, 9910, 9911, 9912, 9913, 9914, 13688, 13797, 13875 | ICN-MHN 1280
*Gnathocharax steindachneri* Fowler, 1913					X				Cala (1977)
*Heterocharax macrolepis* Eigenmann, 1912				X	X				Toledo-Piza (2000b) | IAvH-P 10658
*Heterocharax virgulatus Toledo-Piza, 2000					X				IAvH-P 14091
*Hoplocharax goethei* Géry, 1966					X				IAvH-P 15984
*Lonchogenys ilisha* Myers, 1927				X	X				Mojica et al. (2005) | Maldonado-Ocampo et al. (2006a)
*Gilbertolus alatus* (Steindachner, 1878)	X	X				X		+	Steindachner (1878) | Eigenmann (1922)
*Gilbertolus atratoensis* Schultz, 1943	X	X						X	Schultz (1943) | USNM 76976, 120170
*Gilbertolus maracaiboensis* Schultz, 1943								X	Ortega-Lara et al. (2012)
*Roestes molossus* (Kner, 1858)				X					Prada-Pedreros (1997)
*Roestes ogilviei* (Fowler, 1914)				X					Galvis et al. (2007b)
**Characidae325 spp (68 added and 51 deleted from 2008)**									
*Brachychalcinus copei* (Steindachner, 1882)				X					Mojica et al. (2005)
*Brachychalcinus nummus* Böhlke, 1958				X					Bogotá-Gregory and Maldonado-Ocampo (2005)
*Brachychalcinus orbicularis* (Valenciennes, 1850)				X	X				Mojica et al. (2005) | ICN-MHN 1309 | 824, 839, 862, 865
*Gymnocorymbus bondi* (Fowler, 1911)					X				Benine et al. (2015)
*Gymnocorymbus thayeri* Eigenmann, 1908				X	-				Fowler (1945) | Galvis et al. (2007a)
*Poptella brevispina* Reis, 1989					X				IAvH-P 4382
*Poptella compressa* (Günther, 1864)				+	X				Mojica et al. (2005) | Maldonado-Ocampo and Bogotá-Gregory (2007)
*Poptella longipinnis* (Popta, 1901)					X				Maldonado-Ocampo et al. (2013a)
*Stethaprion erythrops* (Cope, 1870)				X					Galvis et al. (2007b)
*Stichonodon insignis* (Steindachner, 1876)				X					Mojica et al. (2005)
*Parastremma album* Dahl, 1960	X	X					X		Dahl (1960c) | ICN-MHN 147, 147a
*Parastremma pulchrum* Dahl, 1960	X	X					X	-	Dahl (1960c) | ICN-MHN 204
*Parastremma sadina* Eigenmann, 1912	X	X					X	X	Eigenmann (1912) | FMNH 56022 [ex CM 4812], 56023, 69678 | CAS 57600 [ex IU 12675] | USNM 79225
*Acestrocephalus anomalus* (Steindachner, 1880)	X	X	X			X			Steindachner (1880) | NMW 57983
*Acestrocephalus boehlkei* Menezes, 1977				X					Galvis et al. (2007b)
*Acestrocephalus sardina* (Fowler, 1913)				X	X				Bogotá-Gregory and Maldonado-Ocampo (2006a) | Lasso et al. (2005) | IAvH-P 4092-4093, 4793-4796,5851,12001, 12025, 12026, 12119
*Charax condei* (Géry & Knöppel, 1976)				X	X				Mojica et al. (2005)
*Charax metae* Eigenmann, 1922					X				Eigenmann (1922) | CAS 41300 [ex IU 15027], 69117 [ex IU 15027] | FMNH 55145 | IAvH-P 3566, 3567, 3569, 3571, 3573, 3574, 7732 | SU 60707 [ex IU 15028] | UMMZ 160226 | USNM 83631 [ex IU 15027]
*Charax michaeli* Lucena, 1989				X					Mojica et al. (2005)
*Charax niger* Lucena, 1989				X					Mojica et al. (2005)
*Charax notulatus Lucena, 1987					X				IAvH-P 7521, 13028, 13073
*Charax tectifer* (Cope, 1870)				X					Arbeláez et al. (2004)
*Cynopotamus amazonus* (Günther, 1868)				X					Mojica et al. (2005)
*Cynopotamus atratoensis* (Eigenmann, 1907)	X	X	X					X	Eigenmann and Ogle (1907) | USNM 1664, 306567 [ex USNM 1664]
*Cynopotamus bipunctatus* Pellegrin, 1909					X				Urbano-Bonilla et al. (2009).
*Cynopotamus magdalenae* (Steindachner, 1879)	X	X	X			X			Steindachner (1879a) | NMW 62501-02, 62504-05, 77769
*Cynopotamus venezuelae* (Schultz, 1944)								X	Ortega-Lara et al. (2012)
*Exodon paradoxus* Müller & Troschel, 1844					X				Cala (1977)
*Galeocharax gulo* (Cope, 1870)				X					Mojica et al. (2005)
*Phenacogaster maculoblonga de Lucena & Malabarba, 2010					X				CZUT-IC 9680
*Phenacogaster napoatilis de Lucena & Malabarba, 2010				X					CZUT-IC 9008
*Phenacogaster pectinata* (Cope, 1870)				X					Mojica et al. (2005)
*Phenacogaster prolata de Lucena & Malabarba, 2010					X				CZUT-IC 7223
*Priocharax pygmaeus* Weitzman & Vari, 1987	X	X		X					Weitzman and Vari (1987) | NRM 15048 | MBUCV-V-15342 | MZUSP 36498 | NRM 17593 | USNM 278479
*Roeboides affinis* (Günther, 1868)				X	X				Lucena (2007)
*Roeboides araguaito* Lucena, 2003					X				Lucena (2003)
*Roeboides dayi* (Steindachner, 1878)						X	X	X	Steindachner (1878) | Maldonado-Ocampo et al. (2013b)
*Roeboides dientonito* Schultz, 1944					X			+	Lasso et al. (2005) | Ortega-Lara et al. (2012) | IAvH-P 2935, 2988, 3329-3332, 4906, 9497, 13083, 13169, 13185, 13198, 13216, 13236, 13249, 13278, 13296, 13376
*Roeboides myersii* Gill, 1870				X	X				Mojica et al. (2005)
*Roeboides occidentalis* Meek & Hildebrand, 1916						-	X	-	Maldonado-Ocampo et al. (2013b)
*Bario steindachneri* (Eigenmann, 1893)				X					Galvis et al. (2006)
*Hemigrammus aguaruna* Lima, Correa & Ota, 2016				X					Lima et al. (2016) | Galvis et al. (2006)
*Hemigrammus analis* Durbin, 1909				X	X				Arbeláez et al. (2004) | Maldonado-Ocampo (2001)
*Hemigrammus barrigonae* Eigenmann & Henn, 1914					X				Eigenmann and Henn (1914) | AMNH 5320 | CAS 44368 [ex IU 13423], 44369 [ex IU 13424] | IAvH-P 3633
*Hemigrammus bellottii* (Steindachner, 1882)				X	X				Mojica et al. (2005) | Cala (1977) | IAvH-P 10304, 10307
*Hemigrammus bleheri* Géry & Mahnert, 1986					X				Géry and Mahnert (1986)
*Hemigrammus coeruleus* Durbin, 1908				X					Bogotá-Gregory and Maldonado-Ocampo (2006a) | ICN-MHN 5065
*Hemigrammus geisleri Zarske & Géry, 2007				X	X				CZUT-IC 4400, 5097, 8655, 8851, 8933, 8954, 9099, 9245, 9993, 14642, 16138, 16309, 16837, 16934, MPUJ 306, 1232, 1234, 1237, 1357, 4378, 4392, 10804
*Hemigrammus gracilis* (Lütken, 1875)				X	X				Maldonado-Ocampo (2001) | Fowler (1945) | IAvH-P 8735, 8763
*Hemigrammus hyanuary* Durbin, 1918				X	X				Castro (1987a) | Lasso et al. (2005) | ICN-MHN 2787
*Hemigrammus levis* Durbin, 1908				X	-				Correa (2003) | Maldonado-Ocampo et al. (2006a)
*Hemigrammus luelingi* Géry, 1964				X					Mojica et al. (2005)
*Hemigrammus lunatus* Durbin, 1918				X					Mojica et al. (2005)
*Hemigrammus melanochrous* Fowler, 1913				X					Correa (2003)
*Hemigrammus micropterus* Meek, 1907					X				Maldonado-Ocampo et al. (2006a)
*Hemigrammus microstomus* Durbin, 1918				X	X				Bogotá-Gregory and Maldonado-Ocampo (2006a) | Cala (1977) | IAvH-P 2605 | IICN-MHN 4273
*Hemigrammus newboldi* (Fernández-Yépez, 1949)				X	X				Maldonado-Ocampo (2001) | CZUT-IC 7204, 7250, 14763
*Hemigrammus ocellifer* (Steindachner 1882)				X					Mojica (1999) | CIACOL 2327, CZUT-IC 18072
*Hemigrammus pretoensis* Géry, 1965				X					Bogotá-Gregory and Maldonado-Ocampo (2006a) | USNM 216881 | ICN-MHN: 16850-16851
*Hemigrammus pulcher* Ladiges, 1938				X					Mojica et al. (2005)
*Hemigrammus rhodostomus* Ahl, 1924				X	X				Galvis et al. (2007b) | Cala (1977)
*Hemigrammus rubrostriatus* Zarske, 2015	X	X			X				Zarske (2015)
*Hemigrammus schmardae* (Steindachner, 1882)				X	X				Arbeláez et al. (2004) | Maldonado-Ocampo et al. (2006a)
*Hemigrammus stictus* (Durbin, 1909)				-	X				Cala (1977)
*Hemigrammus unilineatus* (Gill, 1858)				X	X				Galvis et al. (2007b) | Cala (1977)
*Hemigrammus vorderwinkleri* Géry, 1963				X	X				Correa (2003) | Maldonado-Ocampo et al. (2006a)
*Hemigrammus yinyang Lima & Sousa, 2009				X					CZUT-IC 4323, 4467, 4553, 4857, MPUJ 12525, 12623-12631
*Hyphessobrycon acaciae* García-Alzate, Román-Valencia & Prada-Pedreros, 2010	X	X			X				García-Alzate et al. (2010a) | MPUJ 5683, 393 | IUQ 2795, 2433, 2492, 2793
*Hyphessobrycon agulha* Fowler, 1913				X					Bogotá-Gregory and Maldonado-Ocampo (2006a) | IAvH-P 8331-8332, 8334, 8336-8344, 8346-8350, 8728, 8794, 8888, 8919, 8941, 8953, 8973, 8989, 9012, 9025, 9046, 9071, 9096, 9121, 9407, 11175-11178
*Hyphessobrycon amaronensis* García-Alzate, Román-Valencia & Taphorn, 2010	X	X		X					García-Alzate et al. (2010b) | IUQ 2286
*Hyphessobrycon bentosi* Durbin, 1908				X	-				Bogotá-Gregory and Maldonado-Ocampo (2005)
*Hyphessobrycon chocoensis* García-Alzate, Román-Valencia & Taphorn, 2013	X	X					X		García-Alzate et al. (2013) | IUQ 3035 | AUM 55075 | IUQ 2274, 2275, 3036, 3044
*Hyphessobrycon columbianus* Zarske & Géry, 2002	X	X						X	Zarske and Géry (2002) | MTDF 25497, 25498
*Hyphessobrycon condotensis* Regan, 1913	X	X					X		Regan (1913) | BMNH 1913.10.1.19-21
*Hyphessobrycon copelandi* Durbin, 1908				X					Mojica et al. (2005)
*Hyphessobrycon diancistrus* Weitzman, 1977					X				Weitzman (1977) | USNM 216607, 216606 | BMNH 1977.1.12.1-2 | MZUSP 13179-80
*Hyphessobrycon dorsalis Zarske, 2014					X				IAvH-P 1032, 2315
*Hyphessobrycon epicharis Weitzman & Palmer, 1997				X					CZUT-IC 4171, 5098
*Hyphessobrycon erythrostigma* (Fowler, 1943)				X					Weitzman and Palmer (1997)
*Hyphessobrycon gracilior* Géry, 1964				X					Bogotá-Gregory and Maldonado-Ocampo (2006a) | ICN-MHN 4277
*Hyphessobrycon heterorhabdus (Ulrey, 1894)				X					García-Alzate et al. (2008)
*Hyphessobrycon klausanni* García-Alzate, Urbano-Bonilla & Taphorn, 2017	X	X			X				García-Alzate et al. (2017)
*Hyphessobrycon loretoensis* Ladiges, 1938				X					ICN-MHN 16814, 16846-16848
*Hyphessobrycon mavro* García-Alzate, Román-Valencia & Prada-Pedreros, 2010	X	X			X				García-Alzate et al. (2010a) | IUQ 2791, 1964 | IMCN 3751
*Hyphessobrycon melazonatus* Durbin, 1908				X					Castro and Arboleda (1988) | ICN-MHN 16855-16856
*Hyphessobrycon metae* Eigenmann & Henn, 1914					X				Eigenmann and Henn (1914)
*Hyphessobrycon minimus* Durbin, 1909					X				Maldonado-Ocampo et al. (2008)
*Hyphessobrycon natagaima* García-Alzate, Taphorn, Román-Valencia & Villa-Navarro, 2015	X	X				X			García-Alzate et al. (2015b)
*Hyphessobrycon niger* García-Alzate, Román-Valencia & Prada-Pedreros, 2010	X	X			X				García-Alzate et al. (2010a) | MPUJ 5657, 5039 | IUQ 2794
*Hyphessobrycon ocasoensis* García-Alzate & Román-Valencia, 2008	X	X				X			García-Alzate and Román-Valencia (2008)
*Hyphessobrycon oritoensis* García-Alzate, Román-Valencia & Taphorn, 2008	X	X		X					García-Alzate et al. (2008) | IUQ 1574, 129, 1575 | MBUCV-V 33737 | MCGN 55844
*Hyphessobrycon otrynus Benine & Lopes, 2008					X				Ota et al. (2015)
*Hyphessobrycon peruvianus* Ladiges, 1938				X					Mojica et al. (2005)
*Hyphessobrycon poecilioides* Eigenmann, 1913	X	X	X			X	-		Eigenmann (1913) | CAS 77396 [ex IU 12850] | FMNH 56290 [ex CM 5091], 56291, 75150 | USNM 79214
*Hyphessobrycon proteus* Eigenmann, 1913	X	X				X		X	Eigenmann (1913) | García-Alzate et al. (2015b) | CAS 57603 [ex IU 12858], 60478-83 [ex IU 12852-57] | CIUA 295, 296 | FMNH 56293 [ex CM 5094], 56294-99, 56381-82, 69778 | SU 22754 | USNM 79215, 167819.
*Hyphessobrycon saizi* Géry, 1964	X	X			X				Géry (1964a) | USNM 198647 | ZMA 114206
*Hyphessobrycon sebastiani* García-Alzate, Román-Valencia & Taphorn, 2010	X	X					X		García-Alzate et al. (2010c)
*Hyphessobrycon sovichthys* Schultz, 1944								X	Ortega-Lara et al. (2012)
*Hyphessobrycon sweglesi* (Géry, 1961)	X	X		-	X				Géry (1961) | USNM 196090, IAvH-P 9517, 11276
*Hyphessobrycon taguae* García-Alzate, Román-Valencia & Taphorn, 2010	X	X		X	X				García-Alzate et al. (2010b) | García-Alzate et al. (2010a) | IUQ 2288
*Hyphessobrycon tukunai* Géry, 1965				X					Bogotá-Gregory and Maldonado-Ocampo (2006a) | ICN-MHN 4278
*Moenkhausia browni* Eigenmann, 1909				X	X				Galvis et al. (2007b) | Maldonado-Ocampo (2001)
*Moenkhausia ceros* Eigenmann, 1908					X				Maldonado-Ocampo et al. (2006a)
*Moenkhausia chrysargyrea* (Günther, 1864)				X	X				Maldonado-Ocampo (2001) | IAvH-P 8652, 8690, 8725, 8766, 8797, 8830, 8860, 8914
*Moenkhausia collettii* (Steindachner, 1882)				X	X				Correa (2003) | Cala (1977)
*Moenkhausia comma* Eigenmann, 1908				X					Mojica et al. (2005)
*Moenkhausia copei* (Steindachner, 1882)				X	X				Bogotá-Gregory and Maldonado-Ocampo (2006a) | Maldonado-Ocampo et al. (2004) | ICN-MHN 11028, 11130
*Moenkhausia cotinho* Eigenmann, 1908				X	X				Galvis et al. (2007b) | Maldonado-Ocampo and Bogotá-Gregory (2007)
*Moenkhausia dichroura* (Kner, 1858)				X	X				Mojica et al. (2005) | Maldonado-Ocampo et al. (2006a)
*Moenkhausia eigenmanni* Géry, 1964	X	X			X				Géry (1964a) | ANSP 112249, 139712 | ICN-MHN 17133 | MNHN 1980-1445 | USNM 198640
*Moenkhausia grandisquamis* (Müller & Troschel, 1845)				X	X				Mojica (1999) | Maldonado-Ocampo et al. (2006a) | ICN-MHN 3838, 6035, 6091
*Moenkhausia hysterosticta Lucinda, Malabarba & Benine, 2007					X				CZUT-IC 9821, MPUJ 4387, 4468
*Moenkhausia intermedia* Eigenmann, 1908				X	X				Galvis et al. (2007b) | Maldonado-Ocampo et al. (2006a)
*Moenkhausia jamesi* Eigenmann, 1908					X				Maldonado-Ocampo et al. (2006a)
*Moenkhausia justae Eigenmann, 1908					X				IAvH-P 847
*Moenkhausia latissima Eigenmann, 1908				X					IAvH-P 11014-11015
*Moenkhausia lepidura* (Kner, 1858)				X	X				Arbeláez et al. (2004) | Maldonado-Ocampo et al. (2006a)
*Moenkhausia megalops* (Eigenmann, 1907)				X	X				Arbeláez et al. (2004) | Maldonado-Ocampo et al. (2006a)
*Moenkhausia melogramma* Eigenmann, 1908				X	-				Arbeláez et al. (2004)
*Moenkhausia metae* Eigenmann, 1922	X	X			X				Eigenmann (1922) | CAS 55610 [ex IU 15026a], 55609 [ex IU 15026], 55608 [ex IU 13951] | FMNH 55214 | IAvH-P 9177
*Moenkhausia mikia Marinho & Langeani, 2010				X					CZUT-IC 3498, 3555, 3584, 4910, 4918, 5104, 8069
*Moenkhausia oligolepis* (Günther, 1864)				X	X				Fowler (1943) | Eigenmann (1922)
*Moenkhausia orteguasae* Fowler, 1943	X	X		X					Fowler (1943) | ANSP 70496
*Moenkhausia robertsi* Géry, 1964				X					Bogotá-Gregory and Maldonado-Ocampo (2006a) | ICN-MHN 5511, 6036
*Moenkhausia simulata* (Eigenmann, 1924)				X					Correa (2003)
*Paracheirodon axelrodi* (Schultz, 1956)					X				Cala (1977)
*Paracheirodon innesi* (Myers, 1936)				X					Mojica et al. (2005)
*Paracheirodon simulans* (Géry, 1963)					X				Weitzman and Fink (1983)
*Parapristella georgiae* Géry, 1964					X				Géry (1964a) | Lasso et al. 2004, Maldonado-Ocampo 2004, Maldonado-Ocampo et al. 2006b, Maldonado-Ocampo et al. 2008, Lasso et al. 2009
*Petitella georgiae* Géry & Boutière, 1964				X					Galvis et al. (2007b)
*Thayeria obliqua* Eigenmann, 1908				X	X				Maldonado-Ocampo (2001) | ICN-MHN 6025, 17140
*Tetragonopterus argenteus* Cuvier, 1816				X	X				Mojica et al. (2005) | Fowler (1945)
*Tetragonopterus chalceus* Spix & Agassiz, 1829				X	X				Fowler (1943) | Cala (1977)
*Aphyocharax alburnus* (Günther, 1869)				X	X				Fowler (1943) | Maldonado-Ocampo et al. (2006a)
*Aphyocharax colifax Taphorn & Thomerson, 1991				X					CZUT-IC 4271
*Aphyocharax erythrurus* Eigenmann, 1912				X	X				Galvis et al. (2007b) | ICN-MHN 5387
*Aphyocharax pusillus* Günther, 1868				X					Galvis et al. (2007b)
*Paragoniates alburnus* Steindachner, 1876				X	X				Mojica et al. (2005) | Galvis et al. (2007a)
*Phenagoniates macrolepis* (Meek & Hildebrand, 1913)								X	Eigenmann and Henn (1914) | Eigenmann (1922)
*Prionobrama filigera* (Cope, 1870)				X					Mojica et al. (2005)
*Xenagoniates bondi* Myers, 1942					X				Cala (1977)
*Atopomesus pachyodus* Myers, 1927				X					Myers (1927) | CAS 41736 [ex IU 17673]
*Axelrodia riesei* Géry, 1966	X	X			X				Géry (1966a) | USNM 207923, 207924 | ANSP 112512, 139715 | MNHN 1982-0538 | ZMA 113865
*Axelrodia stigmatias* (Fowler, 1913)				X					Arbeláez et al. (2004)
*Microschemobrycon callops* Böhlke, 1953					X				Maldonado-Ocampo et al. (2006a)
*Microschemobrycon casiquiare* Böhlke, 1953				X	X				Galvis et al. (2007b) | Maldonado-Ocampo et al. (2006a)
*Microschemobrycon geisleri* Géry, 1973				X					Arbeláez et al. (2004)
*Microschemobrycon melanotus (Eigenmann, 1912)					X				MPUJ 236, 247, 630, 1436, 4497
*Oxybrycon parvulus Géry, 1964				X					CZUT-IC 17910, 17953
*Parecbasis cyclolepis Eigenmann, 1914				X					CIACOL 272
*Tyttobrycon xeruini Géry, 1973					X				IAvH-P-14408, 15186, 15208, 15216, 15233, 15244, 15256, 15358, 15391, 15427, 15433, 15469, 15482, 15517, 15556, 15573, 15597, MPUJ 11025
*Grundulus bogotensis* (Humboldt, 1821)	X	X	X			X			Humboldt and Valenciennes (1821) | ZMB 33306, 3505, 31499
*Grundulus cochae* Román-Valencia, Paepke & Pantoja, 2003	X	X		X		-			Román-Valencia et al. (2001)| IUQ 453, 454, 455 | STRI 1369
*Nematobrycon lacortei* Weitzman & Fink, 1971	X	X					X	-	Weitzman and Fink (1971) | USNM 205594, 205595-96 | BMNH 1971.3.16.1 | CAS 13396 | FMNH 70525 | ZMA 110740
*Nematobrycon palmeri* Eigenmann, 1911	X	X					X	X	Eigenmann (1911) | Maldonado-Ocampo et al. (2013b)
*Cheirodontops geayi* Schultz, 1944					X				Urbano-Bonilla et al. (2009).
*Nanocheirodon insignis* (Steindachner, 1880)						X		X	Steindachner (1880) | Eigenmann (1915) | IAvH-P 11746 | MCNG 24875, 24981, 32355, 33285 | NMW 62543-44
*Odontostilbe fugitiva* Cope, 1870				X					Fowler (1945) | Lima RS (2003)
*Odontostilbe pao Bührnheim & Malabarba, 2007					X				Urbano-Bonilla et al. (2009).
*Odontostilbe pulchra* (Gill, 1858)					X				Bührnheim and Malabarba (2007)
*Odontostilbe splendida* Bührnheim & Malabarba, 2007					X				Bührnheim and Malabarba (2007) | ANSP 181040, 181041 | IAvH-P 3239, 3326, 3328, 3615, 7943, 7945, 7949, 7951, 8050, 8051, 8070, 9553-9555, 9557, 9558, 9697 | ICN-MHN 14168 | INPA 25173 | MBUCV 32890 | MCNG 54519 | MCP 38862
*Protocheirodon pi* (Vari, 1978)				X					CAS 41749
*Saccoderma hastata* (Eigenmann, 1913)	X	X				X		X	Eigenmann (1913) | Eigenmann (1915)
*Saccoderma melanostigma* Schultz, 1944								X	Ortega-Lara et al. (2012)
*Saccoderma robusta* Dahl, 1955	X	X						X	Dahl (1955) | MHNG 1066.39-42
*Eretmobrycon dahli* (Román-Valencia, 2000)							X		Román-Valencia (2000) | ICN-MNH 2722 | IAvH-P 4734 | IMCN 1689, 3894, 3905, 3911, 3918, 4052
*Eretmobrycon emperador* (Eigenmann & Ogle, 1907)						-	X	X	Maldonado-Ocampo et al. (2013b)
*Eretmobrycon guaytarae* (Eigenmann & Henn, 1914)	X	X				-	X		Eigenmann et al. (1914) | FMNH 56657 [ex CM 5474] | CAS 40844 [ex IU 13168]
*Eretmobrycon miraensis* (Fowler, 1945)	X	X				-	X		Fowler (1945) | ANSP 71686, 71687-92, 71693, 71694
*Eretmobrycon peruanus* (Müller & Troschel, 1845)							X	X	Maldonado-Ocampo et al. (2013b) | Fowler (1939)
*Eretmobrycon scleroparius* (Regan, 1908)							-	X	Maldonado-Ocampo et al. (2006b), Colombia. Biota Colombiana 7(1): 143-154
*Markiana geayi* (Pellegrin, 1909)				-	X				Lasso et al. (2005)
*Scopaeocharax atopodus (Böhlke, 1958)				X					CIACOL 2713
*Tyttocharax cochui* (Ladiges, 1950)				X					Arbeláez et al. (2004)
*Tyttocharax madeirae* Fowler, 1913				X					Román-Valencia et al. (2012)
*Tyttocharax metae* Román-Valencia, García-Alzate, Ruiz-C. & Taphorn B., 2012	X	X			X				Román-Valencia et al. (2012) | IUQ 2581
*Xenurobrycon heterodon* Weitzman & Fink, 1985				X					Bogotá-Gregory and Maldonado-Ocampo (2006a) | USNM 280246
*Chrysobrycon guahibo* Vanegas-Ríos, Urbano-Bonilla & Azpelicueta, 2015	X	X			X				Vanegas-Ríos et al. (2015)
*Chrysobrycon hesperus* (Böhlke, 1958)				X					Vanegas-Ríos et al. (2013b)
*Chrysobrycon mojicai* Vanegas-Ríos & Urbano-Bonilla, 2017	X	X		X					Vanegas-Ríos et al. (2017)
*Corynopoma riisei* Gill, 1858					X				Cala (1977)
*Gephyrocharax caucanus* Eigenmann, 1912	X	X				X	+		Eigenmann (1912) | CAS 44275-77 [ex IU 12668-70] | FMNH 56012 [ex CM 4802], 56013-15, 69553, 95008 | USNM 81921 |
*Gephyrocharax chocoensis* Eigenmann, 1912	X	X					X	X	Eigenmann (1912) | Vanegas-Ríos (2016)
*Gephyrocharax martae* Dahl, 1943	X	X				X			Dahl (1943)
*Gephyrocharax melanocheir* Eigenmann, 1912	X	X				X		X	Eigenmann (1912) | CAS 44292-93 [ex IU 12696-97] | FMNH 56049 [ex CM 4839], 56050-51, 69554 | USNM 79209 | Vanegas-Ríos (2016)
*Gephyrocharax sinuensis* Dahl, 1964	X	X						X	Dahl and Medem (1964) | ICN-MHN 749
*Gephyrocharax torresi* Vanegas-Ríos, Azpelicueta, Mirande & García Gonzales, 2013	X	X				X			Vanegas-Ríos et al. (2013a) | UIST 1767
*Gephyrocharax valencia* Eigenmann, 1920					X				Vanegas-Ríos (2016)
*Gephyrocharax venezuelae* Schultz, 1944								X	Ortega-Lara et al. (2012)
*Pterobrycon landoni* Eigenmann, 1913	X	X						X	Eigenmann (1913) | FMNH 56250 [ex CM 5051]
*Boehlkea fredcochui* Géry, 1966				X					Géry (1966b) | ANSP 111676 [ex Géry coll. 0124.18-19], 111668 | MNHN 1980-1425 | ZMA 113828
*Hemibrycon antioquiae* Román-Valencia, Ruiz-C, Taphorn, Mancera-Rodriguez & García-Alzate, 2013	X	X				X			Román-Valencia et al. (2013)
*Hemibrycon boquiae* (Eigenmann, 1913)	X	X				X			Eigenmann (1913) | CAS 44332 [ex IU 12831] | FMNH 56259 [ex CM 5059], 56260
*Hemibrycon brevispini* Román-Valencia & Arcila-Mesa, 2009	X	X				X			Román-Valencia and Arcila-Mesa (2009) | IUQ 542
*Hemibrycon cairoense* Román-Valencia & Arcila-Mesa, 2009	X	X				X			Román-Valencia and Arcila-Mesa (2009) | IUQ 534
*Hemibrycon cardalensis* Román-Valencia, Ruiz-C., Taphorn, Mancera-Rodriguez & García-Alzate, 2013	X	X				X			Román-Valencia et al. (2013)
*Hemibrycon carrilloi* Dahl, 1960	X	X				-	-	X	Dahl (1960c) | ICN-MHN 29, 31, 126
*Hemibrycon caucanus* (Eigenmann, 1913)						X		X	Eigenmann (1913) | Maldonado-Ocampo et al. (2006b)
*Hemibrycon colombianus* Eigenmann, 1914	X	X				X			Eigenmann et al. (1914) | MNH 56653 [ex CM 5470], 56654-56 | CAS 44350 [ex IU 13162], 44351 [ex IU 13163], 44352 [ex IU 13164], 44353 [ex IU 13165]
*Hemibrycon cristiani* (Román-Valencia, 1999)	X	X			X				Román-Valencia (1999) | IAvH-P 7529, 7530 | ICNMNH 3445, 3446-49 | IUQ 381-382
*Hemibrycon dariensis* Meek & Hildebrand, 1916								X	Román-Valencia and Ruiz-C. (2007)
*Hemibrycon decurrens* (Eigenmann, 1913)					-	X			FMNH 56255 [ex CM 5055] | CAS 39542 [IU 12829]
*Hemibrycon dentatus* (Eigenmann, 1913)	X	X				X			Eigenmann (1913)
*Hemibrycon fasciatus* Román-Valencia, Ruiz-C., Taphorn, Mancera-Rodriguez & García-Alzate, 2013	X	X				X			Román-Valencia et al. (2013)
*Hemibrycon galvisi* (Román-Valencia, 2000)	X	X		X					Román-Valencia (2000) | ICN-MHN 2720, 2721 | IUQ 221-223
*Hemibrycon jabonero* Schultz, 1944					-			X	Ortega-Lara et al. (2012)
*Hemibrycon jelskii* (Steindachner, 1877)				X					Maldonado-Ocampo et al. (2008)
*Hemibrycon loisae* (Géry, 1964)					X				Géry (1964a) | ANSP 139713 | IAvH-P 3639, 7528, 9194-9197, 9203, 9677-9680 | MNHN 1980-1426 | USNM 198645
*Hemibrycon metae* Myers, 1930				-	X				Myers (1930)
*Hemibrycon microformaa* Román-Valencia & Ruiz-C., 2007	X	X						X	Román-Valencia and Ruiz-C. (2007) | IUQ 510, 511-514 | MTDF 27628
*Hemibrycon paez* Román-Valencia & Arcila-Mesa, 2010	X	X				X			Román-Valencia and Arcila-Mesa (2010)
*Hemibrycon palomae* Román-Valencia, García-Alzate, Ruiz-C. & Taphorn, 2010	X	X				X			Román-Valencia et al. (2010a) | IUQ 2727-2729, 2802, 2846, 2300, 2301, AUM 50793
*Hemibrycon plutarcoi* (Román-Valencia, 2001)	X	X				X			Román-Valencia (2001) | ICN-MHN 4887 | IUQ 308, 461, 472, 473
*Hemibrycon quindos* Román-Valencia & Arcila-Mesa, 2010	X	X				X			Román-Valencia and Arcila-Mesa (2010)
*Hemibrycon rafaelense* Román-Valencia & Arcila-Mesa, 2008	X	X				X			Román-Valencia and Arcila-Mesa (2008) | ICN-MHN 6703, 3505 | IUQ 499, 509 | MCNG 54101 | MTD F 27623-24
*Hemibrycon raqueliae* Román-Valencia & Arcila-Mesa, 2010	X	X				X			Román-Valencia and Arcila-Mesa (2010)
*Hemibrycon sanjuanensis* Román-Valencia, Ruiz-C., Taphorn & García-Alzate, 2014	X	X					X		Román-Valencia et al. (2014b)
*Hemibrycon santamartae* Román-Valencia, Ruiz-C., García-Alzate & Taphorn, 2010	X	X						X	Román-Valencia et al. (2010d)
*Hemibrycon sierraensis* García-Alzate, Román-Valencia & Taphorn, 2015	X	X						X	García-Alzate et al. (2015a)
*Hemibrycon velox* Dahl, 1964	X	X				X		X	Dahl and Medem (1964) | ICN-MHN 1457, 1463, 1464, 2711
*Hemibrycon virolinica* Román-Valencia & Arcila-Mesa, 2010	X	X				X			Román-Valencia and Arcila-Mesa (2010)
*Hemibrycon yacopiae* Román-Valencia & Arcila-Mesa, 2010	X	X				X			Román-Valencia and Arcila-Mesa (2010)
*Carlastyanax aurocaudatus* (Eigenmann, 1913)	X	X	X			X			Eigenmann (1913) | CAS 68647 [ex IU 12911] | FMNH 56882 [ex CM 5162], 56883
*Creagrutus affinis* Steindachner, 1880						X	X	X	Steindachner (1880) | Eigenmann (1921)
*Creagrutus amoenus* Fowler, 1943				X					Fowler (1943) | ANSP 70499, 70500-02, 70503
*Creagrutus atratus* Vari & Harold, 2001	X	X			X	-			Vari and Harold (2001) | ANSP 134080 | IAvH-P 3659, 3660, 8426 | ICN-MHN 16981 | NRM 16842-43 | USNM 353866-67,
*Creagrutus barrigai Vari & Harold, 2001				X					CZUT-IC 12062
*Creagrutus bolivari* Schultz, 1944					X				Vari and Harold (2001)
*Creagrutus brevipinnis* Eigenmann, 1913	X	X				X			Eigenmann (1913) | CAS 41341-42 [ex IU 12728-29], 41375 [ex IU 12730] | FMNH 56095 [ex CM 4887a], 56096-98, 75172 | USNM 79188
*Creagrutus calai* Vari & Harold, 2001	X	X			X				Vari and Harold (2001) | ANSP 130527, 139149, 177718 | IAvH-P 7918 | NRM 16848-49, 43015-16 | USNM 353304, 353307, 353868
*Creagrutus caucanus* Eigenmann, 1913	X	X				X			Eigenmann (1913) | CAS 41373-74 [ex IU 12736-37], 69304 [ex IU 12738] | FMNH 56104 [ex CM 4895a], 56105-08
*Creagrutus cochui* Géry, 1964				X	+				Vari and Harold (2001)
*Creagrutus flavescens* Vari & Harold, 2001				X					Vari and Harold (2001)
*Creagrutus guanes* Torres-Mejia & Vari, 2005	X	X				X			Torres-Mejia and Vari (2005) | ICN-MHN 8520, 8521, 8522, 9893, 9894, 9895, 9896, 9897, 9898
*Creagrutus hildebrandi* Schultz, 1944								X	Ortega-Lara et al. (2012)
*Creagrutus leuciscus* Regan, 1913	X	X					X		Regan (1913)
*Creagrutus machadoi* Vari & Harold, 2001					X				Galvis et al. (2007a)
*Creagrutus maculosus* Román-Valencia, García-Alzate, Ruiz C. & Taphorn B, 2010	X	X			X				Román-Valencia et al. (2010b)
*Creagrutus magdalenae* Eigenmann, 1913	X	X				X			Eigenmann (1913) | CAS 41641 [ex IU 12722], 60056-57 [ex IU 12725-26] | FMNH 56088 [ex CM 4880], 56089, 56092-93, 69730
*Creagrutus maracaiboensis* Schultz, 1944								X	Ortega-Lara et al. (2012)
*Creagrutus maxillaris* (Myers, 1927)				X	X				Myers (1927) | IAvH-P 10192
*Creagrutus melasma* Vari, Harold & Taphorn, 1994					X				MPUJ 8814, 8815, 9207, 9208, 9209
*Creagrutus nigrostigmatus* Dahl, 1960	X	X				-		X	Dahl (1960b) | ICN-MHN 989 | SU 49491
*Creagrutus paralacus* Harold & Vari, 1994								X	Harold and Vari (1994)
*Creagrutus phasma* Myers, 1927					X				Vari and Harold (2001)
*Creagrutus runa Vari & Harold, 2001				X					CZUT-IC 4117, 4163
*Creagrutus taphorni* Vari & Harold, 2001					X				Urbano-Bonilla et al. (2009).
*Creagrutus tuyuka* Vari & Lima, 2003				X					Vari and Lima (2003)
*Creagrutus vexillapinnus* Vari & Harold, 2001					X				IAvH-P-14609, 14813
*Bryconacidnus hypopterus* (Fowler, 1943)	X	X		X					Fowler (1943) | ANSP 70505
*Ceratobranchia binghami Eigenmann 1927				X					ANSP 70504
*Ceratobranchia joanae Chernoff & Machado-Allison, 1990					X				CZUT-IC 12827
*Ceratobranchia obtusirustris* Eigenmann, 1914				X					Bogotá-Gregory and Maldonado-Ocampo (2006a) | IAvH-P 7526
*Knodus alpha* (Eigenmann, 1914)					X				Eigenmann et al. (1914) | CAS 18511 [ex IU 13157] | EBRG 295, 490, 493, 690, 822, 856, 886, 921, 922, 941, 1311 | FMNH 56646 [ex CM 5463], 56646-47 | IAvH-P 3558, 3640, 3641, 3643-3647, 3650-3653, 3657, 8042-8044, 8054, 8055, 9152-9158, 9178-9180, 9200-9202, 9664-9676, 9781, 11789
*Knodus breviceps* (Eigenmann, 1908)				X	X				IAvH-P 12289, 2538, 2575, 13165, 5176, 5187, 5418, 5419, 8315, 8316, 8317, 8318, 8319, 8320, 8321, 8322, 8323, 8324, 8325, 8326, 8327, 8328, 8329, 8330, 8655, 8694, 8729, 8764, 8795, 8828, 8858, 8889, 8920, 8942, 8954, 8974, 8990, 9013, 9026, 9047, 9072, 9097
*Knodus cinarucoense* Román-Valencia, Taphorn B. & Ruiz-C., 2008					X				IAvH-P-14385, 14396, 14443, 14610, 14637, 14705, 14806, 14811, 14994, 15059, 15228, 15237, 15542, 15583, 15603
*Knodus deuterodonoides* (Eigenmann, 1914)				-	X				Eigenmann et al. (1914) | FMNH 56644 [ex CM 5461], 56645 | CAS 61221 [ex IU 13156]
*Knodus gamma Géry, 1972				X					CZUT-IC 12073, 12967
*Knodus heteresthes* (Eigenmann, 1908)				X					Román-Valencia (2003b) | CZUT-IC 4151, 4200, 12308
*Knodus meridae* Eigenmann, 1911								X	Maldonado-Ocampo et al. (2008)
*Knodus moenkhausii* (Eigenmann & Kennedy, 1903)				X					Arbeláez et al. (2004) | Bogotá-Gregory and Maldonado-Ocampo (2006a)
*Knodus orteguasae* (Fowler, 1943)	X	X		X					Fowler (1943)
*Knodus tiquiensis* Ferreira & Lima, 2006				X					Ferreira and Lima (2006) | CZUT-IC 12307
*Rhinobrycon negrensis Myers, 1944					X				CZUT-IC 5081
*Argopleura chocoensis* (Eigenmann, 1913)	X	X					X	X	Eigenmann (1913) | Maldonado-Ocampo et al. (2006b)
*Argopleura conventus* (Eigenmann, 1913)	X	X				X			Eigenmann (1913) | CAS 39439 [ex IU 12802] | FMNH 56261 [ex CM 5060], 56262, 71290 | USNM 79174;
*Argopleura diquensis* (Eigenmann, 1913)	X	X				X			Eigenmann (1913) | CAS 39013 [ex IU 12820] | FMNH 56272 [ex CM 5072], 56273, 69690;
*Argopleura magdalenensis* (Eigenmann, 1913)	X	X				X			Eigenmann (1913) | BMNH 1924.3.3.37-38 | CAS 40827-32 [ex IU 12821-26] | FMNH 56263 [ex CM 5063], 56264-65, 56267-71, 56275, 69760 | USNM 79176, 236097
*Cyanogaster noctivaga Mattox, Britz, Toledo-Piza & Marinho, 2013				X	X				CZUT-IC 18065, MPUJ 273, 1257, 9846-9847, 10726-10729, 10744
*Othonocheirodus* Myers, 1927				+	X				CZUT-IC 5497, 12031, 12033, 12525, MPUJ 3747
*Bryconamericus andresoi* Román-Valencia, 2003	X	X				-	X		Román-Valencia (2003a) | IUQ 447, 448-449 | CAS 70099-101 | FMNH 56566-67
*Bryconamericus arilepis* Román-Valencia, Vanegas-Ríos & Ruiz-C, 2008	X	X				X			Román-Valencia et al. (2008) | IUQ 920, 1915, 1917, 1567
*Bryconamericus caldasi* Román-Valencia, Ruiz-C., Taphorn B. & García-Alzate, 2014	X	X				X			Román-Valencia et al. (2014a) | IUQ 3714
*Bryconamericus carlosi* Román-Valencia, 2003				X					Román-Valencia (2003c)
*Bryconamericus cismontanus* Eigenmann, 1914					X				Román-Valencia (2003c) | FMNH 56642 [ex CM 5459] | IAvH-P 3315, 3317-3319, 7920, 9168-9170, 9188-9193, 9480, 9481, 9650-9663, 9753
*Bryconamericus foncensis* Román-Valencia, Vanegas-Ríos & Ruiz-C, 2009	X	X				X			Román-Valencia et al. (2009) | IUQ 1941
*Bryconamericus guizae* Román-Valencia, 2003	X	X				-	X		Román-Valencia (2003a) | IUQ 450, 451-452 | STRI 1370
*Bryconamericus huilae* Román-Valencia, 2003	X	X				X			Román-Valencia (2003a) | IUQ 422, 312-313, 423-424, 462-463, 476-477) | STRI 00574
*Bryconamericus icelus* Dahl, 1964	X	X						X	Dahl and Medem (1964) | SMF 16284
*Bryconamericus ichoensis* Román-Valencia, 2000	X	X						X	Román-Valencia (2000) | ICN-MHN 2718, 2719
*Bryconamericus macarenae* Román-Valencia, García-Alzate, Ruiz-C. & Taphorn, 2010	X	X			X				Román-Valencia et al. (2010c) | IUQ 2448, 2271, 2326, 2435, 2437, 2440-2447, 2559-2561 | AUM 50297
*Bryconamericus macrophthalmus Román-Valencia, 2003				X					CZUT-IC 5124
*Bryconamericus multiradiatus* Dahl, 1960	X	X						X	Dahl (1960c) | ICN-MHN 82, 58
*Bryconamericus orinocoense Román-Valencia, 2003				X					CZUT-IC 4886, 4916
*Bryconamericus tolimae* Eigenmann, 1913	X	X				X			Eigenmann (1913)
*Microgenys minuta* Eigenmann, 1913	X	X	X			X			Eigenmann (1913) | CAS 47170 [ex IU 12818] | FMNH 56215 [ex CM 5007], 56216
*Astyanacinus yariguies* Torres-Mejia, Hernández & Senechal, 2012	X	X				X			Torres-Mejia et al. (2012) | UIST 1752
*Astyanax anterior Eigenmann, 1908				X					IAvH-P 8238-8240, 9117, 11012
*Astyanax atratoensis* Eigenmann, 1907	X	X						X	Eigenmann and Ogle (1907)
*Astyanax bimaculatus* (Linnaeus, 1758)				X	X	X	X	X	Fowler (1943) | Maldonado-Ocampo et al. (2013b) | Steindachner (1879a) | IAvH-P 1694, 9110
*Astyanax caucanus* (Steindachner, 1879)	X	X				X			Steindachner (1879c) | NMW 57372-76 | SMNS 2833 | ZMUC 993
*Astyanax daguae* Eigenmann, 1913	X	X	X				X	X	Eigenmann (1913) | Maldonado-Ocampo et al. (2006b)
*Astyanax fasciatus* (Cuvier, 1819)				X	X	X	X	X	Eigenmann (1922) | Fowler (1943) | Lima et al. (2003) | Eigenmann (1921)
*Astyanax fasslii* (Steindachner, 1915)	X	X				X			Steindachner (1915)
*Astyanax filiferus* (Eigenmann, 1913)	X	X				X		X	Eigenmann (1913) | Maldonado-Ocampo et al. (2006b)
*Astyanax gisleni* Dahl, 1943	X	X				X			Dahl (1943)
*Astyanax guianensis Eigenmann, 1909				X					Marinho (2015)
*Astyanax integer* Myers, 1930				-	X				Myers (1930) | IAvH-P 3300, 3305-3307, 3309, 3497-3499, 3503, 3505-3507, 3512, 3514-3516, 9698-9704 | SU 23726
*Astyanax magdalenae* Eigenmann & Henn, 1916						X		X	Eigenmann and Henn (1916) | Maldonado-Ocampo et al. (2006b)
*Astyanax maximus* (Steindachner, 1876)				X	-				Bogotá-Gregory and Maldonado-Ocampo (2006a) | IAvH-P 1731
*Astyanax megaspilura* Fowler, 1944	X	X					X	X	Fowler (1944) | Maldonado-Ocampo et al. (2006b)
*Astyanax metae* Eigenmann, 1914					X				Eigenmann et al. (1914) | CAS 39229 [ex IU 13153] | CRPUT 302, 305, 327, 331, 332 | FMNH 56640 [ex CM 5457], 56641 | IAvH-P 3302, 3521, 3522, 3525-3531, 3535-3544, 7909, 9484, 9643-964
*Astyanax microlepis* Eigenmann, 1913	X	X		-	-	X	-		Eigenmann (1913) | CAS 39341-45 [ex IU 12769-73] | FMNH 56209 [ex CM 5001], 56210-14, 69559-61, 71291, 95006 | USNM 79167
*Astyanax orthodus* Eigenmann, 1907	X	X					X	X	Eigenmann and Ogle (1907) | Maldonado-Ocampo et al. (2006b)
*Astyanax ruberrimus* Eigenmann, 1913						-	X	X	Eigenmann (1913) | Maldonado-Ocampo et al. (2006b) | Eigenmann (1921)
*Astyanax scintillans* Myers, 1928					X				IAvH-P 4979
*Astyanax siapae* Garutti, 2003					X				IMCN 3652
*Astyanax stilbe* (Cope, 1870)								X	Eigenmann (1921)
*Astyanax venezuelae* Schultz, 1944					X				Urbano-Bonilla et al. (2009).
*Brittanichthys axelrodi Géry, 1965					X				Weitzman et al. (2005)
*Bryconella pallidifrons* (Fowler 1946)				X					F. C. T. Lima (pers. com.): as *Hemigrammus analis* in Galvis et al. (2006) | CZUT-IC 17948, IAvH-P 2605, MPUJ 12206, 12207
*Ctenobrycon oliverai Benine, Lopes & Ron, 2010					X				CZUT-IC 7217, 9056, 9279, 9321, 9670, 9752, 9785, 9801, 9810, 9851
*Ctenobrycon spilurus* (Valenciennes, 1850)				X	X				Mojica et al. (2005) | Galvis et al. (2007a)
*Genycharax tarpon* Eigenmann, 1912	X	X	X			X			Eigenmann (1912) | CAS 44271-73 [ex IU 12672-74] | FMNH 56018 [ex CM 4808], 56019-2, 69547, 69779 | USNM 79207
*Jupiaba abramoides* (Eigenmann, 1909)					X				Cala (1977) | ICN-MHN 546
*Jupiaba anteroides* (Géry, 1965)				X	X				Arbeláez et al. (2004) | Galvis et al. (2007a)
*Jupiaba asymmetrica* (Eigenmann, 1908)				X					Fowler (1945) | Lima et al. (2003)
*Jupiaba polylepis* (Günther, 1864)					X				IAvH-P 2013
*Jupiaba scologaster* (Weitzman & Vari, 1986)				X					Galvis et al. (2007b)
*Jupiaba zonata* (Eigenmann, 1908)				X					Galvis et al. (2007b)
*Pseudochalceus kyburzi* Schultz, 1966	X	X					X		Schultz (1966) | USNM 231738 [was USNM 257403-F27], 171751, 231739 [ex USNM 258173-F1]
*Pseudochalceus longianalis* Géry, 1972							X		Géry (1972) | MHNG 1226.90., 1226.91-99 | ANSP 140067 | Géry personal collection 0690
*Schultzites axelrodi* Géry, 1964	X	X			X				Géry (1964a) | USNM 198642
*Serrabrycon magoi* Vari, 1986					X				FMNH 92642
*Thrissobrycon pectinifer* Böhlke, 1953					X				Maldonado-Ocampo et al. (2006a)
**Gasteropelecidae9 spp**									
*Carnegiella marthae* Myers, 1927				X	X				Mojica et al. (2005) | Cala (1977)
*Carnegiella myersi* Fernández-Yépez, 1950				X					Mojica (1999) | NRM 200 | IMCN 7062
*Carnegiella schereri* Fernández-Yépez, 1950				X					Galvis et al. (2007b)
*Carnegiella strigata* (Günther, 1864)				X	X				Weitzman (1960) | Cala (1977)
*Engraulisoma taeniatum* Castro, 1981				-	X				IAvH-P 13957
*Gasteropelecus maculatus* Steindachner, 1879						X	X	X	Eigenmann (1912) | Eigenmann (1922) | Weitzman (1960)
*Gasteropelecus sternicla* (Linnaeus, 1758)				X					Weitzman (1960)
*Thoracocharax securis* (De Filippi, 1853)				X					Mojica et al. (2005)
*Thoracocharax stellatus* (Kner, 1858)				X	X				Weitzman (1960)
**Bryconidae22 spp (2 added and 3 deleted from 2008)**									
*Brycon amazonicus* (Agassiz, 1829)				+	X				Maldonado-Ocampo et al. (2006a) | IAvH-P 11603
*Brycon argenteus* Meek & Hildebrand, 1913							X		Maldonado-Ocampo et al. (2013b)
*Brycon dentex* Günther, 1860							X		Mojica et al. (2004) | ICN-MHN 2351, 5025
*Brycon falcatus* Müller & Troschel, 1844				X	X				Bogotá-Gregory and Maldonado-Ocampo (2006a) | Maldonado-Ocampo et al. (2006a) | IAvH-P 5326 | ICN-MHN 4260, 2000, 5677
*Brycon fowleri* Dahl, 1955	X	X				X		X	Dahl (1955) | ICN-MHN 2044
*Brycon henni* Eigenmann, 1913	X	X				X	X		Eigenmann (1913) | Maldonado-Ocampo et al. (2005)
*Brycon hilarii (Valenciennes, 1850)				X					Lima (2017)
*Brycon labiatus* Steindachner, 1879	X	X	X			X			Steindachner (1879b) | NMW
*Brycon medemi* Dahl, 1960	X	X						X	Dahl (1960c) | ICN-MHN 41, 73
*Brycon meeki* Eigenmann & Hildebrand, 1918	X	X					X	X	Eigenmann (1918a) | Maldonado-Ocampo et al. (2006b)
*Brycon melanopterus* (Cope, 1871)				X	-				Mojica et al. (2005) | Galvis et al. (2007a)
*Brycon moorei* Steindachner, 1878	X	X	X			X		X	Steindachner (1878) | Mojica et al. (2006b)
*Brycon oligolepis* Regan, 1913							X	X	Regan (1913) | Maldonado-Ocampo et al. (2006b) | Eigenmann (1922)
*Brycon pesu* Müller & Troschel, 1845				X	X				Galvis et al. (2007b) | Galvis et al. (2007a)
*Brycon polylepis Moscó Morales 1988					X				Lima (2017) | IAvH-P 3554, 3671
*Brycon posadae* Fowler, 1945							X		Fowler (1945) | ANSP 71695
*Brycon rubricauda* Steindachner, 1879	X	X	X			X		-	Steindachner (1879b)
Brycon sinuensis Dahl, 1955	X	X	X					X	Dahl (1955)
*Brycon striatulus* (Kner, 1863)							X	X	Regan (1913) | Maldonado-Ocampo et al. (2008) |
*Brycon whitei* Myers & Weitzman, 1960	X	X			X				Myers and Weitzman (1960) | Urbano-Bonilla et al. (2009).
*Salminus affinis* Steindachner, 1880			X	-		X		X	Steindachner (1880) | ANSP 140067 | MHNG 1226.90, 1226.91-99
*Salminus hilarii* Valenciennes, 1850				X	X				Fowler (1943) | Cala (1977)
**Triportheidae12 spp (2 added and 2 deleted from 2008)**									
*Agoniates anchovia* Eigenmann, 1914				X					Mojica et al. (2005)
*Agoniates halecinus* Müller & Troschel, 1845				X	X				Bogotá-Gregory and Maldonado-Ocampo (2005) | Maldonado-Ocampo et al. (2006a)
*Triportheus albus* Cope, 1872				X					Mojica et al. (2005)
*Triportheus angulatus* (Spix & Agassiz, 1829)				X					Mojica et al. (2005)
*Triportheus auritus* (Valenciennes, 1850)				+	X				Maldonado-Ocampo and Bogotá-Gregory (2007) | Galvis et al. (2007b)
*Triportheus brachipomus* (Valenciennes, 1850)					X				Galvis et al. (2007a)
*Triportheus culter (Cope, 1872)				X					CZUT-IC 7310
*Triportheus magdalenae* (Steindachner, 1878)	X	X				X			Steindachner (1878) | NMW 69151-54 | ZMUC 87
*Triportheus orinocensis* Malabarba, 2004					X				Maldonado-Ocampo et al. (2013a)
*Triportheus pictus* (Garman, 1890)				X					Correa (2003)
*Triportheus rotundatus (Jardine, 1841)				X					IAvH-P 11059
*Triportheus venezuelensis* Malabarba, 2004					X				Galvis et al. (2007a)
**Iguanodectidae12 spp (1 added and 1 deleted from 2008)**									
*Bryconops affinis* (Günther, 1864)				X	X				Bogotá-Gregory and Maldonado-Ocampo (2005) | Cala (1977) | IAvH-P 2725, 3579
*Bryconops alburnoides* Kner, 1858				X	X				Galvis et al. (2007b) | ICN-MHN 9918
*Bryconops caudomaculatus* (Günther, 1864)				X	X				Correa (2003) | Eigenmann (1922)
*Bryconops collettei* Chernoff & Machado-Allison, 2005				X					Maldonado-Ocampo et al. (2008)
*Bryconops humeralis* Machado-Allison, Chernoff & Buckup, 1996					X				Maldonado-Ocampo et al. (2006a)
*Bryconops giacopinii* (Fernández-Yépez, 1950)				X	X				Bogotá-Gregory and Maldonado-Ocampo (2006a) | Maldonado-Ocampo and Bogotá-Gregory (2007) | IAvH-P 1691, 5307 | ICN-MHN 4625, 10371
*Bryconops inpai* Knöppel, Junk & Géry, 1968				X	X				Mojica et al. (2005) | Maldonado-Ocampo et al. (2006a)
*Bryconops magoi Chernoff & Machado-Allison, 2005				X					CZUT-IC 4129
*Iguanodectes adujai* Géry, 1970				X	X				Maldonado-Ocampo et al. (2008)
*Iguanodectes geisleri* Géry, 1970				X	X				Castro and Arboleda (1988) | Maldonado-Ocampo (2001)
*Iguanodectes purusii* (Steindachner, 1908)				X					Mojica et al. (2005)
*Iguanodectes spilurus* (Günther, 1864)				X	X				Arbeláez et al. (2004) | Maldonado-Ocampo et al. (2006a)
**Chalceidae3 spp**									
*Chalceus epakros* Zanata & Toledo-Piza, 2004				X	X				Zanata and Toledo-Piza (2004) | Maldonado-Ocampo et al. (2006a)
*Chalceus erythrurus* (Cope, 1870)				X					Mojica et al. (2005)
*Chalceus macrolepidotus* Cuvier, 1816				X	X				Correa (2003) | Cala (1977)
**Gymnotiformes78 spp (12 added and 8 deleted from 2008)**									
**Gymnotidae12 spp (2 deleted from 2008)**									
*Electrophorus electricus* (Linnaeus, 1766)				X	X				Maldonado-Ocampo and Albert. (2003) | Cala (1977)
*Gymnotus anguillaris* Hoedeman, 1962					X				Maldonado-Ocampo and Albert. (2003)
*Gymnotus ardilai* Maldonado-Ocampo & Albert, 2004	X	X	X			X			Maldonado-Ocampo and Albert (2004) | IAvH-P 3477, 4001
*Gymnotus carapo* Linnaeus, 1758				X	X				Fowler (1945) | Eigenmann (1922)
*Gymnotus cataniapo* Mago-Leccia, 1994					X				Maldonado-Ocampo and Albert (2003)
*Gymnotus choco* Albert, Crampton & Maldonado-Ocampo, 2003	X	X	X				X	X	Albert and Crampton (2003b) | ICN-MHN 6621 | NRM 27734
*Gymnotus coropinae* Hoedeman, 1962				X	X				Maldonado-Ocampo and Albert (2003) | Galvis et al. (2007a)
*Gymnotus henni* Albert, Crampton & Maldonado-Ocampo, 2003			X				X		Albert and Crampton (2003b) | CAS 47290, 217162 | FMNH 56793
*Gymnotus javari* Albert, Crampton & Hagedorn, 2003				X					Galvis et al. (2007b)
*Gymnotus pedanopterus* Mago-Leccia, 1994				X					Maldonado-Ocampo and Albert (2003)
*Gymnotus stenoleucus* Mago-Leccia, 1994				X	X				Maldonado-Ocampo and Albert (2003)
*Gymnotus tigre* Albert & Crampton, 2003				X					Albert and Crampton (2003b) | UF 25552, 128412 | ICN-MHN 6690
**Hypopomidae9 spp (5 added and 2 deleted from 2008)**									
*Brachyhypopomus batesi* Crampton, de Santana, Waddell & Lovejoy, 2016				X					Crampton et al. (2016)
*Brachyhypopomus beebei* (Schultz, 1944)				X	X				Maldonado-Ocampo and Albert (2003)
*Brachyhypopomus bennetti* Sullivan, Zuanon & Cox Fernandes, 2013				X					Sullivan et al. (2013b)
*Brachyhypopomus brevirostris* (Steindachner, 1868)				X	X	-			Maldonado-Ocampo and Albert (2003)
*Brachyhypopomus bullocki* Sullivan & Hopkins, 2009				X	X				Sullivan and Hopkins (2009) | Crampton et al. (2016) | ANSP 187477
*Brachyhypopomus flavipomus* Crampton, de Santana, Waddell & Lovejoy, 2016				X					Crampton et al. (2016)
*Brachyhypopomus occidentalis* (Regan, 1914)						X	X	X	Regan (1914) | Maldonado-Ocampo et al. (2006b) | Albert and Crampton (2003a)
*Brachyhypopomus sullivani* Crampton, de Santana, Waddell & Lovejoy, 2016					X				Crampton et al. (2016)
*Microsternarchus bilineatus* Fernández-Yépez, 1968					X				Maldonado-Ocampo and Albert (2003)
**Rhamphichthyidae10 spp (1 added and 1 deleted from 2008)**									
*Gymnorhamphichthys hypostomus* Ellis, 1912				X	X				Maldonado-Ocampo and Albert (2003)
*Gymnorhamphichthys rondoni* (Miranda Ribeiro, 1920)				X	X				Maldonado-Ocampo and Albert (2003)
*Rhamphichthys apurensis (Fernández-Yépez, 1968)					X				CZUT-IC 3244, 5178, 5179, 9362, 9421, 9422, 9423, 9424, 9448, 9449, 9547
*Rhamphichthys drepanium* Triques, 1999					X				Maldonado-Ocampo and Albert (2003)
*Rhamphichthys marmoratus* Castelnau, 1855					X				Cala (1977)
*Rhamphichthys rostratus* (Linnaeus, 1766)				X	X				Maldonado-Ocampo and Albert (2003)
*Hypopygus lepturus* Hoedeman, 1962				X	X				Maldonado-Ocampo and Albert (2003)
*Hypopygus neblinae* Mago-Leccia, 1994				X	X				Galvis et al. (2007b) | Maldonado-Ocampo and Albert (2003)
*Steatogenys duidae* (La Monte, 1929)				X	X				Maldonado-Ocampo et al. (2006a) | Maldonado-Ocampo and Albert (2003)
*Steatogenys elegans* (Steindachner, 1880)				X	X				Maldonado-Ocampo and Albert (2003)
**Sternopygidae16 spp (2 added from 2008)**									
*Distocyclus conirostris* (Eigenmann & Allen, 1942)				X					Maldonado-Ocampo and Albert (2003)
‘*Eigenmannia’ goajira* Schultz, 1949								X	Maldonado-Ocampo and Albert (2003)
*Eigenmannia humboldtii* (Steindachner, 1878)						X		X	Steindachner (1878) | Maldonado-Ocampo et al. (2006b)
*Eigenmannia limbata* (Schreiner & Ribeiro, 1903)				X					Maldonado-Ocampo and Albert (2003)
*Eigenmannia macrops* (Boulenger, 1897)					X				Maldonado-Ocampo and Albert (2003)
*Eigenmannia nigra* Mago-Leccia, 1994				X					Maldonado-Ocampo and Albert (2003)
*Eigenmannia virescens* (Valenciennes, 1836)				X	X	X	X	X	Eigenmann and Ward (1905) | Maldonado-Ocampo and Albert (2003)
*Rhabdolichops caviceps* (Fernández-Yépez, 1968)					X				Maldonado-Ocampo and Albert (2003)
*Rhabdolichops eastwardi Lundberg & Mago-Leccia, 1986				X					CZUT-IC 3642, 3987
*Rhabdolichops troscheli* (Kaup, 1856)				X					Maldonado-Ocampo and Albert (2003)
*Rhabdolichops zareti Lundberg & Mago-Leccia, 1986					X				CZUT-IC 9471
*Sternopygus aequilabiatus* (Humboldt, 1805)						X	+	+	Humboldt (1805) | Maldonado-Ocampo et al. (2013b)
*Sternopygus astrabes* Mago-Leccia, 1994					X				Maldonado-Ocampo and Albert (2003)
*Sternopygus dariensis* Meek & Hildebrand, 1916							X	X	Hulen et al. (2005)
*Sternopygus macrurus* (Bloch & Schneider, 1801)				X	X				Maldonado-Ocampo and Albert (2003) | Eigenmann (1922)
*Sternopygus pejeraton* Schultz, 1949								X	Ortega-Lara et al. (2012)
**Apteronotidae31 spp (4 added and 3 deleted from 2008)**									
*Adontosternarchus balaenops* (Cope, 1878)				X					Maldonado-Ocampo and Albert (2003)
*Adontosternarchus clarkae* Mago-Leccia, Lundberg & Baskin, 1985				X					Mago-Leccia et al. (1985)
*Adontosternarchus devenanzii* Mago-Leccia, Lundberg & Baskin, 1985					X				Mago-Leccia et al. (1985)
*Adontosternarchus sachsi (Peters, 1877)					X				CZUT-IC 9438, 9451, 9453, 9455, 9457, 9475, 9490, 9492
*Apteronotus albifrons* (Linnaeus, 1766)					X				Maldonado-Ocampo and Albert (2003)
*Apteronotus anu* de Santana & Vari, 2013								X	de Santana and Vari (2013)
*Apteronotus apurensis* Fernández-Yépez, 1968					X				Maldonado-Ocampo and Albert (2003)
*Apteronotus bonapartii* (Castelnau, 1855)				X					Maldonado-Ocampo and Albert (2003)
*Apteronotus cuchillejo* (Schultz, 1949)								X	Maldonado-Ocampo and Albert (2003)
*Apteronotus cuchillo* Schultz, 1949								X	Maldonado-Ocampo and Albert (2003)
*Apteronotus eschmeyeri* de Santana, Maldonado-Ocampo, Severi & Mendez, 2004	X	X				X		X	de Santana et al. (2004) | Maldonado-Ocampo et al. (2006b)
*Apteronotus galvisi* de Santana, Maldonado-Ocampo & Crampton, 2007	X	X			X				de Santana et al. (2007)
*Apteronotus jurubidae* (Fowler, 1944)	X	X					X		Fowler (1944) | ANSP 71435
*Apteronotus macrolepis* (Steindachner, 1881)				X					Bogotá-Gregory and Maldonado-Ocampo 2006
*Apteronotus macrostomus* (Fowler, 1943)	X	X			X				Fowler (1943) | ANSP 70528
*Apteronotus magdalenensis* (Miles, 1945)	X	X	X			X			Miles (1945) | BMNH 1947.7.1.138 | USNM 123795
*Apteronotus mariae* (Eigenmann & Fisher, 1914)	X	X				X			Eigenmann and Fisher (1914) | FMNH 56774 [ex CM 5594] | CAS 62345 [ex IU 13375]
*Apteronotus milesi* de Santana & Maldonado-Ocampo, 2005	X	X				X			de Santana and Maldonado-Ocampo. (2005) | IAvH-P 3996 | CAS 72249 [ex IU 13379], 72250 [ex IU 13379] | IAvH-P 3997 | ICMN 2500 | ICNMNH 3155 | FMNH 56775, 56776, 56777, 56778
*Apteronotus rostratus* (Meek & Hildebrand, 1913)						+	X	X	de Santana and Vari (2013) | Eigenmann and Fisher (1914)
*Apteronotus spurrellii* (Regan, 1914)	X	X					X		Regan (1914) | BMNH 1914.5.18.90-93
*Parapteronotus hasemani* (Ellis, 1913)				X					Maldonado-Ocampo and Albert (2003)
*Platyurosternarchus macrostomus* (Günther, 1870)				X	X				Maldonado-Ocampo and Albert (2003)
*Sternarchorhynchus mormyrus* (Steindachner, 1868)				X	X				Maldonado-Ocampo and Albert (2003)
*Sternarchorhynchus oxyrhynchus* (Müller & Troschel, 1848)				-	+				Maldonado-Ocampo and Albert (2003)
*Sternarchorhynchus roseni* Mago-Leccia, 1994					X				Maldonado-Ocampo and Albert (2003) | IAvH-P 2981
*Compsaraia compsus* (Mago-Leccia, 1994)					X				Maldonado-Ocampo and Albert (2003)
*Sternarchogiton nattereri* (Steindachner, 1868)				X					Maldonado-Ocampo and Albert (2003)
*Sternarchella orinoco Mago-Leccia, 1994					X				CZUT-IC 9493, 9535
*Sternarchella orthos Mago-Leccia, 1994					X				CZUT-IC 9445, 9482, 9484
*Sternarchella schotti* (Steindachner, 1868)				X	+				Maldonado-Ocampo and Albert (2003) | Evans et al. (2017)
*Sternarchorhamphus muelleri* (Steindachner, 1881)				X					Maldonado-Ocampo and Albert (2003)
**Siluriformes588 spp (98 added and 61 deleted from 2008)**									
**Trichomycteridae66 spp (16 added and 6 deleted from 2008)**									
*Eremophilus mutisii* Humboldt, 1805	X	X	X			X			Humboldt (1805)
*Ituglanis guayaberensis* (Dahl, 1960)	X	X			X				Dahl (1960a)
*Ituglanis metae* (Eigenmann, 1917)					X				Eigenmann (1917a) | CAS 58138 [ex IU 13770]
*Rhizosomichthys totae* (Miles, 1942)	X	X	X		+	-			Miles (1942) | ICN-MHN 353 | MCZ 35744 | SU 37074
*Trichomycterus arhuaco* Ardila Rodríguez, 2016	X	X				X			Ardila Rodríguez (2016c) | Holotype CAR 684
*Trichomycterus ballesterosi* Ardila Rodríguez, 2011	X	X						X	Ardila Rodríguez (2011a) | CAR 400
*Trichomycterus banneaui* (Eigenmann, 1912)	X	X				X			Eigenmann (1912) | CAS 58127 [ex IU 12677] | Holotype FMNH 56025 [ex CM 4815], 56026, 69815 | USNM 79234
*Trichomycterus bogotensis* (Eigenmann, 1912)	X	X				X			Eigenmann (1912) | CAS 58118 [ex IU 12679] | Holotype FMNH 56030 [ex CM 4820] | 56031, 56044 | AMNH 7107 | USNM 79232
*Trichomycterus cachiraensis* Ardila Rodríguez, 2008	X	X	X			X			Ardila Rodríguez (2008c) | CAR 125, 97 | CZUT-IC2920 | IAvH-P 11114 | MBUCV-V35384;
*Trichomycterus caliensis* (Eigenmann, 1912)	X	X	X			X	-		Eigenmann (1912) | FMNH 56029 [ex CM 4819 not 6819]
*Trichomycterus casitaensis* Ardila Rodríguez, 2017	X	X						X	Ardila Rodríguez (2017b)
*Trichomycterus chapmani* (Eigenmann, 1912)	X	X				X	-		Eigenmann (1912) | CAS 58128 [ex IU 12678] | Holotype FMNH 56027 [ex CM 4817], 50628, 69813 | USNM 79233
*Trichomycterus dorsostriatum* (Eigenmann, 1917)	X	X			X				Eigenmann (1917a) | CAS 64579 [ex IU 13810] | Holotype FMNH 58096 [ex CM 7093], 58097
*Trichomycterus emanueli (Schultz, 1944)								X	IAvH-P 9797
*Trichomycterus garciamarquezi* Ardila Rodríguez, 2016	X	X						X	Ardila Rodríguez (2016c) | Holotype CAR 682
*Trichomycterus gorgona* Fernández & Schaefer, 2005	X	X	X				X		Fernández and Schaefer (2005) | ANSP 149946 | ICN-MHN 10019
*Trichomycterus kankuamo* Ardila Rodríguez, 2016	X	X				X			Ardila Rodríguez (2016c) | Holotype CAR 685
*Trichomycterus knerii* Steindachner, 1882					X				Eigenmann (1918b)
*Trichomycterus latidens* (Eigenmann, 1917)	X	X				-	X		Eigenmann (1917a) | CAS 76335 [ex IU 13801]
*Trichomycterus latistriatus* (Eigenmann, 1917)	X	X				X			Eigenmann (1917a) | FMNH 58449 [ex CM 7450]
*Trichomycterus maldonadoi* Ardila Rodríguez, 2011	X	X						X	Ardila Rodríguez (2011b) | CAR 380, 500
*Trichomycterus manaurensis* Ardila Rodríguez, 2016	X	X				X			Ardila Rodríguez (2016c) | Holotype CAR 683
*Trichomycterus maracaiboensis* (Schultz, 1944)								X	Ortega-Lara et al. (2012)
*Trichomycterus migrans* (Dahl, 1960)	X	X			X				Dahl (1960a) | ICN-MHN 399, 400
*Trichomycterus montesi* Ardila Rodríguez, 2016	X	X				X			Ardila Rodríguez (2016c) | Holotype CAR 681
*Trichomycterus nietoi* Ardila Rodríguez, 2014	X	X						X	Ardila Rodríguez (2014)
*Trichomycterus nigromaculatus* Boulenger, 1887	X	X				-	-	X	Boulenger (1887) | BMNH 1880.2.26.16-17
*Trichomycterus ocanaensis* Ardila Rodríguez, 2011	X	X						X	Ardila Rodríguez (2011d) | IAvH-P 11115, 9797
*Trichomycterus regani* (Eigenmann, 1917)	X	X					X		Eigenmann (1917a) | CAS 64591 [ex IU 13772]
*Trichomycterus retropinnis* Regan, 1903	X	X				X			Regan (1903) | BMNH 1899.8.21.12-13
*Trichomycterus romeroi* (Fowler, 1941)	X	X				X			Fowler (1941) | ANSP 69331, 69332-35
*Trichomycterus ruitoquensis* Ardila-Rodríguez, 2007	X	X				X			Ardila Rodríguez (2007) | CAR 340, 37, 88, 89, 325, 331, 332 | IAvH-P 4342, 4344 | IMCN 4195
*Trichomycterus sandovali* Ardila Rodríguez, 2006	X	X	X			X			Ardila Rodríguez (2006a) | CAR 116
*Trichomycterus santanderensis* Castellanos-Morales, 2007	X	X				X			Castellanos-Morales (2007) | CAC-CDMB 035
*Trichomycterus sketi* Castellanos-Morales, 2011	X	X				X			Castellanos-Morales (2011) | CAC-CDMB 104
*Trichomycterus spilosoma* (Regan, 1913)	X	X				-	X		Regan (1913) | BMNH 1910.7.11.106-107, 1910.7.11.108
*Trichomycterus steindachneri* DoNascimiento, Prada-Pedreros & Guerrero-Kommritz, 2014	X	X			X				DoNascimiento et al. (2014)
*Trichomycterus stellatus* (Eigenmann, 1918)	X	X				X			Eigenmann (1918b) | CAS 58121 [ex IU 13814] | FMNH 58101 [ex CM 7097], 58102
*Trichomycterus straminius* (Eigenmann, 1917)	X	X				X		-	Eigenmann (1917a) | CAS 58148 [ex IU 13818], 58105 [ex IU 13804] | FMNH 58105 [ex CM 7101], 58091-92
*Trichomycterus striatus* (Meek & Hildebrand, 1913)						-	-	+	Eigenmann (1918b)
*Trichomycterus taenia* Kner, 1863						-	X		Regan (1913)
*Trichomycterus tetuanensis* García-Melo, Villa-Navarro & DoNascimiento, 2016	X	X				X			García-Melo et al. (2016)
*Trichomycterus torcoromaensis* Ardila-Rodríguez, 2016	X	X				X			Ardila Rodríguez (2016a)
*Trichomycterus transandianus* (Steindachner, 1915)	X	X				X			Steindachner (1915) | NMW 44475
*Trichomycterus uisae* Castellanos-Morales, 2008	X	X				X			Castellanos-Morales (2008) | CAC-CDMB 072
*Trichomycterus unicolor* (Regan, 1913)	X	X					X		Regan (1913) | BMNH 1913.10.1.42-43
*Malacoglanis gelatinosus* Myers & Weitzman, 1966	X	X		X	+				Myers and Weitzman (1966) | CZUT-IC 13816, IAvH-P 13640, SU 50754, 50755
*Tridens* Eigenmann & Eigenmann, 1889				X	+				ICN-MHN 10101, 10330, 12112, 12114
*Tridensimilis venezuelae* Schultz, 1944					-			X	Ortega-Lara et al. (2012)
*Tridentopsis pearsoni Myers, 1925				X					CZUT-IC 17989, 18069
*Haemomaster venezuelae* Myers, 1927					X				Maldonado-Ocampo et al. (2006a)
*Henonemus punctatus* (Boulenger, 1887)				X					Mojica et al. (2005)
*Henonemus triacanthopomus* DoNascimiento & Provenzano, 2006					X				Lasso et al. (2009) | CZUT-IC 9648
*Megalocentor echthrus* de Pinna & Britski, 1991				X	-				IAvH-P 5995
*Ochmacanthus alternus* Myers, 1927					X				Dahl (1960a)
*Ochmacanthus orinoco* Myers, 1927					X				IAvH-P 14250-14252
*Ochmacanthus reinhardtii* (Steindachner, 1882)				X					Arbeláez et al. (2004)
*Pseudostegophilus haemomyzon* (Myers, 1942)					X				IAvH-P 1478, 6997
*Pseudostegophilus nemurus* (Günther, 1869)				X	X				Mojica et al. (2005) | IAvH-P 1189, 4996
*Schultzichthys bondi* (Myers, 1942)					X				Maldonado-Ocampo et al. (2008)
*Schultzichthys gracilis* Dahl, 1960	X	X			X				Dahl (1960a) | ICN-MHN 17123
*Stegophilus septentrionalis* Myers, 1927					X				CZUT-IC 5049
*Paracanthopoma* Giltay, 1935					X				Maldonado-Ocampo et al. (2008)
*Paravandellia phaneronema* (Miles, 1943)	X	X				X			Miles (1943) | MCZ 35874 | USNM 120141
*Vandellia beccarii* Di Caporiacco, 1935					X				Maldonado-Ocampo et al. (2013a)
*Vandellia cirrhosa* Valenciennes, 1846				X					Castro and Arboleda (1988) | de Pinna and Wosiacki (2003)
**Callichthyidae44 spp (2 added and 3 deleted from 2008)**									
*Callichthys callichthys* (Linnaeus, 1758)				X	X				Mojica et al. (2005) | Cala (1977) | IAvH-P 2089, 12161 | ICN-MHN 548, 6377, 7494, 7495, 16897
*Callichthys fabricioi* Román-Valencia, Lehmann A. & Muñoz, 1999	X	X	X			X			Román-Valencia et al. (1999) | ICN-MHN 3842 | IUQ 152, 305, 306, 307 | MCP 21174-75 | UV 89018, 91063, 93001-04, 98002, 98031
*Callichthys oibaensis* Ardila-Rodríguez, 2006	X	X	X			X			Ardila Rodríguez (2006b) | CAR 251, 250 | IMCN 3310 | CZUT-IC 1837 | IAvH-P 5730 | ICNMNH 13396 | MBUCV-V 32798
*Dianema longibarbis* Cope, 1872				X					Mojica et al. (2005)
*Hoplosternum littorale* (Hancock, 1828)				X	X				Mojica et al. (2005) | Cala (1977)
*Hoplosternum magdalenae* Eigenmann, 1913						X		X	Eigenmann (1913) | Eigenmann (1922)
*Hoplosternum punctatum* Meek & Hildebrand, 1916								X	Maldonado-Ocampo et al. (2006b)
*Lepthoplosternum altamazonicum* Reis, 1997				X					Galvis et al. (2007b)
*Megalechis picta* (Müller & Troschel, 1849)				X	X				Mojica et al. (2005) | Maldonado-Ocampo et al. (2006a)
*Megalechis thoracata* (Valenciennes, 1840)				X	X				Mojica et al. (2005) | Galvis et al. (2007a)
*Corydoras aeneus* (Gill, 1858)					X				Nijssen and Isbrücker (1983) | Cala (1977)
*Corydoras agassizii* Steindachner, 1876				X					Castro (1987b)
*Corydoras ambiacus* Cope, 1872				X					Castro (1987b)
*Corydoras arcuatus* Elwin, 1939				X					Castro (1987b)
*Corydoras axelrodi* Rössel, 1962				-	X				Nijssen and Isbrücker (1983)
*Corydoras brevirostris* Fraser-Brunner, 1947					X				Galvis et al. (2007a)
*Corydoras concolor Weitzman, 1961					X				CZUT-IC 11794, 11892
*Corydoras crypticus Sands, 1995				X					IMCN 5833, IMCN 5834 | CZUT-IC 11837
*Corydoras delphax* Nijssen & Isbrücker, 1983	X	X		-	X				Nijssen and Isbrücker (1983) | NRM 26073 [1972222.3251] | NRM 26074 | ZMA 119063
*Corydoras elegans* Steindachner, 1876				X					Castro (1987b)
*Corydoras esperanzae* Castro, 1987	X	X			X				Castro (1987b) | UBJTL MM275
*Corydoras evelynae* Rössel, 1963				X					Galvis et al. (2007b)
*Corydoras fowleri* Böhlke, 1950				X					Mojica (1999). | IMCN 5710 | NRM 16554
*Corydoras gomezi* Castro, 1986				X					Castro (1986) | UBJTL MM536
*Corydoras habrosus* Weitzman, 1960					X				Castro (1987b)
*Corydoras leucomelas* Eigenmann & Allen, 1942				X					Castro (1987b)
*Corydoras loxozonus* Nijssen & Isbrücker, 1983	X	X		-	X				Nijssen and Isbrücker (1983) | ANSP 150170
*Corydoras melanistius* Regan, 1912					X				Castro (1987b)
*Corydoras melanotaenia* Regan, 1912	X	X		-	X				Regan (1912b) | BMNH 1909.7.23.41 | BMNH 1909.7.23.42
*Corydoras melini* Lönnberg & Rendahl, 1930				X	X				Regan (1912b) | Nijssen and Isbrücker (1983)
*Corydoras metae* Eigenmann, 1914	X	X		-	X				Eigenmann (1914) | CAS 36447 [ex IU 13451]
*Corydoras osteocarus* Böhlke, 1951					X				Galvis et al. (2007a)
*Corydoras pastazensis* Weitzman, 1963				X					Mojica et al. (2005)
*Corydoras pygmaeus* Knaack, 1966				X					Galvis et al. (2007b)
*Corydoras rabauti* La Monte, 1941				X					Castro (1987b)
*Corydoras reticulatus* Fraser-Brunner, 1938				X					Castro (1987b)
*Corydoras reynoldsi* Myers & Weitzman, 1960	X	X		X					Myers and Weitzman (1960) | SU 52349, 50702 | ZMA 111424
*Corydoras semiaquilus* Weitzman, 1964				X					Myers and Weitzman (1960) | Arbeláez et al. (2004)
*Corydoras septentrionalis* Gosline, 1940					X				Isbrücker (1999)
*Corydoras simulatus* Weitzman & Nijssen, 1970	X	X		-	X				Weitzman and Nijssen 1970) | USNM 197615, 197616, 197667 | ZMA 110384
*Corydoras sodalis* Nijssen & Isbrücker, 1986				X					Castro (1987b)
*Corydoras splendens* (Castelnau, 1855)				X					Mojica et al. (2005)
*Corydoras trilineatus* Cope, 1872				X					Nijssen and Isbrücker (1983)
*Corydoras zygatus* Eigenmann & Allen, 1942				X					Mojica et al. (2005)
**Astroblepidae38 spp (16 added and 3 deleted from 2008)**									
*Astroblepus acostai* Ardila Rodríguez, 2011	X	X						X	Ardila Rodríguez (2011e) | CAR 473, 422, | ICN-MHN 17843
*Astroblepus ardiladuartei* Ardila Rodríguez, 2015	X	X				X			Ardila Rodríguez (2015a)
*Astroblepus ardilai* Ardila Rodríguez, 2012	X	X				X			Ardila Rodríguez (2012) | CAR 619, 539, 548, 423, 616 | ICN-MHN 18326 | IMCN 5260
*Astroblepus bellezaensis* Ardila Rodríguez, 2015	X	X				X			Ardila Rodríguez (2015a)
*Astroblepus cacharas* Ardila Rodríguez, 2011	X	X				X			Ardila Rodríguez (2011c) | CAR 460
*Astroblepus caquetae* Fowler 1943	X	X		X					Fowler (1943) | ANSP 70506, 70507-08
*Astroblepus chapmani* (Eigenmann, 1912)	X	X				X	-	-	Eigenmann (1912) | CAS 64658 [ex IU 12708a-c] | FMNH 56071 [ex CM 4863], 50672
*Astroblepus chotae* (Regan, 1904)						-	X	-	Maldonado-Ocampo et al. (2013b)
*Astroblepus cirratus* (Regan, 1912)	X	X				-	X		Regan (1912c) | BMNH 1912.3.2.7
*Astroblepus curitiensis* Ardila Rodríguez, 2015	X	X				X			Ardila Rodríguez (2015b)
*Astroblepus floridablancaensis* Ardila Rodríguez, 2016	X	X				X			Ardila Rodríguez (2016b) | CAR 660
*Astroblepus frenatus* Eigenmann, 1918	X	X			-	X		-	Eigenmann (1918a) | FMNH 58384 [ex CM 7380]
*Astroblepus grixalvii* Humboldt, 1805	X	X				X	-	-	Humboldt (1805)
*Astroblepus guentheri* (Boulenger, 1887)	X	X				X	-		Boulenger (1887) | BMNH 1880.2.26.18-25
*Astroblepus heterodon* (Regan, 1908)	X	X					X		Regan (1908) | BMNH 1908.5.29.80
*Astroblepus homodon* (Regan, 1904)	X	X				X	-		Regan (1904) | BMNH 1902.5.15.27
*Astroblepus itae* Ardila Rodríguez, 2011	X	X				X			Ardila Rodríguez (2011e) | CAR 555, 432, 433, 435 | ICN-MHN 7844
*Astroblepus jimenezae* Ardila Rodríguez, 2013	X	X						X	Ardila Rodríguez (2013a) | CAR 470, 542 | CIUA 756, 757, 758;
*Astroblepus jurubidae* Fowler, 1944	X	X					X		Fowler (1944) | ANSP 71431
*Astroblepus latidens* Eigenmann, 1918	X	X			+	-	-		Eigenmann (1918a) | FMNH 58366 [ex CM 7362], 58367-71, 69816 | CAS 64659 [ex IU 13678], 64689 [ex IU 13679]
*Astroblepus mariae* (Fowler, 1919)	X	X			X	-			Fowler (1919) | ANSP 49368, 49369-84
*Astroblepus marmoratus* (Regan, 1904)	X	X				X			Regan (1904) | BMNH 1899.8.21.10-11
*Astroblepus martinezi* Ardila Rodríguez, 2013	X	X						X	Ardila Rodríguez (2013a) | CAR 550,421 | MBUCV 35674
*Astroblepus micrescens* Eigenmann, 1918	X	X			-	X			Eigenmann (1918a) | CAS 64692 [ex IU 13686] | FMNH 58376 [ex CM 7372], 58377 [ex CM 7373]
*Astroblepus mojicai* Ardila Rodríguez, 2015	X	X					X		Ardila Rodríguez (2015a)
*Astroblepus nettoferreirai* Ardila Rodríguez, 2015	X	X				X			Ardila Rodríguez (2015a)
*Astroblepus nicefori* Myers, 1932	X	X			-	X	-		Myers (1932) | SU 24796 | USNM 94178
*Astroblepus onzagaensis* Ardila Rodríguez, 2015	X	X				X			Ardila Rodríguez (2015b)
*Astroblepus orientalis* (Boulenger, 1903)								X	Ortega-Lara et al. (2012)
*Astroblepus pradai* Ardila Rodríguez, 2015	X	X				X			Ardila Rodríguez (2015b)
*Astroblepus putumayoensis* Ardila Rodríguez, 2015	X	X		X					Ardila Rodríguez (2015a)
*Astroblepus rengifoi* Dahl, 1960	X	X						X	Dahl (1960c)
*Astroblepus retropinnus* (Regan, 1908)	X	X					X		Regan (1908) | BMNH 1908.5.29.81–82 | IMCN 295
*Astroblepus santanderensis* Eigenmann, 1918	X	X				X			Eigenmann (1918a) | FMNH 58433 [ex CM 7430], 58372-75, 58427-28, 58431-32 | CAS 47001 [ex IU 13741], 47002 [ex IU 13682], 60163 [ex IU 13740], 64696 [ex IU 13683], 64697 [ex IU 13684], 64698 [ex IU 13685]
*Astroblepus trifasciatus* (Eigenmann, 1912)	X	X				-	X	-	Eigenmann (1912) | AMNH 18667 [ex IU 12711] | CAS 64716-17 [ex IU 12711-12] | FMNH 56076 [ex CM 4868], 56077-78 | ?64718 | USNM 79198
*Astroblepus unifasciatus* (Eigenmann, 1912)	X	X				-	X	-	Eigenmann (1912) | FMNH 56079 [ex CM 4871], 56080, 69817 | CAS 64718 [ex IU 12713] | USNM 79199
*Astroblepus ventralis* (Eigenmann, 1912)	X	X				-	X		Eigenmann (1912) | FMNH 56074 [ex CM 4866], 56073, 56075 | AMNH 18666 [ex IU 12710] (1) | CAS 47013 [ex CM 4865, IU 12709], 47014 [ex CM 4867a-g, IU 12710a-g]
*Astroblepus verai* Ardila Rodríguez, 2015	X	X				X			Ardila Rodríguez (2015b)
**Loricariidae187 spp (30 added and 24 deleted from 2008)**									
*Pseudorinelepis genibarbis* (Valenciennes, 1840)				+	X				Ortega-Lara (2016) | Galvis et al. (2007a)
*Acestridium colombiensis* Retzer, 2005	X	X			X				Retzer (2005) | FMNH 115255, 105169 | INHS 99093 | USNM 381314
*Acestridium dichromum Retzer, Nico & Provenzano, 1999				X	X				IAvH-P 9946-9948, 10704
*Acestridium martini* Retzer, Nico & Provenzano, 1999					X				Lasso et al. (2005) | IAvH-P 9949-9951 | ICN-MHN 5333, 6328, 12380-12383,12817,12818, 12384, 12819
*Hypoptopoma bianale* Aquino & Schaefer, 2010				X					Aquino and Schaefer (2010)
*Hypoptopoma brevirostratum* Aquino & Schaefer, 2010				X					Aquino and Schaefer (2010)
*Hypoptopoma gulare* Cope, 1878				X					Aquino and Schaefer (2010)
*Hypoptopoma machadoi* Aquino & Schaefer, 2010					X				Aquino and Schaefer (2010)
*Hypoptopoma spectabile* (Eigenmann, 1914)				X	X				Eigenmann (1914) | Aquino and Schaefer (2010)
*Hypoptopoma steindachneri* Boulenger, 1895				+	-				Aquino and Schaefer (2010)
*Hypoptopoma sternoptychum* (Schaefer, 1996)				X					Schaefer (1996)
*Hypoptopoma thoracatum* Günther, 1868				X					Aquino and Schaefer (2010)
*Otocinclus batmani* Lehmann A., 2006				X					Lehmann (2006) | ICN-MHN 6721, 6722 | ANSP 178616 | MCP 28172, 34087 | MHNUC 474
*Otocinclus huaorani* Schaefer, 1997				X	X				Galvis et al. (2007b) | Maldonado-Ocampo et al. (2013a)
*Otocinclus macrospilus* Eigenmann & Allen, 1942				X					Mojica et al. (2005)
*Otocinclus vestitus* Cope, 1872				X					Mojica et al. (2005)
*Otocinclus vittatus* Regan, 1904					X				Galvis et al. (2007a)
*Oxyropsis acutirostra* Miranda Ribeiro, 1951				-	X				Aquino and Schaefer (2002)
*Oxyropsis carinata* (Steindachner, 1879)				X					Mojica et al. (2005)
*Oxyropsis wrightiana* Eigenmann & Eigenmann, 1889				X					Aquino and Schaefer (2002)
*Parotocinclus eppleyi* Schaefer & Provenzano, 1993					X				Maldonado-Ocampo et al. (2006a)
*Parotocinclus variola* Lehmann, Schvambach & Reis, 2015	X	X		X					Lehmann et al. (2015)
*Farlowella acus* (Kner, 1854)					X				MPUJ 6067, 6083, 6089, 6095
*Farlowella amazona* (Günther, 1864)				X					Arbeláez et al. (2004)
*Farlowella colombiensis* Retzer & Page, 1997	X	X		-	X				Retzer and Page (1997) | ANSP 88080 | CAS 123733, 169739 [ex CAS 123733] | ICN-MHN 17113 | INHS 32939 [ex ASNP 88080];
*Farlowella curtirostra* Myers, 1942								X	Ortega-Lara et al. (2012)
*Farlowella gracilis* Regan, 1904	X	X		X	-				Regan (1904) | BMNH 1902.5.29.180
*Farlowella mariaelenae* Martín Salazar, 1964					X				Retzer and Page (1997)
*Farlowella mitoupibo* Ballen, Urbano-Bonilla & Zamudio, 2016	X	X			X				Ballen et al. (2016b) | MPUJ 8481
*Farlowella nattereri* Steindachner, 1910				X					Retzer and Page (1997)
*Farlowella oxyrryncha* (Kner, 1853)				X	-				Arbeláez et al. (2004)
*Farlowella smithi* Fowler, 1913				X					Bogotá-Gregory and Maldonado-Ocampo (2006a) | ICN-MHN 5748 | IAvH-P 490
*Farlowella taphorni Retzer & Page, 1997								X	IAvH-P 9800, 9801
*Farlowella vittata* Myers, 1942					X				Retzer and Page (1997) | Cala (1977)
*Farlowella yarigui* Ballen & Mojica, 2014	X	X				X			Ballen and Mojica (2014) | ICN-MHN 17819, 17789, 18889
*Lamontichthys filamentosus (La Monte, 1935)				X					IAvH-P 1610, 1617
*Lamontichthys llanero* Taphorn & Lilyestrom, 1984					X				Urbano-Bonilla et al. (2009).
*Lamontichthys maracaibero* Taphorn & Lilyestrom, 1984								X	Ortega-Lara et al. (2012)
*Sturisoma caquetae* (Fowler, 1945)	X	X		X					Fowler (1945) | ANSP 71719
*Sturisoma tenuirostre* (Steindachner, 1910)					X				Cala (1977)
*Sturisomatichthys aureus* (Steindachner, 1900)	X	X				X	-	X	Steindachner (1900) | Maldonado-Ocampo et al. (2006b)
*Sturisomatichthys festivus* (Myers, 1942)								X	Ortega-Lara et al. (2012)
*Sturisomatichthys leightoni* (Regan, 1912)	X	X				X	X		Regan (1912c) | Eigenmann (1922) | BMNH 1909.7.23.45, 1909.7.23.46-4
*Sturisomatichthys panamensis* (Eigenmann & Eigenmann, 1889)						X	X	X	Eigenmann (1922) | Regan (1913)
*Sturisomatichthys tamanae* (Regan, 1912)	X	X				-	X	-	Regan (1912c) | BMNH 1910.7.11.133, BMNH 1910.7.11.134
*Crossoloricaria cephalaspis* Isbrücker, 1979	X	X			-	+			Isbrücker (1979) | BMNH 1947.7.1.228
*Crossoloricaria variegata* (Steindachner, 1879)						X	X	X	Eigenmann (1922) | Regan (1914)
*Crossoloricaria venezuelae* (Schultz, 1944)								X	Ortega-Lara et al. (2012)
*Dasyloricaria filamentosa* (Steindachner, 1878)						X		X	Steindachner (1878) | Londoño-Burbano and Reis (2016)
*Dasyloricaria latiura* (Eigenmann & Vance, 1912)							-	X	Eigenmann (1912) | FMNH 55115 | USNM 79219
*Dasyloricaria paucisquama* Londoño-Burbano & Reis, 2016	X	X				X			Londoño-Burbano and Reis (2016)
*Dentectus barbarmatus* Mártin Salazar, Isbrücker & Nijssen, 1982					X				Urbano-Bonilla et al. (2009).
*Hemiodontichthys acipenserinus* (Kner, 1853)				X					Mojica et al. (2005)
*Limatulichthys griseus* (Eigenmann, 1909)				X	X				Mojica et al. (2005) | Maldonado-Ocampo et al. (2006a)
*Loricaria cataphracta* Linnaeus, 1758				X	X				Galvis et al. (2007b) | Galvis et al. (2007a)
*Loricaria nickeriensis* Isbrücker, 1979				X					Mojica et al. (2005)
*Loricaria simillima* Regan, 1904					X				Urbano-Bonilla et al. (2009).
*Loricariichthys brunneus* (Hancock, 1828)					X				Galvis et al. (2007a)
*Loricariichthys hauxwelli (Hancock, 1828)				X					IAvH-P 12562
*Pseudohemiodon* Bleeker, 1862					X				Urbano-Bonilla et al. (2009).
*Rhadinoloricaria laani* (Nijssen & Isbrücker, 1988)				-	X				Nijssen and Isbrücker (1988) | ANSP 131483, 131484, 157750, 157940-41
*Rhadinoloricaria listrorhinos* (Nijssen & Isbrücker, 1988)	X	X		-	X				Nijssen and Isbrücker (1988) | ANSP 131482
*Rhadinoloricaria rhami* (Isbrücker & Nijssen, 1983)				X					Mojica et al. (2005)
*Rineloricaria castroi* Isbrücker & Nijssen, 1984				X					Arbeláez et al. (2004)
*Rineloricaria daraha Rapp Py-Daniel & Fichberg, 2008				X					Bogotá-Gregory et al. (2016)
*Rineloricaria eigenmanni* (Pellegrin, 1908)					X				Galvis et al. (2007a)
*Rineloricaria formosa* Isbrücker & Nijssen, 1979				-	X				Isbrücker and Nijssen (1979) | FMNH 83713, 83714-15 | IRSNB 608-609 | MZUSP [ex IRSNB 608, MZUSP ex IRSNB 609] | ZMA 114922-23, 115182, 115196-97 | ZSM 25821
*Rineloricaria jubata* (Boulenger, 1902)							X	X	Eigenmann (1922) | Regan (1914)
*Rineloricaria lanceolata* (Günther, 1868)				X					Arbeláez et al. (2004)
*Rineloricaria magdalenae* (Steindachner, 1879)						X	-	X	Steindachner (1879a) | Eigenmann (1922)
*Rineloricaria rupestris* (Schultz, 1944)								X	Ortega-Lara et al. (2012)
*Rineloricaria sneiderni* (Fowler, 1944)	X	X					X		Fowler (1944) | ANSP 71433, 71434
*Spatuloricaria atratoensis* Schultz, 1944	X	X						X	Schultz (1944b) | USNM 93810
*Spatuloricaria caquetae* (Fowler, 1943)	X	X		X					Fowler (1943) | ANSP 70526, 70527
*Spatuloricaria curvispina* (Dahl, 1942)	X	X				X			Dahl (1942)
*Spatuloricaria euacanthagenys* Isbrücker, 1979	X	X		X					Fowler (1943)
*Spatuloricaria gymnogaster* (Eigenmann & Vance, 1912)	X	X				X		X	Eigenmann (1912) | Villa-Navarro et al. (2006)
*Spatuloricaria fimbriata* (Eigenmann & Vance, 1912)						X		X	Eigenmann (1912) | Miles (1947)
*Spatuloricaria lagoichthys* (Schultz, 1944)								X	Miles (1947)
*Spatuloricaria phelpsi* Schultz, 1944								X	Ortega-Lara et al. (2012)
*Acanthicus hystrix* Spix & Agassiz, 1829				X	X				Bogotá-Gregory and Maldonado-Ocampo (2005) | Cala (1977) | ICN-MHN 596 | IAvH-P 2301
*Baryancistrus beggini* Lujan, Arce & Armbruster, 2009					X				Lujan et al. (2009)
*Baryancistrus demantoides* Werneke, Sabaj Pérez, Lujan & Armbruster, 2005					X				Ajiaco-Martínez et al. (2012c) | Ortega-Lara (2016)
*Chaetostoma aburrense* (Posada, 1909)	X	X				X			Posada (1909)
*Chaetostoma anale* (Fowler, 1943)	X	X		X					Fowler (1943) | ANSP 70525
*Chaetostoma anomalum* Regan, 1903								X	Ortega-Lara et al. (2012)
*Chaetostoma brevilabiatum* Dahl, 1942	X	X				X			Dahl (1943)
*Chaetostoma dorsale* Eigenmann, 1922					X				Urbano-Bonilla et al. (2009).
*Chaetostoma fischeri* Steindachner, 1879						X	X	X	Eigenmann (1922)
*Chaetostoma floridablancaense* Ardila Rodríguez, 2013	X	X				X			Ardila Rodríguez (2013b) | CAR 633, 104, 105, 417 | MBUCV 35676 | IMCN 5676
*Chaetostoma formosae* Ballen, 2011	X	X			X				Ballen (2011) | ICN-MHN 17114
*Chaetostoma joropo* Ballen, Urbano-Bonilla & Maldonado-Ocampo, 2016	X	X			X				Ballen et al. (2016a)
*Chaetostoma lepturum* Regan, 1912	X	X					X		Regan (1912c) | BMNH 1910.7.11.116–118
*Chaetostoma leucomelas* Eigenmann, 1918	X	X				X	X	X	Eigenmann (1918a) | Maldonado-Ocampo et al. (2006b) | Miles (1947)
*Chaetostoma marginatum* Regan, 1904						-	X		Regan (1913)
*Chaetostoma milesi* Fowler, 1941	X	X			-	X			Fowler (1941) | ANSP 69330
*Chaetostoma niveum* Fowler, 1944	X	X					X		Fowler (1944) | ANSP 71432
*Chaetostoma palmeri* Regan, 1912	X	X					X		Regan (1912c) | BMNH 1910.7.11.120–121
*Chaetostoma patiae* Fowler, 1945	X	X					X		Fowler (1945) | ANSP 71716, 71717
*Chaetostoma paucispinis* Regan, 1912	X	X					X		Regan (1912c) | BMNH 1910.7.11.119
*Chaetostoma platyrhynchus* (Fowler, 1943)				X	+				Fowler (1943) | ANSP 70512, 70513-15
*Chaetostoma setosum* Boulenger, 1887	X	X			-	+			Boulenger (1887) | BMNH 1880.2.26.9-10
*Chaetostoma sovichthys* Schultz, 1944								X	Ballen (2011)
*Chaetostoma tachiraense* Schultz, 1944								X	Ortega-Lara et al. (2012)
*Chaetostoma thomsoni* Regan, 1904	X	X				X			Regan (1904) | BMNH 1902.5.15.28-30
*Chaetostoma vagum* Fowler, 1943	X	X		X					Fowler (1943)
*Cordylancistrus daguae* (Eigenmann, 1912)	X	X				-	X		Eigenmann (1912) | FMNH 56052 [ex CM 4842], 56053-54 | CAS 56745 [ex IU 12698], 74161 [ex IU 12699]
*Cordylancistrus pijao* Provenzano R. & Villa-Navarro, 2017	X	X				X			Provenzano R. and Villa-Navarro (2017)
*Dolichancistrus atratoensis* (Dahl, 1960)	X	X						X	Dahl (1960c) | ICN-MHN 51, 46 (now apparently 48)
*Dolichancistrus carnegiei* (Eigenmann, 1916)	X	X				X			Eigenmann (1916) | FMNH 58350 [ex CM 7346], 58351 | CAS 77344 [ex IU 13661], 77345 [ex IU 13662]
*Dolichancistrus cobrensis* (Schultz, 1944)					+			X	Ballen and Vari (2012) | ICN-MHN 18009
*Dolichancistrus fuesslii* (Steindachner, 1911)	X	X			X				Steindachner (1911) | NMW 48026
‘*Hemiancistrus’ guahiborum* Werneke, Armbruster, Lujan & Taphorn, 2005					X				Maldonado-Ocampo et al. (2006a)
*‘*Hemiancistrus’ subviridis* Werneke, Sabaj, Lujan & Armbruster, 2005					X				IMCN 5897, 6094, 6189, 6195, 6196, 6284, 6574, 6575
*Hypancistrus contradens* Armbruster, Lujan & Taphorn, 2007					X				Maldonado-Ocampo et al. (2008)
*Hypancistrus debilittera* Armbruster, Lujan & Taphorn, 2007					X				Ortega-Lara (2016) | IAvH-P 12471
*Hypancistrus furunculus Armbruster, Lujan & Taphorn, 2007					X				Ortega-Lara (2016) | IMCN 5828, 6017, 6018, 6297, 6298, 6299, 6300
*Hypancistrus inspector Armbruster, 2002					X				Ortega-Lara (2016)
*Hypancistrus lunaorum* Armbruster, Lujan & Taphorn, 2007					X				Maldonado-Ocampo et al. (2008) | IAvH-P 7044
*Leporacanthicus galaxias* Isbrücker & Nijssen, 1989					X				IAvH-P 7041
*Leporacanthicus triactis Isbrücker, Nijssen & Nico, 1992					X				IMCN 5772, 5773, 5774
*Leptoancistrus canensis (Meek & Hildebrand, 1913)								X	Maldonado-Ocampo et al. (2013b)
*Leptoancistrus cordobensis* Dahl, 1964	X	X				X		X	Dahl and Medem (1964) | IMCN 233, 250
*Panaque cochliodon* (Steindachner, 1879)	X	X	X			X			Steindachner (1879b) | NMW 47297-98
*Panaque nigrolineatus* (Peters, 1877)					X				Lujan et al. (2010) | Cala (1977)
*Panaque suttonorum* Schultz, 1944								X	Lujan et al. (2010)
*Panaque titan Lujan, Hidalgo & Stewart 2010				X					IMCN 6059
*Panaqolus albomaculatus* (Kanazawa, 1958)				X					Galvis et al. (2007b)
*Panaqolus maccus* Schaefer & Stewart, 1993					X				Urbano-Bonilla et al. (2009).
*Peckoltia brevis* (La Monte, 1935)				X					Armbruster (2008)
*Peckoltia caenosa Armbruster, 2008					X				IMCN 7061
*Peckoltia lineola* Armbruster, 2008					X				Armbruster (2008)
*Peckoltia lujani* Armbruster, Werneke & Tan, 2015					X				Armbruster et al. (2015)
*Peckoltia sabaji* Armbruster, 2003					X				Urbano-Bonilla et al. (2009).
*Peckoltia vittata* (Steindachner, 1881)					X				Armbruster (2008)
*Peckoltichthys bachi* (Boulenger, 1898)				X					Armbruster (2008)
*Parancistrus* Bleeker, 1862					X				Galvis et al. (2007a)
*Pseudoancistrus sidereus Armbruster, 2004					X				IMCN 6232, 6234
*Aphanotorulus ammophilus* Armbruster & Page, 1996					X				Maldonado-Ocampo et al. (2008)
*Aphanotorulus emarginatus* (Valenciennes, 1840)				X	X				Bogotá-Gregory and Maldonado-Ocampo (2006a) | Ray and Armbruster (2016) | ICN-MHN 2543
*Aphanotorulus horridus* (Kner, 1854)				X					Ray and Armbruster (2016)
*Aphanotorulus unicolor* (Steindachner, 1908)				X					Ray and Armbruster (2016)
*Hypostomus annectens* (Regan, 1904)							X		Eigenmann (1922)
*Hypostomus argus* (Fowler, 1943)				-	X				Fowler (1943) | ANSP 70510
*Hypostomus carinatus* (Steindachner, 1881)				X					Maldonado-Ocampo et al. (2008)
*Hypostomus hemicochliodon* Armbruster, 2003				X					Lasso et al. (2009) | IAvH-P 8910
‘*Hypostomus’ holostictus* (Regan, 1913)	X	X					X	X	Regan (1913) | BMNH 1913.10.1.57
*Hypostomus hondae* (Regan, 1912)			X			X	X	X	Regan (1912c) | Dahl (1955) | Eigenmann (1922)
*Hypostomus niceforoi* (Fowler, 1943)	X	X		X	X				Fowler (1943) | Urbano-Bonilla et al. (2009).
*Hypostomus oculeus* (Fowler, 1943)				X					Fowler (1943) | ANSP 70518, 70519-20
*Hypostomus plecostomoides* (Eigenmann, 1922)				X	X				Eigenmann (1922) | CAS 82501 [ex IU 15043] | IAvH-P 3045
*Hypostomus plecostomus* (Linnaeus, 1758)					X				Eigenmann (1922)
*Hypostomus pyrineusi* (Miranda Ribeiro, 1920)				X	X				Mojica et al. (2005) | Maldonado-Ocampo et al. (2008)
*Hypostomus robinii Valenciennes, 1840					X				IAvH-P 423, 736, 4957, 5372, 5373, 5495, 5502, 5504
*Hypostomus sculpodon* Armbruster, 2003					X				IAvH-P 5614
*Hypostomus varimaculosus* (Fowler, 1945)				X					Fowler (1945) | ANSP 71707
*Hypostomus wilsoni* (Eigenmann, 1918)	X	X					X	X	Eigenmann (1918a) | Dahl (1955)
*Isorineloricaria tenuicauda* (Steindachner, 1878)	X	X				X			Steindachner (1878) | MSNG 8856 | NMW 42596, 44263-66, 44268), 44294 | ZMUC 85
*Isorineloricaria villarsi* (Lütken, 1874)					-			+	Ray and Armbruster (2016)
*Pterygoplichthys gibbiceps* (Kner, 1854)					X				Maldonado-Ocampo et al. (2006a)
*Pterygoplichthys lituratus* (Kner, 1854)				X					Mojica et al. (2005)
*Pterygoplichthys multiradiatus* (Hancock, 1828)					X				Maldonado-Ocampo et al. (2013a)
*Pterygoplichthys pardalis* (Castelnau, 1855)				X					Mojica et al. (2005)
*Pterygoplichthys undecimalis* (Steindachner, 1878)	X	X			-	X			Steindachner (1878) | NMW 47224, 47220-22
*Pterygoplichthys weberi* Armbruster & Page, 2006				X					Armbruster and Page (2006) | ICN-MHN 13455 | FMNH 96959, 101378 | MSU 2736.2 | USNM 177204
*Pterygoplichthys zuliaensis Weber, 1991								X	Ortega-Lara et al. (2012)
*Ancistrus caucanus* Fowler, 1943	X	X				X			Fowler (1943) | ANSP 70516
*Ancistrus centrolepis* Regan, 1913	X	X				-	X	X	Regan (1913) | Taphorn et al. (2013)
*Ancistrus lineolatus* Fowler, 1943	X	X		X					Fowler (1943) | ANSP 70517
*Ancistrus macrophthalmus* (Pellegrin, 1912)					X				Maldonado-Ocampo et al. (2006a)
*Ancistrus martini* Schultz, 1944								X	Taphorn et al. (2013)
*Ancistrus tolima* Taphorn, Armbruster, Villa-Navarro & Ray, 2013	X	X				X			Taphorn et al. (2013) | CZUT-IC 4040
*Ancistrus triradiatus* Eigenmann, 1918				-	X			-	Eigenmann (1918a)
*Ancistrus vericaucanus* Taphorn, Armbruster, Villa-Navarro & Ray, 2013	X	X				X			Taphorn et al. (2013)
*Dekeyseria amazonica* Rapp Py-Daniel, 1985				X					Mojica et al. (2005)
*Dekeyseria brachyura* (Kner, 1854)					X				Ortega-Lara (2016)
*Dekeyseria pulchra* (Steindachner, 1915)					X				Galvis et al. (2007a)
*Dekeyseria scaphirhyncha* (Kner, 1854)					X				Maldonado-Ocampo et al. (2006a)
*Lasiancistrus caucanus* Eigenmann, 1912						X	X	X	Eigenmann (1912) | Fowler (1945) | Dahl (1955)
*Lasiancistrus guacharote* (Valenciennes, 1840)								X	Ortega-Lara et al. (2012)
*Lasiancistrus schomburgkii* (Günther, 1864)				X					Armbruster (2005)
*Lasiancistrus tentaculatus* Armbruster, 2005					X				Armbruster (2005) | IAvH-P 5100-5105, 7692, 7982, 7983, 9487, 9595, 9596
*Lithoxancistrus orinoco* Isbrücker, Nijssen & Cala, 1988					X				Isbrücker et al. (1988) | ICN-MHN 1200, 1201 | ZMA 119882 | ZMUL 972/3482
*Pseudolithoxus anthrax (Armbruster & Provenzano, 2000)					X				IMCN 6601, 6602, 6603,6604, 5874, 5880, 5881, 6063, 6064, 6231
*Pseudolithoxus dumus* (Armbruster & Provenzano, 2000)					X				Maldonado-Ocampo et al. (2006a)
*Pseudolithoxus kelsorum Lujan & Birindelli, 2011					X				IMCN 5876, IMCN 5877
*Pseudolithoxus tigris* (Armbruster & Provenzano, 2000)					X				Maldonado-Ocampo et al. (2006a)
**Cetopsidae15 spp (4 deleted from 2008)**									
*Cetopsidium morenoi* (Fernández-Yépez, 1972)					X				Vari et al. (2005)
*Cetopsidium pemon* Vari, Ferraris & de Pinna, 2005					X				Vari et al. (2005)
*Cetopsis amphiloxa* (Eigenmann, 1914)							X	X	Eigenmann et al. (1914) | Vari et al. (2005)
*Cetopsis baudoensis* (Dahl, 1960)	X	X					X		Dahl (1960c) | ICN-MHN 118, 100
*Cetopsis candiru* Spix & Agassiz, 1829				X					Mojica et al. (2005)
*Cetopsis coecutiens* (Lichtenstein, 1819)				X	X				Vari et al. (2005) | Dahl (1960a)
*Cetopsis fimbriata* Vari, Ferraris & de Pinna 2005	X	X						X	Vari et al. (2005) | USNM 305348, 372825, 372826 | ICN-MHN 7272
*Cetopsis jurubidae* (Fowler, 1944)	X	X					X		Fowler (1944) | ANSP 71430
*Cetopsis motatanensis* (Schultz, 1944)								X	Ortega-Lara et al. (2012)
*Cetopsis orinoco* (Schultz, 1944)					X				Cala (1977); Vari et al. (2005)
*Cetopsis othonops* (Eigenmann, 1912)	X	X				X		X	Eigenmann (1912) | Vari et al. (2005)
*Cetopsis umbrosa* Vari, Ferraris & de Pinna 2005	X	X			X				Vari et al. (2005)
**Denticetopsis seducta Vari, Ferraris & de Pinna 2005					X				Vari et al. (2005)
*Helogenes castaneus* (Dahl, 1960)	X	X			X				Dahl (1960a)
*Helogenes marmoratus* Günther, 1803				X	X				Mojica et al. (2005) | Maldonado-Ocampo (2001)
**Aspredinidae18 spp (2 added and 2 deleted from 2008)**									
*Amaralia hypsiura* (Kner, 1855)				X					Friel and Carvalho (2016)
*Bunocephalus aleuropsis* Cope, 1870				X	+				Cardoso (2008)
*Bunocephalus aloikae Hoedeman, 1961					X				Cardoso (2008)
*Bunocephalus colombianus* Eigenmann, 1912	X	X				X	X	X	Eigenmann (1912) | Cardoso (2008) | Miles (1945)
*Bunocephalus coracoideus* (Cope, 1874)				X					Mojica et al. (2005)
*Bunocephalus knerii* Steindachner, 1882				X					Cardoso (2008)
*Bunocephalus verrucosus* (Walbaum, 1792)				X					Bogotá-Gregory and Maldonado-Ocampo (2005)
*Pseudobunocephalus amazonicus* (Mees, 1989)				X					Friel (2008)
*Pseudobunocephalus bifidus* (Eigenmann, 1942)				X					Galvis et al. (2007b)
*Pseudobunocephalus lundbergi* Friel, 2008					X				Friel (2008)
*Pterobunocephalus depressus Hasseman, 1911				X	X				IAvH-P 674, 8714, 8779, 8814, 8844, 8872, 8903, 11991
*Dupouyichthys sapito* Schultz, 1944						X		+	Mojica et al. (2000) | Ortega-Lara et al. (2012) | Miles (1945)
*Hoplomyzon papillatus* Stewart, 1985				X					Galvis et al. (2007b)
*Hoplomyzon sexpapilostoma* Taphorn & Marrero, 1990					X				Maldonado-Ocampo et al. (2008)
*Xyliphius kryptos* Taphorn & Lilyestrom, 1983								X	Ortega-Lara et al. (2012)
*Xyliphius lepturus* Orcés, 1962					X			-	Cala (1977) | ICN-MHN 1772
*Xyliphius magdalenae* Eigenmann, 1912	X	X				X			Eigenmann (1912) | FMNH 56039 [ex CM 4829]
*Xyliphius melanopterus* Orcés, 1962				X	X				Galvis et al. (2007b) | Urbano-Bonilla et al. (2009)
**Auchenipteridae46 spp (7 added and 4 deleted from 2008)**									
*Centromochlus altae* Fowler, 1945	X	X		X	+				Fowler (1945) | ANSP 71700, 71701-04 | IAvH-P 551
*Centromochlus existimatus* Mees, 1974				X					Mojica et al. (2005)
*Centromochlus heckelii* (De Filippi, 1853)				X	X				Lasso et al. (2005) | Mojica et al. (2005) | IAvH-P 966, 1055, 3691, 10964 | ICN-MHN 952, 1927, 2716, 8549, 11950-11952, 12801
*Centromochlus macracanthus, 2000				X					IAvH-P 14318
*Centromochlus perugiae* Steindachner, 1882				X					Arbeláez et al. (2004) | IAvH-P 9270-9271, 9284, 9287, 9300-9301, 9314-9315, 9332-9333, 9348-9349, 9361-9362, 9378-9379
*Centromochlus reticulatus* (Mees, 1974)				X	+				Mojica et al. (2005) | Galvis et al. (2007a)
*Centromochlus romani* (Mees, 1988)					X				Urbano-Bonilla et al. (2009).
*Gelanoglanis stroudi* Böhlke, 1980					X				Böhlke (1980) | ANSP 142937, 142938-39, 142940, 142941 | FMNH 83911, 83912 | MZUSP 14641
*Tatia aulopygia* (Kner, 1858)				X	-				IAvH-P 9108, 9134
*Tatia dunni* (Fowler, 1945)				X					Fowler (1945) | ANSP 71705, 71706
*Tatia galaxias* Mees, 1974					X				Sarmento-Soares and Martins-Pinheiro (2008)
*Tatia gyrina* (Eigenmann & Allen, 1942)				X					Sarmento-Soares and Martins-Pinheiro (2008)
*Tatia intermedia* (Steindachner, 1877)				X	X				Sarmento-Soares and Martins-Pinheiro (2008) | IAvH-P 1232
*Tatia marthae Vari & Ferraris, 2013					X				IAvH-P 12458, 12463, 12473, 12483, 12496, 12861, 12870, 12873
*Tatia nigra Sarmento-Soares & Martins-Pinheiro, 2008				X	X				CZUT-IC 4441, 4544, IAvH-P 12862, 12874
*Tatia strigata Soares-Porto, 1995				X	X				CZUT-IC 4527, IAvH-P 5634, 10049, 10761, 12577, 12579, 12581-12583
*Ageneiosus dentatus Kner 1857					X				Galvis et al. (2007a) | Ribeiro et al. (2017)
*Ageneiosus inermis* (Linnaeus, 1766)				X	X				Mojica et al. (2005) | Dahl (1960a)
*Ageneiosus magoi* Castillo & Brull, 1989					X				Maldonado-Ocampo et al. (2013a)
*Ageneiosus pardalis* Lütken, 1874			X			X		X	Steindachner (1879a) | Dahl (1955)
*Ageneiosus ucayalensis* Castelnau, 1855				X	-				Mojica et al. (2005)
*Ageneiosus vittatus* Steindachner, 1908				X	-				ICN-MHN 5476, 5975-5976, 5978, 6605, 9178
*Asterophysus batrachus Kner, 1858					X				Ortega-Lara (2016) | IMCN 5528, 5529, 5530, 5964, 6116, 6117, 6118, 6119, 6120, 6121, 6122, 6123, 6124, 6433, 6434
*Tympanopleura atronasus* (Eigenmann & Eigenmann, 1888)				X					Mojica et al. (2005)
*Tympanopleura brevis* (Steindachner, 1881)				X					Mojica et al. (2005)
*Tympanopleura piperata* Eigenmann, 1912				X					Mojica et al. (2005)
*Auchenipterichthys coracoideus Eigenmann & Allen, 1942				X					Galvis et al. (2007b)
*Auchenipterichthys longimanus* (Günther, 1864)				X	X				Bogotá-Gregory and Maldonado-Ocampo (2006a) | Maldonado-Ocampo et al. (2006a) | IAvH-P 255, 5260
*Auchenipterichthys punctatus* (Valenciennes, 1840)				X	+				Bogotá-Gregory and Maldonado-Ocampo (2006a) | IAvH-P 254, 2393, 10008-10019
*Auchenipterus ambyiacus* Fowler, 1915				X	X				Ferraris and Vari (1999)
*Auchenipterus nuchalis* (Spix & Agassiz, 1829)				X	X				Maldonado-Ocampo et al. (2008) | Mojica et al. (2005)
*Entomocorus gameroi* Mago-Leccia, 1984					X				Reis and Borges (2006)
*Epapterus dispilurus* Cope, 1878				X					Mojica et al. (2005)
*Liosomadoras morrowi* Fowler, 1940				X					Bogotá-Gregory and Maldonado-Ocampo (2005)
*Liosomadoras oncinus* (Jardine, 1841)				X					Galvis et al. (2007b)
*Pseudepapterus cucuhyensis* Böhlke, 1951				X					Böhlke (1951)
*Pseudepapterus hasemani* (Steindachner, 1915)				X					Mojica et al. (2005)
*Tetranematichthys wallacei* Vari & Ferraris, 2006				X	X				Vari and Ferraris (2006) | IAvH-P 9084
*Trachelyichthys decaradiatus* Mees, 1974					X				Galvis et al. (2007a)
*Trachelyopterichthys anduzei* Ferraris & Fernandez, 1987					X				Maldonado-Ocampo et al. (2006a)
*Trachelyopterichthys taeniatus* (Kner, 1858)				X	X				Bogotá-Gregory and Maldonado-Ocampo (2006a) | Cala (1977) | CAS-SU 42795
*Trachelyopterus fisheri* (Eigenmann, 1916)	X	X				-		X	Eigenmann (1916) | FMNH 57695 [ex CM 6667a], 57696-98 | CAS 52136 [ex IU 13496], 57937 [ex IU 13495], 57938 [ex IU 13497] | USNM 76929
*Trachelyopterus galeatus* (Linnaeus, 1766)				X	X				Mojica et al. (2005) | Galvis et al. (2007a)
*Trachelyopterus insignis* (Steindachner, 1878)	X	X				X		X	Steindachner (1878) | Mojica et al. (2006a)
*Trachelyopterus peloichthys* (Schultz, 1944)								X	Ortega-Lara et al. (2012)
*Trachycorystes trachycorystes* (Valenciennes, 1840)					X				IAvH-P 12863, 12876, 12889
**Doradidae56 spp (7 added and 4 deleted from 2008)**									
*Acanthodoras cataphractus* (Linnaeus, 1758)				X	X				Bogotá-Gregory and Maldonado-Ocampo (2006a) | Galvis et al. (2007a) | ICN-MHN 360, 2772, 4183, 5402
*Acanthodoras depressus (Steindachner, 1881)				X					CZUT-IC 4312
*Acanthodoras spinosissimus* (Eigenmann & Eigenmann, 1888)				X	X				Mojica et al. (2005) | Maldonado-Ocampo et al. (2006a)
*Agamyxis albomaculatus* (Peters, 1877)				-	X				Galvis et al. (2007a)
*Agamyxis pectinifrons* (Cope, 1870)				X					Mojica et al. (2005)
*Astrodoras Bleeker, 1862				X					Roa-Fuentes et al. (2010)
*Amblydoras affinis* (Kner, 1855)				X	X				Mojica et al. (2005) | IAvH-P 747, 1075, 1181, 2807
*Amblydoras bolivarensis* (Fernández-Yépez, 1968)					X				Maldonado-Ocampo et al. (2006a)
*Amblydoras gonzalezi* (Fernández-Yépez 1968)				-	X				Galvis et al. (2007b)
*Amblydoras monitor* (Cope, 1872)				X					Mojica et al. (2005)
*Amblydoras nauticus* (Cope, 1874)				X					Mojica et al. (2005)
*Anadoras grypus* (Cope, 1872)				X					Mojica et al. (2005)
*Anadoras regani* (Steindachner, 1908)				X					Bogotá-Gregory and Maldonado-Ocampo (2005)
*Hypodoras forficulatus* Eigenmann, 1925				X					Mojica et al. (2005)
*Physopyxis ananas* Sousa & Rapp Py-Daniel, 2005				X	X				Maldonado-Ocampo et al. (2008)
*Physopyxis lyra* Cope, 1871				X					Mojica et al. (2005)
*Scorpiodoras heckelii* (Kner, 1855)				X	X				Correa (2003) | Galvis et al. (2007a)
*Anduzedoras oxyrhynchus* (Valenciennes, 1821)					X				Cala (1977)
*Centrochir crocodili* (Humboldt, 1821)	X	X				X			Humboldt and Valenciennes (1821)
*Centrodoras brachiatus* (Cope, 1872)				X					Bogotá-Gregory and Maldonado-Ocampo (2006a) | IAvH-P 405, 479
*Centrodoras hasemani (Steindachner, 1915)				X					CZUT-IC 14832
*Doras phlyzakion* Sabaj Pérez & Brindelli, 2008				X					Sabaj Pérez and Birindelli (2008)
*Doraops zuloagai* Schultz, 1944			X					X	Ortega-Lara et al. (2012)
*Hassar orestis* (Steindachner, 1875)				X	X				Birindelli et al. (2011) | IAvH-P 6046
*Hemidoras stenopeltis* (Kner, 1855)				X					Mojica et al. (2005)
*Hemidoras stuebelii* (Steindachner, 1882)				X					Mojica et al. (2005)
*Leptodoras acipenserinus* (Günther, 1868)				X					Maldonado-Ocampo et al. (2008)
*Leptodoras juruensis* Boulenger, 1898				X					Mojica et al. (2005)
*Leptodoras linnelli* Eigenmann, 1912					X				IAvH-P 15132
*Leptodoras nelsoni* Sabaj Pérez, 2005				X	X				Sabaj (2005) | IAvH-P 904
*Leptodoras rogersae Sabaj Pérez, 2005					X				CZUT-IC 9529
*Lithotodoras dorsalis (Valenciennes, 1840)				X					CZUT-IC 14500
*Megalodoras uranoscopus* (Eigenmann & Eigenmann, 1888)				X					Mojica et al. (2005)
*Nemadoras cristinae* Sabaj Pérez, Arce H., Sousa & Birindelli, 2014				X	X				Sabaj Pérez et al. (2014)
*Nemadoras elongatus* (Boulenger, 1898)				X					Sabaj Pérez et al. (2014)
*Nemadoras hemipeltis* (Eigenmann, 1925)				X					Sabaj Pérez et al. (2014)
*Nemadoras humeralis* (Kner, 1855)				X					Sabaj Pérez et al. (2014)
*Orinocodoras eigenmanni* Myers, 1927					X				Maldonado-Ocampo (2001)
*Opsodoras boulengeri* Steindachner, 1915				X					Mojica et al. (2005)
*Opsodoras morrisi* Eigenmann, 1925				X					Bogotá-Gregory and Maldonado-Ocampo (2006a)
*Ossancora punctata* (Kner, 1853)				X					Birindelli and Sabaj Pérez (2011)
*Oxydoras niger* (Valenciennes, 1821)				X	X				Mojica et al. (2005) | Maldonado-Ocampo et al. (2006a)
*Oxydoras sifontesi* Fernández-Yépez, 1968					X				Maldonado-Ocampo et al. (2006a) | CZUT-IC 9380
*Platydoras armatulus* (Valenciennes, 1840)				X	X				Piorski et al. (2008)
*Platydoras hancockii* (Valenciennes, 1840)					X				Cala (1977)
*Pterodoras granulosus* (Valenciennes, 1821)				X					Mojica et al. (2005)
*Pterodoras rivasi* (Fernández-Yépez, 1950)				-	X				Birindelli (2014)
*Rhinodoras boehlkei Glodek, Whitmire & Orcés V., 1976				X					CZUT-IC 12272, 12318
*Rhinodoras gallagheri* Sabaj, Taphorn & Castillo G., 2008					X				Maldonado-Ocampo et al. (2008)
*Rhinodoras thomersoni* Taphorn & Lilyestrom, 1984			X					X	Ortega-Lara et al. (2012)
*Tenellus leporhinus* (Eigenmann, 1912)				-	X				Sabaj Pérez et al. (2014)
*Tenellus ternetzi* (Eigenmann, 1925)				X	+				Sabaj Pérez et al. (2014)
*Tenellus trimaculatus* (Boulenger, 1898)				X					Mojica et al. (2005)
*Trachydoras microstomus* (Eigenmann, 1912)				-	+				IAvH-P 10752
*Trachydoras nattereri* (Steindachner, 1881)				X					Mojica et al. (2005)
*Trachydoras steindachneri* (Perugia, 1897)				X					Mojica et al. (2005)
**Ariidae1 sp**									
*Notarius bonillai* (Miles, 1945)	X	X	X			X		+	Miles (1945) | Acero and Betancur-R (2006)
**Heptapteridae52 spp (14 added and 7 deleted from 2008)**									
*Brachyrhamdia imitator Myers, 1927					X				IMCN 5691
*Brachyrhamdia meesi Sands & Black, 1985				X					IAvH-P 11146
*Brachyrhamdia thayeria Slobodian & Bockmann, 2013				X					Galvis et al. (2007b)
*Cetopsorhamdia boquillae* Eigenmann, 1922	X	X				X			Eigenmann (1922) | CAS 63607 [ex IU 15004] | FMNH 55212 [ex CM 3923], 55213 [ex CM 3924]
*Cetopsorhamdia molinae* Miles, 1943	X	X				X			Miles (1943)
*Cetopsorhamdia nasus* Eigenmann & Fisher, 1916	X	X				X			Eigenmann (1916) | FMNH 58126 [ex CM 7124]
*Cetopsorhamdia orinoco* Schultz, 1944					X				Urbano-Bonilla et al. (2009).
*Cetopsorhamdia picklei* Schultz, 1944					-			X	Ortega-Lara et al. (2012)
*Chasmocranus rosae* Eigenmann, 1922	X	X			X				Eigenmann (1922) | FMNH 55140 [ex CM 3841], 55141 [?CM 3842] | CAS 75751 [ex IU 15019]
*Gladioglanis conquistador* Lundberg, Bornbusch & Mago-Leccia, 1991				X					Galvis et al. (2007b)
*Gladioglanis machadoi Ferraris & Mago-Leccia, 1989				X	X				Maldonado-Ocampo et al. (2006a) | CZUT-IC 5091, 5126
*Goeldiella eques* (Müller & Troschel, 1848)				X	X				Correa (2003) | Maldonado-Ocampo (2001)
*Heptapterus panamensis* Bussing, 1970								X	IAvH-P 7273-7274
*Imparfinis microps* Eigenmann & Fisher, 1916	X	X			X				Eigenmann (1916) | FMNH 57793 [ex CM 6776 not 8778]
*Imparfinis nemacheir* (Eigenmann & Fisher, 1916)						X	X	X	Eigenmann (1916) | Eigenmann (1922)
*Imparfinis pristos* Mees & Cala, 1989					X				Mees and Cala (1989) | ICN-MHN 1401, 1402 | RMNH 30544
*Imparfinis pseudonemacheir* Mees & Cala, 1989					X				Mees and Cala (1989)
*Imparfinis spurrellii* (Regan, 1913)	X	X					X		Regan (1913) | BMNH 1913.10.1.41
*Imparfinis stictonotus* (Fowler, 1940)				X					Fowler (1945)
*Imparfinis timana* Ortega-Lara, Milani, DoNascimiento, Villa-Navarro & Maldonado-Ocampo, 2011	X	X				X			Ortega-Lara et al. (2011) | IAvH-P 10696
*Imparfinis usmai* Ortega-Lara, Milani, DoNascimiento, Villa-Navarro & Maldonado-Ocampo, 2011	X	X				X	X		Ortega-Lara et al. (2011) | IMCN 4812
*Mastiglanis asopos* Bockmann, 1994				X					Galvis et al. (2007b)
*Myoglanis koepckei* Chang, 1999				X					Galvis et al. (2007b)
*Nemuroglanis mariai* (Schultz, 1944)	X	X			X				Schultz (1944a) | USNM 121251
*Nemuroglanis pauciradiatus Ferraris, 1988					X				IAvH-P
*Pariolius armillatus* (Mees, 1987)				X					IAvH-P 8680, 8935, 9003, 9087, 9113, 9389, 9433
*Phenacorhamdia anisura (Mees, 1987)					X				IAvH-P 12894
*Phenacorhamdia macarenensis* Dahl, 1961	X	X			X				Dahl (1961)
*Phenacorhamdia nigrolineata* Zarske, 1998				X					Maldonado-Ocampo et al. (2008)
*Phenacorhamdia provenzanoi DoNascimiento & Milani, 2008					X				CZUT-IC 6351, 6881, 6988
*Phenacorhamdia taphorni DoNascimiento & Milani, 2008					X				IAvH-P 7932, 9254, 9594, 10727
*Pimelodella chagresi* (Steindachner, 1876)						X	X	X	Fowler (1944) | Eigenmann (1922)
*Pimelodella conquetaensis* Ahl, 1925	X	X		X					Ahl (1925) | ZMB 32030
*Pimelodella cristata* (Müller & Troschel, 1848)				X	X				Arbeláez et al. (2004) | Urbano-Bonilla et al. (2009).
*Pimelodella cruxenti Fernández-Yépez, 1950					X				CZUT-IC 7180, 7192
*Pimelodella eutaenia* Regan, 1913	X	X				-	X	X	Regan (1913) | BMNH 1913.10.1.37–40
*Pimelodella figueroai* Dahl, 1961	X	X			X				Dahl (1961)
*Pimelodella floridablancaensis* Ardila Rodríguez, 2017	X	X				X			Ardila Rodríguez (2017a)
*Pimelodella geryi* Hoedeman, 1961				X					Arbeláez et al. (2004)
*Pimelodella gracilis* (Valenciennes, 1835)				X	X				Bogotá-Gregory and Maldonado-Ocampo (2006a) | Lasso et al. (2005) | IAvH-P 1847, 1967, 3724, 3725-3729, 10004-10005 | ICN-MHN 1816, 3731, 1909, 2179, 5512, 5654
*Pimelodella grisea* (Regan, 1903)							X		Regan (1913)
*Pimelodella linami* Schultz, 1944					X				Galvis et al. (2007a)
*Pimelodella macrocephala* (Miles, 1943)	X	X	X			X			Miles (1943) | MCZ 35876 | USNM 120157
*Pimelodella metae* Eigenmann, 1917					X				Eigenmann (1917b) | CAS [ex IU 13768] | FMNH 58441 [ex CM 7441 or 7141], 58442 | ICN-MHN 17128
*Pimelodella modestus* (Günther, 1860)							X		Eigenmann (1922)
*Pimelodella odynea* Schultz, 1944								X	Ortega-Lara et al. (2012)
*Pimelodella pallida* Dahl, 1961	X	X			X				Dahl (1961)
*Pimelodella reyesi* Dahl, 1964	X	X						X	Dahl and Medem (1964)
*Rhamdia guatemalensis (Günther, 1864)						X	X	X	Hernández et al. (2015)
*Rhamdia laukidi* Bleeker, 1858					X				Hernández et al. (2015)
*Rhamdia quelen* (Quoy & Gaymard, 1824)				X	X	-	-	-	Bogotá-Gregory and Maldonado-Ocampo (2006a) | Eigenmann (1922) | IAvH-P 6182, 6223 | ICN-MHN 5029, 6579, 6591
*Rhamdia saijaensis Rendahl, 1941	X	X					X		Rendahl (1941)
**Pimelodidae53 spp (3 added and 4 deleted from 2008)**									
*Aguarunichthys inpai* Zuanon, Rapp Py-Daniel & Jégu, 1993				X					Mojica et al. (2005)
*Brachyplatystoma filamentosum* (Lichtenstein, 1819)			X	X	X				Ajiaco-Martínez et al. 2012a)
*Brachyplatystoma juruense* (Boulenger, 1898)			X	X	X				Ramírez-Gil et al. (2012a) | Cala (1977)
*Brachyplatystoma platynemum* Boulenger, 1898			X	X	X				Ramírez-Gil et al. (2012b)
*Brachyplatystoma rousseauxii* (Castelnau, 1855)			X	X	X				Agudelo-Córdoba et al. (2012)
*Brachyplatystoma tigrinum* (Britski, 1981)				X					Mojica et al. (2005)
*Brachyplatystoma vaillantii* (Valenciennes, 1840)			X	X	X				Ajiaco-Martínez (2012b) | Dahl (1961)
*Platynematichthys notatus* (Jardine, 1841)				X	X				Mojica et al. (2005) | Maldonado-Ocampo et al. (2004)
*Calophysus macropterus* (Lichtenstein, 1819)				X	X				Dahl (1961) | Mojica et al. (2005) | IAvH-P 2210 | ICN-MHN 7889, 8158
*Cheirocerus abuelo* (Schultz, 1944)								X	Ortega-Lara et al. (2012)
*Cheirocerus goeldii* (Steindachner, 1908)				X					Mojica et al. (2005)
*Duopalatinus peruanus* Eigenmann & Allen, 1942					X				MPUJ
*Exallodontus aguanai Lundberg, Mago-Leccia & Nass, 1991					X				CZUT-IC 9407, 9420, 9553, 9660, 9708, 9727, 9733, 9745, 9842
*Hemisorubim platyrhynchos* (Valenciennes, 1840)				X	X				Maldonado-Ocampo et al. (2006a) | Mojica et al. (2005)
*Hypophthalmus edentatus* Spix & Agassiz, 1829				X	X				Mojica et al. (2005) | Galvis et al. (2007a)
*Hypophthalmus fimbriatus* Kner, 1858				X					Mojica et al. (2005)
*Hypophthalmus marginatus* Valenciennes, 1840				X	X				Mojica et al. (2005) | Lasso et al. (2005) | ICN-MHN 8228
*Hypophthalmus oremaculatus* Nani & Fuster, 1947				X	+				Mojica et al. (2005) | Littmann et al. (2015)
*Leiarius marmoratus* (Gill, 1870)				X	X				Mojica et al. (2005) | Eigenmann (1922)
*Leiarius perruno* (Schultz, 1944)				-				+	Ortega-Lara et al. (2012)
*Leiarius pictus* (Müller & Troschel, 1849)				X	X				Lasso et al. (2005) | Correa (2003) | IMCN 842
*Megalonema orixanthum* Lunberg & Dahdul, 2008					X				Lundberg and Dahdul (2008) | ANSP 187449 (ex ANSP 148143)
*Megalonema platycephalum* Eigenmann, 1912				-	X				Dahl (1961)}
*Megalonema psammium* Schultz, 1944								X	Ortega-Lara et al. (2012)
*Megalonema xanthum* Eigenmann, 1912	X	X	X			X			Eigenmann (1912) | FMNH 56032 [ex CM 4822] | AMNH 5340 | BMNH 1920.12.20.112-113, 1924.3.3.83-84 | CAS 63674-75 [ex IU 12681-82] | FMNH 10251, 10284-89, 77909, 56032-33, 69818, 95984 | MCZ 30961 | UMMZ 190406 | USNM 76930, 79222, 167852
*Phractocephalus hemioliopterus* (Bloch & Schneider, 1801)				X	X				Mojica et al. (2005) | Dahl (1961)
*Pimelodina flavipinnis* Steindachner, 1877				X	X				Lasso et al. (2005) | Mojica et al. (2005) | ICN-MHN 6874, 8143
*Pimelodus albofasciatus* Mees, 1974				+	X				Lasso et al. (2005) | IAvH-P 256, 503, 836, 477, 2150, 2677, 2731, 6023, 8754, 8821, 8850, 8913 | ICN-MHN 1259, 1951, 1798, 3725, 3726 | 3822, 3386
*Pimelodus blochii* Valenciennes, 1840				X	X	X	X	X	Mojica et al. (2005) | Galvis et al. (2007a) | Mojica et al. (2006a) | Maldonado-Ocampo et al. (2013b)
*Pimelodus coprophagus* Schultz, 1944			X					X	Ortega-Lara et al. (2012)
*Pimelodus crypticus* Villa-Navarro & Cala, 2017	X	X				X			Villa-Navarro et al. (2017)
*Pimelodus garciabarrigai* Dahl, 1960	X	X			X				Dahl (1961) | ICN-MHN 744, ICN-MHN 410
*Pimelodus grosskopfii* Steindachner, 1879	X	X	X			X	-	-	Steindachner (1879b) | NMW 45781-45782
*Pimelodus navarroi* Schultz, 1944								X	Mojica (1999) | ICN-MHN 2150
*Pimelodus ornatus* Kner, 1858				X	X				Mojica et al. (2005) | Dahl (1961)
*Pimelodus pictus* Steindachner, 1877				X	X				Mojica et al. (2005) | Galvis et al. (2007a)
*Pimelodus punctatus* (Meek & Hildebrand, 1913)							+	X	Maldonado-Ocampo et al. (2013b) | Villa-Navarro et al. (2017)
*Pimelodus yuma* Villa-Navarro & Acero P., 2017	X	X				X		X	Villa-Navarro et al. (2017)
*Pinirampus pirinampu* (Spix & Agassiz, 1829)				X	X				Mojica et al. (2005) | Maldonado-Ocampo et al. (2006a)
*Platysilurus malarmo* Schultz, 1944			X					X	Ortega-Lara et al. (2012)
*Platysilurus mucosus* (Vaillant, 1880)				X	X				Mojica et al. (2005) | Galvis et al. (2007a)
*Platystomatichthys sturio* (Kner, 1858)				X					Mojica et al. (2005)
*Pseudoplatystoma magdaleniatum* Buitrago-Suárez & Burr, 2007	X	X	X			X			Buitrago-Suárez and Burr (2007) | CAS 19165 | FMNH 56278, 59234
*Pseudoplatystoma metaense* Buitrago-Suárez & Burr, 2007			X		X				Buitrago-Suárez and Burr (2007) | ANSP 146858, 128135, 149541
*Pseudoplatystoma orinocoense* Buitrago-Suárez & Burr, 2007			X		X				Buitrago-Suárez and Burr (2007)
*Pseudoplatystoma punctifer* (Castelnau, 1855)			X	X					Buitrago-Suárez and Burr (2007)
*Pseudoplatystoma tigrinum* (Valenciennes, 1840)			X	X					Buitrago-Suárez and Burr (2007)
*Sorubim cuspicaudus* Littmann, Burr & Nass, 2000			X			X		+	Littmann et al. (2000) | AUM 28756 | CAS 150404, 150406 | EBRG 8216 | FMNH 56223, 60305, 107492 | IAvH-P 11809 | INHS 35428;
*Sorubim elongatus* Littmann, Burr, Schmidt & Isern, 2001				X	X				Littmann et al. (2001)
*Sorubim lima* (Bloch & Schneider, 1801)			X	X	X				Littmann (2007) | Dahl (1961)
*Sorubim maniradii* Littmann, Burr & Buitrago-Suarez, 2001				X					Mojica et al. (2005)
*Sorubimichthys planiceps* (Spix & Agassiz, 1829)			X	X	X				Mojica et al. (2005) | Dahl (1961) | ICN-MHN 7880, 9938, 17351
*Zungaro zungaro* (Humboldt, 1821)			X	X	X				Mojica et al. (2005) |
**Pseudopimelodidae12 spp (1 added from 2008)**									
*Batrochoglanis acanthochiroides* (Günther, 1942)							-	+	Ortega-Lara et al. (2012)
*Batrochoglanis raninus* (Valenciennes, 1840)				X	-				Bogotá-Gregory and Maldonado-Ocampo (2005)
*Batrochoglanis transmontanus* (Regan, 1913)						-	X	X	Regan (1913) | Maldonado-Ocampo et al. (2013b)
*Batrochoglanis villosus* (Eigenmann, 1912)				-	+				Lasso et al. 2004, Bogotá-Gregory and Maldonado-Ocampo 2006, Maldonado-Ocampo et al. 2008, Lasso et al. 2009 | IAvH-P 5705, 10059, 10060, 10061, 10062, 10063, 10064, 10065, 10066, 10743, 10798, 12466, 12467, 12481, 12867, 12880, 12913
*Cephalosilurus apurensis* (Mees, 1978)					X				Lasso et al. (2005) | IAvH-P 3731
*Cruciglanis pacifici* Ortega-Lara & Lehmann, 2006	X	X	X				X		Ortega-Lara and Lehmann (2006) | IMCN 2359 | IAvH-P 7505
*Microglanis iheringi* Gomes, 1946					X				Cala (1977)
*Microglanis poecilus* Eigenmann, 1912				X	X				Mojica et al. (2005) | Maldonado-Ocampo et al. (2006a)
*Microglanis secundus* Mees, 1974					X			-	Lasso et al. (2005) | ICN-MHN 1912
*Pseudopimelodus bufonius* (Valenciennes, 1840)				X	X	-		X	Lasso et al. (2005) | Ortega-Lara et al. (2012) | IAvH-P 3371, 9198-9199, 11294, 12291 | ICN-MHN 772, 6582
*Pseudopimelodus schultzi* (Dahl, 1955)	X	X	X		-	X		X	Dahl (1955) | Mojica et al. (2006a)
*Rhyacoglanis annulatus* Shibatta & Vari, 2017					X				Shibatta and Vari (2017)
**Batrachoidiformes3 spp**									
**Batrachoididae**									
*Daector gerringi* (Rendahl, 1941)	X	X						X	Rendahl (1941) | NRM 10651
*Daector quadrizonatus* (Eigenmann, 1922)	X	X					-	X	Eigenmann (1922) | FMNH [ex CM 3921]
*Thalassophryne amazonica* Steindachner, 1876				X					Mojica et al. (2005)
**Gobiiformes1 sp (1 deleted from 2008)**									
**Eleotridae**									
*Microphilypnus ternetzi* Myers, 1927				+	X				Caires and Figueiredo (2011) | Galvis et al. (2007a)
**Synbranchiformes1 sp**									
**Synbranchidae**									
*Synbranchus marmoratus* Bloch, 1795				X	X	X	X	X	Mojica et al. (2005) | Fowler (1943) | Galvis et al. (2007a) | Steindachner (1880) | Maldonado-Ocampo et al. (2013b) | Regan (1913)
**Pleuronectiformes5 spp (2 added and 2 deleted from 2008)**									
**Achiridae**									
*Achirus novoae* Cervigón, 1982					X				Maldonado-Ocampo et al. (2006a)
*Apionichthys nattereri (Steindachner, 1876)				X					Mojica et al. (2005)
*Apionichthys sauli* Ramos, 2003					X				Ramos (2003b) | ANSP 176081, 163851-53,163854, 163855-56 | MZUSP 52055 | UFPB 3596
*Hypoclinemus mentalis* (Günther, 1862)				X	X				Mojica et al. (2005) | Lasso et al. (2009) | IMCN 2657, 2662 | IAvH-P 10784-10787
*Trinectes hubbsbollinger* Duplain, Chapleau & Munroe, 2012	X	X					X		Duplain et al. (2012) | BMNH 1915.10.1.19,
**Ovalentaria *incertae sedis***									
**Polycentridae1 sp**									
*Monocirrhus polyacanthus* Heckel, 1840				X	X				Arbeláez et al. (2004) | Cala (1977)
**Cichliformes94 spp (8 added and 30 deleted from 2008)**									
**Cichlidae**									
*Cichla intermedia* Machado-Allison, 1971					X				Maldonado-Ocampo (2001)
*Cichla monoculus* Spix & Agassiz, 1831				X	X				Mojica et al. (2005) | Maldonado-Ocampo et al. (2006a)
*Cichla orinocensis* Humboldt, 1821				X	X				Kullander and Ferreira (2006)
*Cichla temensis* Humboldt, 1821				X	X				Galvis et al. (2007b) | Cala (1977)
*Chaetobranchus flavescens* Heckel, 1840				X	X				Kullander (1986) | Galvis et al. (2007a)
*Acarichthys heckelii* (Müller & Troschel, 1849)				X					Kullander (1986)
*Biotoecus dicentrarchus* Kullander, 1989					X				Kullander (1989a) | ICN-MHN 1400 | FMNH 85650, 105089 | NRM 16662, 37078
*Crenicara punctulatum* (Günther, 1863)				X					Mojica et al. (2005)
*Crenicichla alta* Eigenmann, 1912				X	X				Bogotá-Gregory and Maldonado-Ocampo (2005) | Maldonado-Ocampo et al. (2006a)
*Crenicichla anthurus* Cope, 1872				X	X				Correa (2003) | Galvis et al. (2007a)
*Crenicichla geayi* Pellegrin, 1903					X				Regan (1905)
*Crenicichla johanna* Heckel, 1840				X	X				Mojica et al. (2005)
*Crenicichla lenticulata* Heckel, 1840				X	X				Correa (2003) | Cala (1977)
*Crenicichla lugubris* Heckel, 1840				X	X				Fowler (1943) | IAvH-P 389, 2327, 4893, 10110 | ICN-MHN 867, 877, 884, 1553, 1668, 2286
*Crenicichla monicae* Kullander & Varella, 2015				X					Kullander and Varella (2015)
*Crenicichla reticulata* (Heckel, 1840)				X	-				Kullander (1986)
*Crenicichla strigata* Günther, 1862				X					Bogotá-Gregory and Maldonado-Ocampo (2006a) | ICN-MHN 2594, 10233
*Crenicichla sveni* Ploeg, 1991					X				Ploeg (1991) | RMNH 31622, 31623 | ZMA 120388
*Crenicichla zebrina Montaña, López-Fernández & Taphorn 2008					X				IMCN 6476
*Dicrossus filamentosus* (Ladiges, 1958)					X				Cala (1977)
*Dicrossus gladicauda* Schindler & Staeck, 2008					X				Schindler and Staeck (2008) | MTDF 31312, 33313-18
*Apistogramma agassizii* (Steindachner, 1875)				X					Kullander (1986)
*Apistogramma alacrina* Kullander, 2004	X	X		X	X				Kullander (2004) | UF 33670, 128785, 33687, 33717 | CAS 50638 | ICNMNHN 6735 | NRM 27040, 33375, 36102
*Apistogramma bitaeniata* Pellegrin, 1936				X					Kullander (1986)
*Apistogramma cacatuoides* Hoedeman, 1951				X					Kullander (1986)
*Apistogramma cruzi* Kullander, 1986				X					Kullander (1986)
*Apistogramma diplotaenia* Kullander, 1987				X					Bogotá-Gregory and Maldonado-Ocampo (2005)
*Apistogramma eunotus* Kullander, 1981				X					Kullander (1986) | NRM 17784
*Apistogramma flabellicauda Mesa S. & Lasso, 2011				X	X				CZUT-IC 1494, IAvH-P 10087, 10089-10092, 10094-10098
*Apistogramma hoignei* Meinken, 1965					X				Urbano-Bonilla et al. (2009).
*Apistogramma hongsloi* Kullander, 1979				-	X				Kullander (1979) | NRM 11234, 11235-39
*Apistogramma iniridae* Kullander, 1979	X	X		X	X				Kullander (1979) | Bogotá-Gregory and Maldonado-Ocampo (2005)
*Apistogramma lineata* Mesa S. & Lasso, 2011	X	X			X				Mesa S and Lasso (2011b)
*Apistogramma macmasteri* Kullander, 1979	X	X			X				Kullander (1979) | AMNH 22733 | FMNH 55218 [ex CM 3930] | ICN-MHN 17117 | NRM 11240, 11241-44
*Apistogramma megaptera* Mesa & Lasso, 2011					X				Mesa Salazar and Lasso (2011a)
*Apistogramma minima* Mesa S. & Lasso, 2011					X				Mesa S and Lasso (2011b) | IAvH-P 11876
*Apistogramma piaroa* Mesa S. & Lasso, 2011					X				Mesa S and Lasso (2011b)
*Apistogramma velifera* Staeck, 2003					X				Mesa S and Lasso (2011b)
*Apistogramma viejita* Kullander, 1979					X				Kullander (1979) | NRM 11231, 11232
*Apistogrammoides pucallpaensis* Meinken, 1965				X					Kullander (1986)
*Biotodoma cupido* (Heckel, 1840)				X					Mojica et al. (2005)
*Biotodoma wavrini* (Gosse, 1963)				X	X				Maldonado-Ocampo et al. (2006a) | Arbeláez et al. (2004)
*Geophagus abalios* López-Fernández & Taphorn, 2004				X	X				Bogotá-Gregory and Maldonado-Ocampo (2005) | Galvis et al. (2007a)
*Geophagus crassilabris* Steindachner, 1876							X		ICN-MHN 98
*Geophagus dicrozoster* López-Fernández & Taphorn, 2004					X				Maldonado-Ocampo et al. (2006a)
*Geophagus megasema* Heckel, 1840				X					Bogotá-Gregory and Maldonado-Ocampo (2005)
*Geophagus pellegrini* Regan, 1912	X	X					X	X	Regan (1912a) | Eigenmann (1922)
*Geophagus steindachneri* Eigenmann & Hildebrand, 1910						X		X	Eigenmann (1910) | Ortega-Lara et al. (2012)
*Geophagus surinamensis* (Bloch, 1791)				X					Correa (2003)
*Geophagus taeniopareius* Kullander & Royero, 1992					X				Maldonado-Ocampo et al. (2006a)
*Geophagus winemilleri* López-Fernández & Taphorn, 2004				X	X				Bogotá-Gregory and Maldonado-Ocampo (2005) | Maldonado-Ocampo and Bogotá-Gregory (2007)
*Mikrogeophagus ramirezi* (Myers & Harry, 1948)					X				Cala (1977)
*Satanoperca daemon* (Heckel, 1840)				X	X				Correa (2003) | Maldonado-Ocampo et al. (2006a)
*Satanoperca jurupari* (Heckel, 1840)				X					Kullander (1986)
*Satanoperca mapiritensis* (Fernández-Yépez, 1950)					X				Galvis et al. (2007a)
*Astronotus ocellatus* (Agassiz, 1831)				X	X				Mojica et al. (2005) | Galvis et al. (2007a)
*Acaronia nassa* (Heckel, 1840)				X					Bogotá-Gregory and Maldonado-Ocampo (2006a) | IAvH-P 5308
*Acaronia vultuosa* Kullander, 1989				-	X				Kullander (1989b)
*Aequidens diadema* (Heckel, 1840)				X	X				Arbeláez et al. (2004) | Maldonado-Ocampo et al. (2006a)
*Aequidens metae* Eigenmann, 1922				-	X				Eigenmann (1922) | CAS 66884 [ex IU 13967], 66983 [ex IU 13967] | IAvH-P 3162
*Aequidens tetramerus* (Heckel, 1840)				X	X				Mojica et al. (2005)
*Andinoacara biseriatus* (Regan, 1913)	X	X					X	X	Regan (1913) | Eigenmann (1922)
*Andinoacara latifrons* (Steindachner, 1878)	X	X				X	X	X	Steindachner (1878) | Maldonado-Ocampo et al. (2013b) | Eigenmann (1922)
*Andinoacara sapayensis* (Regan, 1903)							X		Eigenmann (1922)
*Bujurquina mariae* (Eigenmann, 1922)	X	X		-	X				Eigenmann (1922)
*Bujurquina moriorum* Kullander, 1986				X					Kullander (1986)
*Bujurquina peregrinabunda* Kullander, 1986				X					Kullander (1986)
*Bujurquina syspilus* (Cope, 1872)				X					Mojica (1999) | ICN-MHN 1523, 1529
*Chocoheros microlepis* (Dahl, 1960)	X	X					X		Dahl (1960c) | ICN-MHN 95, 95a
*Cichlasoma amazonarum* Kullander, 1983				X					Kullander (1986)
*Cichlasoma gephyrum* Eigenmann, 1922	X	X					X		Eigenmann (1922) | FMNH 58614 [ex CM 7639], 58615 [ex CM 7639] | CAS 66956 [ex IU 14171]
*Cichlasoma orinocense* Kullander, 1983					X				Kullander (1983) | ANSP 127364, 127364, 127397 | FMNH 84008 | NRM 39282
*Laetacara flavilabris* (Cope, 1870)				X					IMCN 6026
*Laetacara fulvipinnis Staeck & Schindler, 2007					X				CZUT-IC 8653
*Laetacara thayeri* (Steindachner, 1875)				X					Mojica et al. (2005)
*Caquetaia kraussii* (Steindachner, 1879)						X	-	X	Steindachner (1878) | Eigenmann (1922)
*Caquetaia myersi* (Schultz, 1944)				X					Schultz (1944c) | USNM 120533, 120534
*Heroina isonycterina* Kullander, 1996				X					Kullander (1996)
*Heros efasciatus* Heckel, 1840				X					Kullander (1986)
*Heros severus* Heckel, 1840				X	X				Correa (2003) | Cala (1977)
*Hoplarchus psittacus* (Heckel, 1840)				X	X				Bogotá-Gregory and Maldonado-Ocampo (2006a) | Maldonado-Ocampo et al. (2006a) | IAvH-P 2224
*Hypselecara coryphaenoides* (Heckel, 1840)				X	X				Arbeláez et al. (2004) | Galvis et al. (2007a)
*Hypselecara temporalis* (Günther, 1862)				X					Kullander (1986)
*Kronoheros umbrifer* (Meek & Hildebrand, 1913)			X			X		X	Eigenmann (1922)
*Mesoheros atromaculatus* (Regan, 1912)	X	X					X	X	Regan (1912a) | Eigenmann (1922)
*Mesoheros ornatus* (Regan, 1905)							X	+	Fowler (1944) | Eigenmann (1922)
*Mesonauta egregius* Kullander & Silfvergrip, 1991					X				Kullander and Silfvergrip (1991) | FMNH 92863 | ICNMNH 1686 [ex NRM 43043] | NRM 11302, 12280
*Mesonauta insignis* (Heckel, 1840)				X	X				Kullander (1986) | Kullander and Silfvergrip (1991)
*Mesonauta mirificus* Kullander & Silfvergrip, 1991				X					Kullander and Silfvergrip (1991)
*Pterophyllum altum* Pellegrin, 1903			X		X				Cala (1977)
*Pterophyllum scalare* (Schultze, 1823)				X					Arbeláez et al. (2004)
*Symphysodon aequifasciatus* Pellegrin, 1904				X					Correa (2003)
*Uaru amphiacanthoides* Heckel, 1840				X					Correa (2003)
*Uaru fernandezyepezi* Stawikowski, 1989					X				Galvis et al. (2007a)
**Beloniformes4 spp (1 deleted from 2008)**									
**Belonidae**									
*Belonion dibranchodon* Collette, 1966				X	X				Correa (2003) | Maldonado-Ocampo (2001)
*Potamorrhaphis guianensis* (Jardine, 1843)				X	X				Galvis et al. (2007b); Cala (1977)
*Potamorrhaphis petersi* Collette, 1974					X				Galvis et al. (2007a)
*Pseudotylosurus microps* (Günther, 1866)				X	X				Mojica et al. (2005) | Maldonado-Ocampo (2001)
**Cyprinodontiformes42 spp (7 added and 3 deleted from 2008)**									
**24 spp (5 added and 2 deleted from 2008)**									
*Anablepsoides atratus* (Garman, 1895)				X					NRM 14686
*Anablepsoides elongatus* (Fels & de Rham, 1981)				X					Correa (2003)
*Anablepsoides ophiomimus* (Huber, 1992)				X					Correa (2003)
*Anablepsoides ornatus* (Garman, 1895)				X					Mojica et al. (2005)
*Anablepsoides rubrolineatus* (Fels & de Rham, 1981)				X					Mojica et al. (2005)
*Anablepsoides taeniatus* (Fowler, 1945)	X	X		X					Fowler (1945) | ANSP 71720,71721
*Anablepsoides tessellatus* (Huber, 1992)	X	X			X				Huber (1992) | ANSP 139468, 168979
*Austrofundulus guajira* Hrbek, Taphorn & Thomerson, 2005			X					X	Hrbek et al. (2005)
*Austrofundulus myersi* Dahl, 1958	X	X				X			Dahl (1958) | SU 49513, 49513, 68324 | DZVU [ex SU 49513]
*Cynodonichthys boehlkei* (Huber & Fels, 1985)	X	X				X			Huber and Fels (1985) | ANSP 139467, 139467
*Cynodonichthys elegans* (Steindachner, 1880)	X	X			-	X	-	-	Steindachner (1880) | NMW 605441, 605442-3, 18893-6
*Cynodonichthys leucurus* (Fowler, 1944)	X	X					X		Fowler (1944) | ANSP 71436, 71437-50
*Cynodonichthys magdalenae* (Eigenmann & Henn, 1916)	X	X				X			Henn (1916) | CAS 44227 [ex CM 5814a-m | IU 13603], 52234 [ex IU 13606], 52235 [ex IU 13605], 78377 [ex IU 13606] | FMNH 56997 [ex CM 5813], 56998-99, 57000-02
*Cynodonichthys pacificus* (Huber, 1992)	X	X					X	X	Huber (1992) | Maldonado-Ocampo et al. (2013b)
*Laimosemion altivelis* (Huber, 1992)	X	X			X				Huber (1992) | NRM 16548, 14687
*Laimosemion corpulentus* (Thomerson & Taphorn, 1993)	X	X			X				Thomerson and Taphorn (1993) | SU [not CAS] 69692, 53693
*Laimosemion leticia* Valdesalici, 2016	X	X		X					Valdesalici (2016)
*Rachovia brevis* (Regan, 1912)						X		X	Regan (1912d) | Mojica et al. (2006b)
*Rachovia hummelincki* de Beaufort, 1940						X			Taphorn and Thomerson (1978)
*Rachovia maculipinnis* (Radda, 1964)					X				Taphorn and Thomerson (1978)
*Rivulus azurescens* Vermeulen, 2013	X	X				X			Vermeulen (2013)
*Rivulus pivijay* Vermeulen, 2013	X	X				X			Vermeulen (2013)
*Rivulus ribesrubrum* Vermeulen, 2013	X	X				X			Vermeulen (2013)
*Rivulus xi* Vermeulen, 2013	X	X				X			Vermeulen (2013)
**Cyprinodontidae1 sp**									
**Yssolebias martae (Steindachner, 1876)	X	X						X	Steindachner (1876)
**Poeciliidae17 spp (2 added and 1 deleted from 2008)**									
*Fluviphylax obscurus* Costa, 1996					X				IAvH-P 2171, 2219
*Fluviphylax pygmaeus* (Myers & Carvalho, 1955)				X	+				Lucinda (2003) | NRM 26248
*Gambusia lemaitrei* Fowler, 1950	X	X				-		+	Fowler (1950) | ANSP 71938, 71939-40
*Neoheterandria elegans* Henn, 1916	X	X						X	Henn (1916) | FMNH 57007 [ex CM 5823], 57008 [ex CM 5824a-g] | AMNH 22705sw [ex FMNH 57008], 36481 [ex IU 13612] | CAS 22768 [ex IU 13612] | UMMZ 65232 [ex IU 13612]
*Poeciliopsis turrubarensis* (Meek, 1912)							X		Rosen and Bailey (1963)
*Priapichthys caliensis* (Eigenmann & Henn, 1916)	X	X				X	-		Henn (1916) | FMNH 57721 [ex CM 6700a], FMNH [ex CM 6700b]
*Priapichthys chocoensis* (Henn, 1916)	X	X					X		Henn (1916) | CAS 22547 [ex IU 13618], 22560 [ex IU 13619] | FMNH 57009 | UMMZ 65223 [ex IU 13619]
*Priapichthys darienensis (Meek & Hildebrand, 1913)								X	CZUT-IC 11742
*Priapichthys nigroventralis* (Eigenmann & Henn, 1912)	X	X					X	X	Eigenmann (1912) | Henn (1916)
*Pseudopoecilia austrocolumbiana* Radda, 1987	X	X					X		Radda (1987) | FMNH 56045 [ex CM 4835], 54046 | BMNH 1924.3.3.85-86 | CAS 22765 [ex IU 12689] | USNM 79205
*Pseudopoecilia fria* (Eigenmann & Henn, 1914)							X		Maldonado-Ocampo et al. (2013b) | ICN-MHN 2335
*Poecilia caucana* (Steindachner, 1880)						X		X	Steindachner (1880) | Mojica et al. (2006b)
*Poecilia gillii (Kner, 1863)								X	Poeser (2003a)
**Poecilia koperi Poeser, 2003								X	Poeser (2003b)
*Poecilia mechthildae* Meyer, Etzel & Bork, 2002	X	X						X	Meyer et al. (2002) | MTDF 26473-26476 | SMF 28889
*Poecilia orri* Fowler, 1943								X	Poeser (2003a)
*Poecilia sphenops* Valenciennes, 1846								X	Agudelo-Zamora et al. (2010)
**Eupercaria *incertae sedis* 7 spp**									
**Sciaenidae**									
*Pachypops fourcroi* (Lacepède, 1802)				X	+				Casatti (2002) | IAvH-P
*Pachypops trifilis* (Müller & Troschel, 1849)				X					Bogotá-Gregory and Maldonado-Ocampo (2005)
*Pachyurus gabrielensis* Casatti, 2001					X				Maldonado-Ocampo et al. (2008)
*Pachyurus junki* Soares & Casatti, 2000				X					Bogotá-Gregory and Maldonado-Ocampo (2006a)
*Pachyurus schomburgkii* Günther, 1860				X	X				Mojica et al. (2005) | Galvis et al. (2007a)
*Plagioscion magdalenae* (Steindachner, 1878)			X			X		X	Steindachner (1878) | Mojica et al. (2006b)
*Plagioscion squamosissimus* (Heckel, 1840)				X	X				Mojica et al. (2005) | Cala (1977) | Casatti (2005)
**Tetraodontiformes**									
**Tetraodontidae**									
*Colomesus asellus* (Müller & Troschel, 1849)				X					Mojica et al. (2005)
**Ceratodontiformes**									
**Lepidosirenidae**									
*Lepidosiren paradoxa* Fitzinger, 1837			X	X	X				Mojica et al. (2005) | Bogotá-Gregory and Maldonado-Ocampo (2006b)
						
**Total of additions by hydrographic system**				11	23	3	3	18	
**Total of deletions by hidrographic system**				44	32	37	24	19	
**Total number of modifications by hidrographic system**				55	55	40	27	37	

### Final remarks

Confusions and misinterpretations related to the freshwater fishes distributed in Colombian territory and erroneous name assignations were included in previous checklists at local, regional or national levels, as pointed out in previous sections. The exhaustive verification process of the current checklist of the freshwater fishes of Colombia is a step forward linked to the ichthyology collections database depuration process in progress. These efforts will bring new opportunities to insert this information into new analysis dealing with new conservation opportunities or approaches to counteract the drivers of biodiversity decline of the Colombian freshwater fish fauna.

New protected areas or portfolios have been proposed for freshwaters ecosystems in the Orinoco, Magdalena-Cauca and Amazon River basins in the recent years (Lasso et al. 2011b; Téllez et al. 2011; Portocarrero-Aya and Cowx 2016). However, some species level information taken into account to designate those areas were wrong; criteria like species richness or endemic species have to be re-evaluated based on the information provided in the current checklist. Expert criteria, although important on those analysis and proposals, also have to be reevaluated in light of rigorous data depuration at taxonomic and geographic levels (e.g. Herrera 2015). Applying a decision theory approach to guide and improve the process in the field of conservation planning is important to deal with an incomplete geographic coverage of taxonomic groups (Ornellas et al. 2011), as is the case of the Colombian freshwater fishes.

As it was mentioned in the introduction, new scenarios are in discussion in reference to new approaches dealing with the conservation of the freshwater ecosystems and their biota worldwide (Ormerod 2014). Colombia as a country with one of the richest freshwater fish faunas, faces enormous challenges due to the past and current human impacts that have been affecting the river systems running through the Colombian landscape. Most remarkable is that an important number of species have small distribution ranges in many trans-Andean rivers and piedmont areas of the Orinoco and Amazon regions.

Finally, the data depuration process, conducted mainly at the species level, reinforces the importance of biological collections as repositories of the biodiversity patrimony and allows specimen identifications to be rechecked and verified. More efforts are needed by the institutions in charge of the Colombian ichthyological collections to guarantee the adequate conservation of their specimens. A continuous interinstitutional effort and collection support is an important task to be facilitated by ACICTIOS and the Colombian Biodiversity Information System (SiB Colombia). Those interactions have an enormous impact in terms of the need to maintain a continuous and rigorous updating list process. Moving forward (in addition to field work to fill gaps in poorly sampled basins), we identify as the main tasks remaining: 1) to continue the process of verification of fish species distributions at the sub-basin level or smaller biogeographic areas; and 2) to conduct revisionary taxonomic studies of specific species-rich fish groups found in Colombia. Ideally, these studies have to be developed in the context of an integrative taxonomic approach.

## Supplementary materials

Supplementary material 1 Checklist of the native freshwater fishes of Colombia Data type: Microsoft Excel Worksheet (.xlsx) This dataset is made available under the Open Database License (http://opendatacommons.org/licenses/odbl/1.0/). The Open Database License (ODbL) is a license agreement intended to allow users to freely share, modify, and use this Dataset while maintaining this same freedom for others, provided that the original source and author(s) are credited.Carlos DoNascimiento, Edgar Esteban Herrera-Collazos, Guido A. Herrera-R, Armando Ortega-Lara, Francisco A. Villa-Navarro, José Saulo Usma Oviedo, Javier A. Maldonado-Ocampo

Supplementary material 2 New species described from Colombia after Maldonado-Ocampo et al. (2008) Data type: Microsoft Word Document (.docx) This dataset is made available under the Open Database License (http://opendatacommons.org/licenses/odbl/1.0/). The Open Database License (ODbL) is a license agreement intended to allow users to freely share, modify, and use this Dataset while maintaining this same freedom for others, provided that the original source and author(s) are credited.Carlos DoNascimiento, Edgar Esteban Herrera-Collazos, Guido A. Herrera-R, Armando Ortega-Lara, Francisco A. Villa-Navarro, José Saulo Usma Oviedo, Javier A. Maldonado-Ocampo

Supplementary material 3 New records of species for Colombia after Maldonado-Ocampo et al. (2008) Data type: Microsoft Word Document (.docx) This dataset is made available under the Open Database License (http://opendatacommons.org/licenses/odbl/1.0/). The Open Database License (ODbL) is a license agreement intended to allow users to freely share, modify, and use this Dataset while maintaining this same freedom for others, provided that the original source and author(s) are credited.Carlos DoNascimiento, Edgar Esteban Herrera-Collazos, Guido A. Herrera-R, Armando Ortega-Lara, Francisco A. Villa-Navarro, José Saulo Usma Oviedo, Javier A. Maldonado-Ocampo

Supplementary material 4 Species listed in Maldonado-Ocampo et al. (2008) with geographic distribution corrected Data type: Microsoft Word Document (.docx) This dataset is made available under the Open Database License (http://opendatacommons.org/licenses/odbl/1.0/). The Open Database License (ODbL) is a license agreement intended to allow users to freely share, modify, and use this Dataset while maintaining this same freedom for others, provided that the original source and author(s) are credited.Carlos DoNascimiento, Edgar Esteban Herrera-Collazos, Guido A. Herrera-R, Armando Ortega-Lara, Francisco A. Villa-Navarro, José Saulo Usma Oviedo, Javier A. Maldonado-Ocampo

Supplementary material 5 Species deleted from the Colombian continental ichthyofauna Data type: Microsoft Word Document (.docx) This dataset is made available under the Open Database License (http://opendatacommons.org/licenses/odbl/1.0/). The Open Database License (ODbL) is a license agreement intended to allow users to freely share, modify, and use this Dataset while maintaining this same freedom for others, provided that the original source and author(s) are credited.Carlos DoNascimiento, Edgar Esteban Herrera-Collazos, Guido A. Herrera-R, Armando Ortega-Lara, Francisco A. Villa-Navarro, José Saulo Usma Oviedo, Javier A. Maldonado-Ocampo
