# Supplementary material for: Checklist of the freshwater fishes of Colombia: a Darwin Core alternative to the updating problem
Source: Zookeys. 2017 Oct 13;(708):25–138. doi: 10.3897/zookeys.708.13897 (PMC5674168; doi:10.3897/zookeys.708.13897)
Supplement: Supplementary material 2 — New species described from Colombia after Maldonado-Ocampo et al. (2008) [file zookeys-708-025-s002.docx]

**SUPPLEMENTARY FILE 2**

New species described from Colombia after Maldonado-Ocampo *et al*. (2008). Numbers in parenthesis following each taxon name indicate the number of new species added to each taxon. Species are listed within their respective family by year of description and alphabetically by genus and species names. The three species indicated by an asterisk were inadvertently omitted from the above mentioned reference and consequently are listed here at the end of the table.

| **Taxa** | **DC** | **EN** | **Amz** | **Ori** | **Mag-Cauc** | **Pac** | **Car** | **Collections/References** |
| --- | --- | --- | --- | --- | --- | --- | --- | --- |
| **Characiformes (39)** |  |  |  |  |  |  |  |  |
| **Parodontidae (3)** |  |  |  |  |  |  |  |  |
| *Parodon alfonsoi* Londoño-Burbano, Román-Valencia & Taphorn, 2011 | X | X |  |  | X |  |  | Londoño-Burbano et al. (2011) |
| *Parodon atratoensis* Londoño-Burbano, Román-Valencia & Taphorn, 2011 | X | X |  |  |  |  | X | Londoño-Burbano et al. (2011) |
| *Parodon magdalenensis* Londoño-Burbano, Román-Valencia & Taphorn, 2011 | X | X |  |  | X |  |  | Londoño-Burbano et al. (2011) |
| **Anostomidae (1)** |  |  |  |  |  |  |  |  |
| *Leporinus enyae* Burns, Chatfield, Birindelli & Sidlauskas, 2017 |  |  |  | X |  |  |  | Burns et al. (2017) |
| **Lebiasinidae (1)** |  |  |  |  |  |  |  |  |
| *Lebiasina chocoensis* Ardila Rodríguez, 2010 | X | X |  |  |  |  | X | Ardila Rodríguez (2010) |
| **Characidae (34)** |  |  |  |  |  |  |  |  |
| *Bryconamericus foncensis* Román-Valencia, Vanegas-Ríos & Ruiz-C., 2009 | X | X |  |  | X |  |  | Román-Valencia et al. (2009) |
| *Hemibrycon brevispini* Román-Valencia & Arcila-Mesa, 2009 | X | X |  |  | X |  |  | Román-Valencia and Arcila-Mesa (2009) |
| *Hemibrycon cairoense* Román-Valencia & Arcila-Mesa, 2009 | X | X |  |  | X |  |  | Román-Valencia and Arcila-Mesa (2009) |
| *Bryconamericus macarenae* Román-Valencia, García-Alzate, Ruiz-C. & Taphorn, 2010 | X | X |  | X |  |  |  | Román-Valencia et al. (2010c) |
| *Creagrutus maculosus* Román-Valencia, García-Alzate, Ruiz C. & Taphorn B., 2010 | X | X |  | X |  |  |  | Román-Valencia et al. (2010b) |
| *Hemibrycon paez* Román-Valencia & Arcila-Mesa, 2010 | X | X |  |  | X |  |  | Román-Valencia and Arcila-Mesa (2010) |
| *Hemibrycon palomae* Román-Valencia, García-Alzate, Ruiz-C. & Taphorn, 2010 | X | X |  |  | X |  |  | Román-Valencia et al. (2010a) |
| *Hemibrycon quindos* Román-Valencia & Arcila-Mesa, 2010 | X | X |  |  | X |  |  | Román-Valencia and Arcila-Mesa (2010) |
| *Hemibrycon raqueliae* Román-Valencia & Arcila-Mesa, 2010 | X | X |  |  | X |  |  | Román-Valencia and Arcila-Mesa (2010) |
| *Hemibrycopn santamartae* Román-Valencia, Ruiz-C., García-Alzate & Taphorn, 2010 | X | X |  |  |  |  | X | Román-Valencia et al. (2010d) |
| *Hemibrycon virolinica* Román-Valencia & Arcila-Mesa, 2010 | X | X |  |  | X |  |  | Román-Valencia and Arcila-Mesa (2010) |
| *Hemibrycon yacopiae* Román-Valencia & Arcila-Mesa, 2010 | X | X |  |  | X |  |  | Román-Valencia and Arcila-Mesa (2010) |
| *Hyphessobrycon acaciae* García-Alzate, Román-Valencia & Prada-Pedreros, 2010 | X | X |  | X |  |  |  | García-Alzate et al. (2010a) |
| *Hyphessobrycon amaronensis* García-Alzate, Román-Valencia & Taphorn, 2010 | X | X | X |  |  |  |  | García-Alzate et al. (2010b) |
| *Hyphessobrycon mavro* García-Alzate, Román-Valencia & Prada-Pedreros, 2010 | X | X |  | X |  |  |  | García-Alzate et al. (2010a) |
| *Hyphessobrycon niger* García-Alzate, Román-Valencia & Prada-Pedreros, 2010 | X | X |  | X |  |  |  | García-Alzate et al. (2010a) |
| *Hyphessobrycon sebastiani* García-Alzate, Román-Valencia & Taphorn, 2010 | X | X |  |  |  | X |  | García-Alzate et al. (2010c) |
| *Hyphessobrycon taguae* García-Alzate, Román-Valencia & Taphorn, 2010 | X | X | X | X |  |  |  | García-Alzate et al. (2010b) |
| *Astyanacinus yariguies* Torres-Mejia, Hernández & Senechal, 2012 | X | X |  |  | X |  |  | Torres-Mejia et al. (2012) |
| *Tyttocharax metae* Román-Valencia, García-Alzate, Ruiz-C. & Taphorn B., 2012 | X | X |  | X |  |  |  | Román-Valencia et al. (2012) |
| *Gephyrocharax torresi* Vanegas-Ríos, Azpelicueta. Mirande & García Gonzales, 2013 | X | X |  |  | X |  |  | Vanegas-Ríos et al. (2013a) |
| *Hemibrycon antioquiae* Román-Valencia, Ruiz-C., Taphorn, Mancera-Rodriguez & García-Alzate, 2013 | X | X |  |  | X |  |  | Román-Valencia et al. (2013) |
| *Hemibrycon cardalensis* Román-Valencia, Ruiz-C., Taphorn, Mancera-Rodriguez & García-Alzate, 2013 | X | X |  |  | X |  |  | Román-Valencia et al. (2013) |
| *Hemibrycon fasciatus* Román-Valencia, Ruiz-C., Taphorn, Mancera-Rodriguez & García-Alzate, 2013 | X | X |  |  | X |  |  | Román-Valencia et al. (2013) |
| *Hyphessobrycon chocoensis* García-Alzate, Román-Valencia & Taphorn, 2013 | X | X |  |  |  | X |  | García-Alzate et al. (2013) |
| *Bryconamericus caldasi* Román-Valencia, Ruiz-C., Taphorn B. & García-Alzate, 2014 | X | X |  |  | X |  |  | Román-Valencia et al. (2014a) |
| *Hemibrycon sanjuanensis* Román-Valencia, Ruiz-C., Taphorn & García-Alzate, 2014 | X | X |  |  |  | X |  | Román-Valencia et al. (2014b) |
| *Chrysobrycon guahibo* Vanegas-Ríos, Urbano-Bonilla & Azpelicueta, 2015 | X | X |  | X |  |  |  | Vanegas-Ríos et al. (2015) |
| *Hemibrycon sierraensis* García-Alzate, Román-Valencia & Taphorn, 2015 | X | X |  |  |  |  | X | García-Alzate et al. (2015a) |
| *Hemigrammus rubrostriatus* Zarske, 2015 | X |  | X |  |  |  |  | Zarske (2015) |
| *Hyphessobrycon natagaima* García-Alzate, Taphorn, Román-Valencia & Villa-Navarro, 2015 | X | X |  |  | X |  |  | García-Alzate et al. (2015b) |
| *Hemigrammus aguaruna* Lima, Correa & Ota, 2016 |  |  | X |  |  |  |  | Lima et al. (2016) |
| *Chrysobrycon mojicai* Vanegas-Ríos & Urbano-Bonilla 2017 | X | X | X |  |  |  |  | Vanegas-Ríos and Urbano-Bonilla (2017) |
| *Hyphessobrycon klausanni* García-Alzate, Urbano-Bonilla & Taphorn 2017 | X | X |  | X |  |  |  | García-Alzate et al. (2017) |
| **Gymnotiformes (6)** |  |  |  |  |  |  |  |  |
| **Hypopomidae (5)** |  |  |  |  |  |  |  |  |
| *Brachyhypopomus bullocki* Sullivan & Hopkins, 2009 | X |  |  | X |  |  |  | Sullivan and Hopkins (2009) |
| *Brachyhypopomus* (*Odontohypopomus*) *bennetti* Sullivan, Zuanon & Cox, Fernandes 2013 |  |  | X |  |  |  |  | Sullivan et al. (2013b) |
| *Brachyhypopomus batesi* Crampton, de Santana, Waddell & Lovejoy, 2016 |  |  | X |  |  |  |  | Crampton et al. (2016) |
| *Brachyhypopomus flavipomus* Crampton, de Santana, Waddell & Lovejoy, 2016 |  |  | X |  |  |  |  | Crampton et al. (2016) |
| *Brachyhypopomus sullivani* Crampton, de Santana, Waddell & Lovejoy, 2016 |  |  |  | X |  |  |  | Crampton et al. (2016) |
| **Apteronotidae (1)** |  |  |  |  |  |  |  |  |
| *Apteronotus anu* de Santana & Vari, 2013 |  |  |  |  |  |  | X | de Santana and Vari (2013) |
| **Siluriformes (47)** |  |  |  |  |  |  |  |  |
| **Trichomycteridae (13)** |  |  |  |  |  |  |  |  |
| *Trichomycterus ballesterosi* Ardila Rodríguez, 2011 | X | X |  |  |  |  | X | Ardila Rodríguez (2011a) |
| *Trichomycterus maldonadoi* Ardila Rodríguez, 2011 | X | X |  |  |  |  | X | Ardila Rodríguez (2011b) |
| *Trichomycterus ocanaensis* Ardila Rodríguez, 2011 | X | X |  |  |  |  | X | Ardila Rodríguez (2011d) |
| *Trichomycterus sketi* Castellanos-Morales, 2011 | X | X |  |  | X |  |  | Castellanos-Morales (2011) |
| *Trichomycterus nietoi* Ardila Rodríguez, 2014 | X | X |  |  |  |  | X | Ardila Rodríguez (2014) |
| *Trichomycterus steindachneri* DoNascimiento, Prada-Pedreros & Guerrero-Kommritz, 2014 | X | X |  | X |  |  |  | DoNascimiento et al. (2014b) |
| *Trichomycterus arhuaco* Ardila-Rodríguez, 2016 | X | X |  |  | X |  |  | Ardila Rodríguez (2016c) |
| *Trichomycterus garciamarquezi* Ardila-Rodríguez, 2016 | X | X |  |  |  |  | X | Ardila Rodríguez (2016c) |
| *Trichomycterus kankuamo* Ardila-Rodríguez, 2016 | X | X |  |  | X |  |  | Ardila Rodríguez (2016c) |
| *Trichomycterus manaurensis* Ardila-Rodríguez, 2016 | X | X |  |  | X |  |  | Ardila Rodríguez (2016c) |
| *Trichomycterus montesi* Ardila-Rodríguez, 2016 | X | X |  |  | X |  |  | Ardila Rodríguez (2016c) |
| *Trichomycterus tetuanensis* García-Melo, Villa-Navarro & DoNascimiento, 2016 | X | X |  |  | X |  |  | García-Melo et al. (2016) |
| *Trichomycterus torcoromaensis* Ardila Rodríguez, 2016 | X | X |  |  | X |  |  | Ardila Rodríguez (2016a) |
| **Astroblepidae (16)** |  |  |  |  |  |  |  |  |
| *Astroblepus acostai* Ardila Rodríguez, 2011 | X | X |  |  |  |  | X | Ardila Rodríguez (2011e) |
| *Astroblepus cacharas* Ardila Rodríguez, 2011 | X | X |  |  | X |  |  | Ardila Rodríguez (2011c) |
| *Astroblepus itae* Ardila Rodríguez, 2011 | X | X |  |  | X |  |  | Ardila Rodríguez (2011e) |
| *Astroblepus ardilai* Ardila Rodríguez, 2012 | X | X |  |  | X |  |  | Ardila Rodríguez (2012) |
| *Astroblepus jimenezae* Ardila Rodríguez, 2013 | X | X |  |  |  |  | X | Ardila Rodríguez (2013a) |
| *Astroblepus martinezi* Ardila Rodríguez, 2013 | X | X |  |  |  |  | X | Ardila Rodríguez (2013a) |
| *Astroblepus ardiladuartei* Ardila Rodríguez, 2015 | X | X |  |  | X |  |  | Ardila Rodríguez (2015a) |
| *Astroblepus bellezaensis* Ardila Rodríguez, 2015 | X | X |  |  | X |  |  | Ardila Rodríguez (2015a) |
| *Astroblepus curitiensis* Ardila Rodríguez, 2015 | X | X |  |  | X |  |  | Ardila Rodríguez (2015b) |
| *Astroblepus mojicai* Ardila Rodríguez, 2015 | X | X |  |  |  | X |  | Ardila Rodríguez (2015a) |
| *Astroblepus nettoferreirai* Ardila Rodríguez, 2015 | X | X |  |  | X |  |  | Ardila Rodríguez (2015a) |
| *Astroblepus onzagaensis* Ardila Rodríguez, 2015 | X | X |  |  | X |  |  | Ardila Rodríguez (2015b) |
| *Astroblepus pradai* Ardila Rodríguez, 2015 | X | X |  |  | X |  |  | Ardila Rodríguez (2015b) |
| *Astroblepus putumayoensis* Ardila Rodríguez, 2015 | X | X | X |  |  |  |  | Ardila Rodríguez (2015a) |
| *Astroblepus verai* Ardila Rodríguez, 2015 | X | X |  |  | X |  |  | Ardila Rodríguez (2015b) |
| *Astroblepus floridablancaensis* Ardila Rodríguez, 2016 | X | X |  |  | X |  |  | Ardila Rodríguez (2016b) |
| **Loricariidae (13)** |  |  |  |  |  |  |  |  |
| *Hypoptopoma bianale* Aquino & Schaefer, 2010 | X |  | X |  |  |  |  | Aquino and Schaefer (2010) |
| *Hypoptopoma brevirostratum* Aquino & Schaefer, 2010 | X |  | X |  |  |  |  | Aquino and Schaefer (2010) |
| *Chaetostoma formosae* Ballen, 2011 | X | X |  | X |  |  |  | Ballen (2011) |
| *Ancistrus tolima* Taphorn, Armbruster, Villa-Navarro & Ray, 2013 | X | X |  |  | X |  |  | Taphorn et al. (2013) |
| *Ancistrus vericaucanus* Taphorn, Armbruster, Villa-Navarro & Ray, 2013 | X | X |  |  | X |  | X | Taphorn et al. (2013) |
| *Chaetostoma floridablancaense* Ardila Rodríguez, 2013 | X | X |  |  | X |  |  | Ardila Rodríguez (2013b) |
| *Farlowella yarigui* Ballen & Mojica, 2014 | X | X |  |  | X |  |  | Ballen and Mojica (2014) |
| *Parotocinclus variola* Lehmann, Schvambach & Reis, 2015 | X | X | X |  |  |  |  | Lehmann et al. (2015) |
| *Peckoltia lujani* Armbruster, Werneke & Tan, 2015 |  |  |  | X |  |  |  | Armbruster et al. (2015) |
| *Chaetostoma joropo* Ballen, Urbano-Bonilla & Maldonado-Ocampo, 2016 | X | X |  | X |  |  |  | Ballen et al. (2016a) |
| *Dasyloricaria paucisquama* Londoño-Burbano & Reis, 2016 | X | X |  |  | X |  |  | Londoño-Burbano and Reis (2016) |
| *Farlowella mitoupibo* Ballen, Urbano-Bonilla & Zamudio, 2016 | X | X |  |  |  |  |  | Ballen et al. (2016b) |
| *Cordylancistrus pijao* Provenzano R. & Villa-Navarro, 2017 | X | X |  |  | X |  |  | Provenzano R. and Villa-Navarro (2017) |
| **Doradidae (1)** |  |  |  |  |  |  |  |  |
| *Nemadoras cristinae* Sabaj Pérez, Arce H., Sousa & Birindelli, 2014 | X |  | X | X |  |  |  | Sabaj Pérez et al. (2014) |
| **Heptapteridae (3)** |  |  |  |  |  |  |  |  |
| *Imparfinis timana* Ortega-Lara, Milani, DoNascimiento, Villa-Navarro & Maldonado-Ocampo, 2011 | X | X |  |  | X |  |  | Ortega-Lara et al. (2011) |
| *Imparfinis usmai* Ortega-Lara, Milani, DoNascimiento, Villa-Navarro & Maldonado-Ocampo, 2011 | X | X |  |  | X | X |  | Ortega-Lara et al. (2011) |
| *Pimelodella floridablancaensis* Ardila Rodríguez 2017 | X | X |  |  | X |  |  | Ardila Rodríguez (2017) |
| **Pseudopimelodidae (1)** |  |  |  |  |  |  |  |  |
| *Rhyacoglanis annulatus* Shibatta & Vari, 2017 |  |  |  | X |  |  |  | Shibatta & Vari (2017) |
| **Pleuronectiformes (1)** |  |  |  |  |  |  |  |  |
| **Achiridae (1)** |  |  |  |  |  |  |  |  |
| *Trinectes hubbsbollinger* Duplain, Chapleau & Munroe, 2012 | X | X |  |  |  | X |  | Duplain et al. (2012) |
| **Cichliformes (5)** |  |  |  |  |  |  |  |  |
| **Cichlidae (5)** |  |  |  |  |  |  |  |  |
| *Apistogramma lineata* Mesa S. & Lasso, 2011 | X | X |  | X |  |  |  | Mesa S. and Lasso (2011b) |
| *Apistogramma megaptera* Mesa Salazar & Lasso, 2011 | X |  |  | X |  |  |  | Mesa Salazar and Lasso (2011a) |
| *Apistogramma minima* Mesa S. & Lasso, 2011 |  |  |  | X |  |  |  | Mesa S. and Lasso (2011b) |
| *Apistogramma piaroa* Mesa S. & Lasso, 2011 |  |  |  | X |  |  |  | Mesa S. and Lasso (2011b) |
| *Crenicichla monicae* Kullander & Varella, 2015 |  |  | X |  |  |  |  | Kullander and Varella (2015) |
| **Cyprinodontiformes (5)** |  |  |  |  |  |  |  |  |
| **Cynolebiidae (5)** |  |  |  |  |  |  |  |  |
| *Rivulus* (*Cynodonichthys*) *azurescens* Vermeulen, 2013 | X | X |  |  | X |  |  | Vermeulen (2013) |
| *Rivulus* (*Cynodonichthys*) *pivijay* Vermeulen, 2013 | X | X |  |  | X |  |  | Vermeulen (2013) |
| *Rivulus* (*Cynodonichthys*) *ribesrubrum* Vermeulen, 2013 | X | X |  |  | X |  |  | Vermeulen (2013) |
| *Rivulus* (*Cynodonichthys*) *xi* Vermeulen, 2013 | X | X |  |  | X |  |  | Vermeulen (2013) |
| *Laimosemion leticia* Valdesalici, 2016 | X | X | X |  |  |  |  | Valdesalici (2016) |
| ***Species inadvertently omitted in Maldonado-Ocampo *et al*. (2008)** | | | | | | | | |
| **Siluriformes (1)** |  |  |  |  |  |  |  |  |
| **Cetopsidae (1)** |  |  |  |  |  |  |  |  |
| **Denticetopsis seducta* Vari, Ferraris & de Pinna, 2005 |  |  |  | X |  |  |  | Vari et al. (2005) |
| **Cyprinodontiformes (2)** |  |  |  |  |  |  |  |  |
| **Cyprinodontidae (1)** |  |  |  |  |  |  |  |  |
| **Yssolebias martae* (Steindachner, 1876) | X | X |  |  |  |  | X | Steindachner (1876) |
| **Poeciliidae (1)** |  |  |  |  |  |  |  |  |
| **Poecilia koperi* Poeser, 2003 |  |  |  |  |  |  | X | Poeser (2003b) |
