# Supplementary material for: Checklist of the freshwater fishes of Colombia: a Darwin Core alternative to the updating problem
Source: Zookeys. 2017 Oct 13;(708):25–138. doi: 10.3897/zookeys.708.13897 (PMC5674168; doi:10.3897/zookeys.708.13897)
Supplement: Supplementary material 3 — New records of species for Colombia after Maldonado-Ocampo et al. (2008) [file zookeys-708-025-s003.docx]

**SUPPLEMENTARY FILE 3**

New records of species for Colombia after Maldonado-Ocampo *et al*. (2008). Numbers in parenthesis following each taxon name indicate the number of species added to each taxon.

| **Taxa** | **Amz** | **Ori** | **Mag-Cauc** | **Pac** | **Car** | **Collections/References** |
| --- | --- | --- | --- | --- | --- | --- |
| **Myliobatiformes (3)** |  |  |  |  |  |  |
| **Potamotrygonidae (3)** |  |  |  |  |  |  |
| *Heliotrygon gomesi* Carvalho & Lovejoy, 2011 | X |  |  |  |  | Lasso et al. (2014) |
| *Plesiotrygon nana* Carvalho & Ragno, 2011 | X |  |  |  |  | IAvH-P 11910-11915, IMCN 6336; Lasso et al. (2014) |
| *Potamotrygon scobina* Garman, 1913 |  | X |  |  |  | IAvH-P 11962; Lasso et al. (2014) |
| **Characiformes (53)** |  |  |  |  |  |  |
| **Crenuchidae (2)** |  |  |  |  |  |  |
| *Elachocharax mitopterus* Weitzman, 1986 | X |  |  |  |  | CZUT-IC 4071, 4086, 5106 |
| *Melanocharacidium* *nigrum* Buckup, 1993 | X |  |  |  |  | IAvH-P 8660, 87008765, 8796, 8859, 9421 |
| **Erythrinidae (1)** |  |  |  |  |  |  |
| *Hoplias curupira* Oyakawa & Mattox, 2009 |  | X |  |  |  | IAvH-P 1349, 2274, 2407, 2743 |
| **Serrasalmidae (1)** |  |  |  |  |  |  |
| *Serrasalmus eigenmanni* Norman, 1929 | X |  |  |  |  | CZUT-IC 7334 |
| **Hemiodontidae (1)** |  |  |  |  |  |  |
| *Hemiodus atranalis* (Fowler, 1940) | X |  |  |  |  | IAvH-P 2389 |
| **Anostomidae (3)** |  |  |  |  |  |  |
| *Anostomoides* *laticeps* (Eigenmann, 1912) | X |  |  |  |  | XIII Congreso Colombiano de Ictiología |
| *Leporinus* *amazonicus* Santos & Zuanon, 2008 | X |  |  |  |  | XIII Congreso Colombiano de Ictiología |
| *Leporinus parae* Eigenmann, 1907 | X | X |  |  |  | IAvH-P 1822, 12770, 13003, 13011. Identifications based on Britski and Birindelli (2008) |
| **Chilodontidae (1)** |  |  |  |  |  |  |
| *Caenotropus schizodon* Scharcansky & Lucena, 2007 | X |  |  |  |  | CZUT-IC 12280 |
| **Curimatidae (2)** |  |  |  |  |  |  |
| *Curimatopsis microlepis* Eigenmann & Eigenmann, 1889 | X |  |  |  |  | CZUT-IC 4073, 4075 |
| *Cyphocharax notatus* (Steindachner, 1908) | X |  |  |  |  | CZUT-IC 10874 |
| **Lebiasinidae (2)** |  |  |  |  |  |  |
| *Copella meinkeni* Zarske & Géry, 2006 |  | X |  |  |  | IMCN 6328; Ortega-Lara (2016) |
| *Nannostomus digrammus* (Fowler, 1913) | X |  |  |  |  | IAvH-P 10688, 10692, 10694 |
| **Acestrorhynchidae (1)** |  |  |  |  |  |  |
| *Heterocharax virgulatus* Toledo-Piza, 2000 |  | X |  |  |  | IAvH-P 14091 |
| **Characidae (34)** |  |  |  |  |  |  |
| *Charax notulatus* Lucena, 1987 |  | X |  |  |  | IAvH-P 7521, 13028, 13073 |
| *Phenacogaster maculoblonga* Lucena & Malabarba, 2010 |  | X |  |  |  | CZUT-IC 9680 |
| *Phenacogaster napoatilis* Lucena & Malabarba, 2010 | X |  |  |  |  | CZUT-IC 9008 |
| *Phenacogaster prolata* Lucena & Malabarba, 2010 |  | X |  |  |  | CZUT-IC 7223 |
| *Hemigrammus geisleri* Zarske & Géry, 2007 | X | X |  |  |  | CZUT-IC 4400, 5097, 8655, 8851, 8933, 8954, 9099, 9245, 9993, 14642, 16138, 16309, 16837, 16934, MPUJ 306, 1232, 1234, 1237, 1357, 4378, 4392, 10804 |
| *Hemigrammus yinyang* Lima & Sousa, 2009 | X |  |  |  |  | CZUT-IC 4323, 4467, 4553, 4857, MPUJ 12525, 12623-12631 |
| *Hyphessobrycon dorsalis* Zarske, 2014 |  | X |  |  |  | IAvH-P 1032, 2315 |
| *Hyphessobrycon epicharis* Weitzman & Palmer, 1997 | X |  |  |  |  | CZUT-IC 4171, 5098 |
| *Hyphessobrycon heterorhabdus* (Ulrey, 1894) | X |  |  |  |  | IAvH-P 8343, 8345; García-Alzate *et al*. (2008, 2010) |
| *Hyphessobrycon otrynus* Benine & Lopes, 2008 |  | X |  |  |  | MCGN 55760 in Ota *et al*. (2015) |
| *Moenkhausia hysterosticta* Lucinda, Malabarba & Benine, 2007 |  | X |  |  |  | CZUT-IC 9821, MPUJ 4387, 4468 |
| *Moenkhausia justae* Eigenmann, 1908 |  | X |  |  |  | IAvH-P 847 |
| *Moenkhausia latissima* Eigenmann, 1908 | X |  |  |  |  | IAvH-P 11014-11015 |
| *Moenkhausia mikia* Marinho & Langeani, 2010 | X |  |  |  |  | CZUT-IC 3498, 3555, 3584, 4910, 4918, 5104, 8069 |
| *Aphyocharax colifax* Taphorn & Thomerson, 1991 | X |  |  |  |  | CZUT-IC 4271 |
| *Microschemobrycon melanotus* (Eigenmann, 1912) |  | X |  |  |  | MPUJ 236, 247, 630, 1436, 4497 |
| *Oxybrycon parvulus* Géry 1964 | X |  |  |  |  | CZUT-IC 17910, 17953 |
| *Parecbasis cyclolepis* Eigenmann, 1914 | X |  |  |  |  | CIACOL 272 |
| *Tyttobrycon xeruini* Géry, 1973 |  | X |  |  |  | IAvH-P-14408, 15186, 15208, 15216, 15233, 15244, 15256, 15358, 15391, 15427, 15433, 15469, 15482, 15517, 15556, 15573, 15597, MPUJ 11025 |
| *Odontostilbe pao* Bührnheim & Malabarba, 2007 |  |  |  |  |  | Urbano-Bonilla et al. (2009) |
| *Scopaeocharax atopodus* (Böhlke, 1958) | X |  |  |  |  | CIACOL 2713 |
| *Creagrutus barrigai* Vari & Harold, 2001 | X |  |  |  |  | CZUT-IC 12062 |
| *Creagrutus runa* Vari & Harold, 2001 | X |  |  |  |  | CZUT-IC 4117, 4163 |
| *Ceratobranchia binghami* Eigenmann, 1927 | X |  |  |  |  | CZUT-IC 12031, 12033 |
| *Ceratobranchia joanae* Chernoff & Machado-Allison, 1990 |  | X |  |  |  | CZUT-IC 12827 |
| *Knodus gamma* Géry, 1972 | X |  |  |  |  | CZUT-IC 12073, 12967 |
| *Rhinobrycon negrensis* Myers, 1944 |  | X |  |  |  | CZUT-IC 5081 |
| *Cyanogaster noctivaga* Mattox, Britz, Toledo-Piza & Marinho, 2013 | X | X |  |  |  | CZUT-IC 18065, MPUJ 273, 1257, 9846-9847, 10726-10729, 10744 |
| *Bryconamericus macrophthalmus* Román-Valencia, 2003 | X |  |  |  |  | CZUT-IC 5124 |
| *Bryconamericus orinocoense* Román-Valencia, 2003 | X |  |  |  |  | CZUT-IC 4886, 4916 |
| *Astyanax anterior* Eigenmann, 1908 | X |  |  |  |  | IAvH-P 8238-8240, 9117, 11012 |
| *Astyanax guianensis* Eigenmann, 1909 | X |  |  |  |  | Marinho *et al*. (2015) |
| *Brittanichthys axelrodi* Géry, 1965 |  | X |  |  |  | Weitzman et al. (2005) |
| *Ctenobrycon oliverai* Benine, Lopes & Ron, 2010 |  | X |  |  |  | CZUT-IC 7217, 9056, 9279, 9321, 9670, 9752, 9785, 9801, 9810, 9851 |
| **Bryconidae (2)** |  |  |  |  |  |  |
| *Brycon hilarii* (Valenciennes, 1850) | X |  |  |  |  | Lima (2017) |
| *Brycon polylepis* Moscó Morales, 1988 |  | X |  |  |  | Lima (2017) |
| **Triportheidae (2)** |  |  |  |  |  |  |
| *Triportheus culter* (Cope, 1872) | X |  |  |  |  | CZUT-IC 7310 |
| *Triportheus rotundatus* (Jardine, 1841) | X |  |  |  |  | IAvH-P 11059 |
| **Iguanodectidae (1)** |  |  |  |  |  |  |
| *Bryconops magoi* Chernoff & Machado-Allison, 2005 | X |  |  |  |  | CZUT-IC 4129 |
| **Gymnotiformes (6)** |  |  |  |  |  |  |
| **Rhamphichthyidae (1)** |  |  |  |  |  |  |
| *Rhamphichthys apurensis* (Fernández-Yépez, 1968) |  | X |  |  |  | CZUT-IC 3244, 5178, 5179, 9362, 9421, 9422, 9423, 9424, 9448, 9449, 9547 |
| **Sternopygidae (2)** |  |  |  |  |  |  |
| *Rhabdolichops eastwardi* Lundberg & Mago-Leccia, 1986 | X |  |  |  |  | CZUT-IC 3642, 3987 |
| *Rhabdolichops zareti* Lundberg & Mago-Leccia, 1986 |  | X |  |  |  | CZUT-IC 9471 |
| **Apteronotidae (3)** |  |  |  |  |  |  |
| *Adontosternarchus sachsi* (Peters, 1877) |  | X |  |  |  | CZUT-IC 9438, 9451, 9453, 9455, 9457, 9475, 9490, 9492 |
| *Sternarchella orinoco* Mago-Leccia, 1994 |  | X |  |  |  | CZUT-IC 9493, 9535 |
| *Sternarchella orthos* Mago-Leccia, 1994 |  | X |  |  |  | CZUT-IC 9445, 9482, 9484 |
| **Siluriformes (47)** |  |  |  |  |  |  |
| **Trichomycteridae (2)** |  |  |  |  |  |  |
| *Trichomycterus emanueli* (Schultz, 1944) |  |  |  |  | X | IAvH-P 9797 |
| *Tridentopsis pearsoni* Myers, 1925 | X |  |  |  |  | CZUT-IC 17989, 18069 |
| **Callichthyidae (2)** |  |  |  |  |  |  |
| *Corydoras concolor* Weitzman, 1961 |  | X |  |  |  | CZUT-IC 11794, 11892 |
| *Corydoras crypticus* Sands, 1995 | X |  |  |  |  | CZUT-IC 11837, IMCN 5833-5834; |
| **Loricariidae (17)** |  |  |  |  |  |  |
| *Acestridium dichromum* Retzer, Nico & Provenzano, 1999 | X | X |  |  |  | IAvH-P 9946-9948, 10704 |
| *Farlowella taphorni* Retzer & Page, 1997 |  |  |  |  | X | IAvH-P 9800-9801 |
| *Lamontichthys filamentosus* (La Monte, 1935) | X |  |  |  |  | IAvH-P 1610, 1617 |
| *Loricariichthys hauxwelli* (Hancock, 1828) | X |  |  |  |  | IAvH-P 12562 |
| *Rineloricaria daraha* Rapp Py-Daniel & Fichberg,  2008 | X |  |  |  |  | Bogotá-Gregory et al. (2016) |
| ‘*Hemiancistrus*’ *subviridis* Werneke, Sabaj, Lujan & Armbruster, 2005 |  | X |  |  |  | IMCN 5897, 6094, 6189, 6195-6196, 6284, 6574-6575 |
| *Hypancistrus furunculus* Armbruster, Lujan & Taphorn, 2007 |  | X |  |  |  | IMCN 5828, 6017-6018, 6297-6300; Lasso et al. (2009) |
| *Hypancistrus inspector* Armbruster, 2002 |  | X |  |  |  | Ortega-Lara (2016) |
| *Leporacanthicus triactis* Isbrücker, Nijssen & Nico, 1992 |  | X |  |  |  | IAvH-P 8564, IMCN 5772-5774 |
| *Leptoancistrus canensis* (Meek & Hildebrand, 1913) |  |  |  |  | X | Maldonado-Ocampo et al (2013b) |
| *Panaque titan* Lujan, Hidalgo & Stewart, 2010 | X |  |  |  |  | IMCN 6059 |
| *Peckoltia caenosa* Armbruster, 2008 |  | X |  |  |  | IMCN 7061 |
| *Pseudoancistrus sidereus* Armbruster, 2004 |  | X |  |  |  | IMCN 6232, 6234 |
| *Hypostomus* *robinii* Valenciennes, 1840 |  | X |  |  |  | IAvH-P 423, 736, 4957, 5372-5373, 5495, 5502, 5504; Armbruster *et al*. (2007) |
| *Pterygoplichthys zuliaensis* Weber, 1991 |  |  |  |  | X | Ortega-Lara et al. (2012) |
| *Pseudolithoxus anthrax* (Armbruster y Provenzano, 2000) |  | X |  |  |  | IMCN 5874, 5880-5881, 6063-6064, 6231, 6601-6604; Lasso et al. (2009) |
| *Pseudolithoxus kelsorum* Lujan & Birindelli, 2011 |  | X |  |  |  | IMCN 5876-5877 |
| **Aspredinidae (2)** |  |  |  |  |  |  |
| *Bunocephalus aloikae* Hoedeman, 1961 |  | X |  |  |  | IAvH-P 3275, 3280-3281, 12041, 12065, 13038 |
| *Pterobunocephalus depressus* (Haseman, 1911) | X | X |  |  |  | IAvH-P 8674, 8714, 8779, 8814, 8844, 8872, 8903, 11991 |
| **Auchenipteridae (7)** |  |  |  |  |  |  |
| *Centromochlus macracanthus*, Soares-Porto 2000 | X |  |  |  |  | IAvH-P 14318 |
| *Tatia marthae* Vari & Ferraris, 2013 |  | X |  |  |  | IAvH-P 12458, 12463, 12473, 12483, 12496, 12861, 12870, 12873 |
| *Tatia nigra* Sarmento-Soares & Martins-Pinheiro, 2008 | X | X |  |  |  | CZUT-IC 4441, 4544, IAvH-P 12862, 12874 |
| *Tatia strigata* Soares-Porto, 1995 | X | X |  |  |  | CZUT-IC 4527, IAvH-P 5634, 10049, 10761, 12577, 12579, 12581-12583 |
| *Ageneiosus dentatus* Kner, 1857 |  |  |  |  |  | Specimens from the Orinoco River Basin formerly recorded as *Ageneiosus ucayalensis* (see Ribeiro *et al*. 2017 for details on similarities of both species) |
| *Asterophysus batrachus* Kner, 1858 |  | X |  |  |  | IMCN 5528-5530, 5964, 6116-6124, 6433-6434 |
| *Auchenipterichthys coracoideus* Eigenmann & Allen, 1942 | X |  |  |  |  | Galvis *et al*. (2007b) as *Auchenipterichthys thoracatus* |
| **Doradidae (6)** |  |  |  |  |  |  |
| *Acanthodoras depressus* (Steindachner, 1881) | X |  |  |  |  | CZUT-IC 4312 |
| *Astrodoras* | X |  |  |  |  | Roa-Fuentes et al. (2010) |
| *Centrodoras hasemani* (Steindachner, 1915) | X |  |  |  |  | CZUT-IC 14832 |
| *Leptodoras rogersae* Sabaj Pérez, 2005 |  | X |  |  |  | CZUT-IC 9529 |
| *Lithodoras dorsalis* (Valenciennes, 1840) | X |  |  |  |  | CZUT-IC 14500 |
| *Rhinodoras boehlkei* Glodek, Whitmire & Orcés V., 1976 |  | X |  |  |  | CZUT-IC 12272, 12318 |
| **Heptapteridae (10)** |  |  |  |  |  |  |
| *Brachyrhamdia imitator* Myers, 1927 |  | X |  |  |  | IMCN 5691 |
| *Brachyrhamdia meesi* Sands & Black, 1985 | X |  |  |  |  | IAvH-P 11146 |
| *Brachyrhamdia thayeria* Slobodian & Bockmann, 2013 | X |  |  |  |  | Galvis *et al*. (2007b) as *Brachyrhamdia* sp |
| *Gladioglanis machadoi* Lundberg, Mago-Leccia & Nass, 1991 | X |  |  |  |  | CZUT-IC 5091, 5126, IAvH-P-9998; Maldonado-Ocampo et al. (2006a) |
| *Phenacorhamdia anisura* (Mees, 1987) |  | X |  |  |  | IAvH-P 12894 |
| *Phenacorhamdia provenzanoi* DoNascimiento & Milani, 2008 |  | X |  |  |  | CZUT-IC 6351, 6881, 6988 |
| *Phenacorhamdia taphorni* DoNascimiento & Milani, 2008 |  | X |  |  |  | IAvH-P 7932, 9254, 9594, 10727 |
| *Pimelodella cruxenti* Fernández-Yépez, 1950 |  | X |  |  |  | CZUT-IC 7180, 7192 |
| *Rhamdia guatemalensis* (Günther, 1864) |  |  | X | X | X | Perdices et al. (2002), Hernández et al. (2015) |
| *Rhamdia saijaensis* Rendahl, 1941 |  |  |  | X |  | Hernández et al. (2015) |
| **Pimelodidae (1)** |  |  |  |  |  |  |
| *Exallodontus aguanai* Lundberg, Mago-Leccia & Nass, 1991 |  | X |  |  |  | CZUT-IC 9407, 9420, 9553, 9660, 9708, 9727, 9733, 9745, 9842 |
| **Pleuronectiformes (1)** |  |  |  |  |  |  |
| **Achiridae (1)** |  |  |  |  |  |  |
| *Apionichthys nattereri* (Steindachner, 1876) | X |  |  |  |  | IAvH-P 3994 |
| **Cichliformes (3)** |  |  |  |  |  |  |
| **Cichlidae (3)** |  |  |  |  |  |  |
| *Crenicichla zebrina* Montaña, López-Fernández & Taphorn, 2008 |  | X |  |  |  | IMCN 6476 |
| *Apistogramma flabellicauda* Mesa S. & Lasso, 2011 | X | X |  |  |  | CZUT-IC 1494, IAvH-P 10087, 10089-10092, 10094-10098 |
| *Laetacara fulvipinnis* Staeck & Schindler, 2007 |  | X |  |  |  | CZUT-IC 8653 |
| **Cyprinodontiformes (2)** |  |  |  |  |  |  |
| **Poeciliidae (2)** |  |  |  |  |  |  |
| *Priapichthys darienensis* (Meek & Hildebrand, 1913) |  |  |  |  | X | CZUT-IC 11742 |
| *Poecilia gillii* (Kner, 1863) |  |  |  |  | X | Poeser (2003a) |
