# Supplementary material for: Checklist of the freshwater fishes of Colombia: a Darwin Core alternative to the updating problem
Source: Zookeys. 2017 Oct 13;(708):25–138. doi: 10.3897/zookeys.708.13897 (PMC5674168; doi:10.3897/zookeys.708.13897)
Supplement: Supplementary material 4 — Species listed in Maldonado-Ocampo et al. (2008) with geographic distribution corrected [file zookeys-708-025-s004.docx]

**SUPPLEMENTARY FILE 4**

**Species listed in Maldonado-Ocampo et al. (2008) with geographic distribution corrected.**

**MYLIOBATIFORMES**

**Potamotrygonidae**

***Potamotrygon orbignyi*** (Kner, 1858)

Distribution extended to include the Amazon River Basin (Lasso et al. 2013b).

**CLUPEIFORMES**

**Engraulidae**

***Anchoviella guianensis*** (Eigenmann, 1912)

Presence in the Amazon River Basin was based on Ortega et al. (2006), which only listed Peruvian collections. Restricted to the Orinoco River Basin.

**CHARACIFORMES**

**Crenuchidae**

***Characidium boavistae*** Steindachner, 1915

This species apparently has a wide distribution in the Lago de Maracaibo Basin, Orinoco and northern tributaries of the Amazon Basin (Buckup 2003). In Colombia, additional to its presence in the Magdalena-Cauca system is also found in the Catatumbo River drainage (Caribbean system) (Ortega-Lara et al. 2012) and Orinoco system (Urbano-Bonilla et al. 2009).

***Characidium chupa*** Schultz, 1944

Originally described from the Lago de Maracaibo Basin, its presence in the Catatumbo River drainage (Caribbean system) was inadvertently omitted and is supported by the following lots: IAvH-P 9, 9759, 9762-9764, 11719.

***Characidium sanctjohanni*** Dahl, 1960

Only known from the holotype (Agudelo-Zamora et al. 2014), which comes from the San Juan River Basin (Pacific system). Its presence in the Caribbean Versant Rivers (Maldonado-Ocampo et al. 2008) is not supported, neither its purported presence in the Atrato River Basin (Maldonado-Ocampo et al. 2013b). Removed from the Caribbean system.

***Melanocharacidium dispilomma*** Buckup, 1993

Bogotá-Gregory and Maldonado-Ocampo (2006a) listed Buckup (1993) as supporting its presence in the Colombian Amazon, but all the Colombian localities listed by Buckup (1993) are from the Orinoco River Basin.

**Parodontidae**

***Parodon suborbitalis*** Valenciennes, 1850

Restricted to the Catatumbo River drainage in Colombia, Caribbean system (Londoño-Burbano et al. 2011).

**Serrasalmidae**

***Myloplus asterias*** (Müller & Troschel, 1844)

Lasso et al. (2005) listed IAvH-P, however the only two records from the Orinoco River Basin at this collection (IAvH-P 10317-10318), actually correspond to *Mylopus rubripinnis* (Müller & Troschel 1844).

***Serrasalmus irritans*** Peters, 1877

Endemic to the Orinoco River Basin (Jegú, 2003). Mojica (1999) listed this species only for the Orinoco River Basin. Removed from the Amazon system.

**Hemiodontidae**

***Hemiodus amazonum*** (Humboldt, 1821)

Only recorded in Colombia in the Vaupés River drainage, Amazon system (ICN-MHN 17168). Provisionally excluded from the Orinoco system, until specimens become available from this drainage, since the species has also been recorded from this system in Venezuela (Lasso et al. 2004).

***Hemiodus gracilis*** Günther, 1864

Provisionally excluded from the Amazon system. All available records in Colombian collections come from the Orinoco River Basin.

**Anostomidae**

***Anostomus ternetzi*** Fernández-Yépez, 1949

Bogotá-Gregory and Maldonado-Ocampo (2006a) listed ICN-MHN, but all lots from this collection come from the Orinoco River Basin. The other source that supports its inclusion in the Colombian Amazon is the unpublished dissertation of Calderón and Hincapié (2001), which is not followed here as a reliable reference. Removed from the Amazon system.

**Chilodontidae**

***Caenotropus mestomorgmatos*** Vari, Castro & Raredon, 1995

Distributed in the upper portions of the Orinoco and Negro River basins in Brazil and Venezuela (Vari et al. 1995). Recorded for the Tomo River, Orinoco system in Colombia (IAvH-P 4098 and 4101). The species is also supported in the Amazon system by the lot IAvH-P 288, coming from the Putumayo River drainage.

***Chilodus gracilis*** Isbrücker & Nijssen, 1988

Distributed also in the Orinoco River Basin (USNM 232361, 263288, 431461).

**Curimatidae**

***Curimata cerasina*** Vari, 1984

Endemic to the Orinoco River Basin in Venezuela (Vari 2003). Originally listed for the Amazon system in Colombia in Bogotá-Gregory and Maldonado-Ocampo (2006a) and Maldonado-Ocampo et al. (2008), but lacking of taxonomically validated records in collections. However its presence in the Orinoco River Basin (Cusiana River) is supported by the lot CZUT-IC 11494.

***Steindachnerina argentea*** (Gill, 1858)

Distributed in rivers of the western portion of the island of Trinidad, the Orinoco River Basin, and some of the coastal rivers draining into the Caribbean Sea along the north coast of Venezuela (Vari 1991). All available lots at IAvH-P come from the Orinoco River Basin. Inclusion in the Amazon River Basin in Mojica et al. (2005) and Bogotá-Gregory and Maldonado-Ocampo (2006a) was probably based on misidentifications of other species occurring in this basin. Removed from the Amazon system.

**Lebiasinidae**

***Lebiasina erythrinoides*** (Valenciennes, 1850)

Originally described from the Lago de Maracaibo Basin. Presence in the Caribbean system was confirmed for the Catatumbo River drainage by Ortega-Lara et al. (2012) and is also supported by the lot IAvH-P 53 from the Ranchería River Basin. Additionally, its presence in the Pacific system is supported by lot IAvH-P 573.

***Lebiasina narinensis*** Ardila Rodríguez, 2002

Inclusion in the Magdalena-Cauca system is not supported by references or lots in collections.

***Pyrrhulina lugubris*** Eigenmann, 1922

Known only from the Orinoco River Basin in Colombia and Venezuela (Weitzman & Weitzman 2003).

**Ctenoluciidae**

***Boulengerella lucius*** (Cuvier, 1816)

Presence in the Amazon River Basin is not confirmed by verified lots in collections.

**Characidae**

***Gymnocorymbus thayeri*** Eigenmann, 1908

Distributed in the Corantijn and Amazon basins along with the Rio Gurupi and Rio Parnaíba of northeastern Brazil (Benine et al. 2015). Removed from the Orinoco system.

***Poptella compressa*** (Günther, 1864)

Present also in the Amazon system (Mojica et al. 2005), but inadvertently omitted in Maldonado-Ocampo et al. (2008).

***Parastremma pulchrum*** Dahl, 1960

Found only in the San Juan River Basin (Pacific system). The entry of the species in Cardoso (2003) states that the type locality is probably in error as the species is only found in the Atrato River Basin. However this is clearly a mistake since the holotype and the single paratype were directly collected by G. Dahl in two localities of the San Juan River, eliminating any question about the actual origin of the specimens.

***Roeboides dientonito*** Schultz, 1944

Widely distributed in the Lago de Maracaibo and Orinoco River Basin, and rivers of the north-western portion of Guyana (Lucena and Menezes, 2003). Distributed in the Catatumbo River (IAvH-P 11728, IAvH-P 11743), a tributary of the Lago de Maracaibo Basin.

***Roeboides occidentalis*** Meek & Hildebrand, 1916

Distributed in Colombia in rivers draining the Pacific slope (Lucena, 2000). The lot IAvH-P 7259 from the Atrato River Basin (Caribbean system) and listed in Maldonado-Ocampo et al. (2006b, 2013b) as supporting its presence in this river basin, actually corresponds to *Roeboides dayi* (Steindachner, 1878).

***Hemigrammus levis*** Durbin, 1908

Restricted to the Amazon River Basin (Lima et al. 2003). Records of this species in the Orinoco River Basin correspond to *H. micropterus* Meek, 1907 (Flávio C. T. Lima pers. com.).

***Hemigrammus stictus*** (Durbin, 1909)

Contrary to Bogotá-Gregory and Maldonado-Ocampo (2006a), the species was only listed for the Orinoco in Mojica (1999). All available records in Colombian collections come from the Orinoco system. Provisionally excluded from the Amazon system.

***Hyphessobrycon bentosi*** Durbin, 1908

Lasso et al. (2005) listed IAvH-P and IMCN as support for its inclusion in the Orinoco River Basin. However, all lots identified as this species in IAvH-P, actually correspond to *H. sweglesi* (Géry 1961) (Donald C. Taphorn pers. com.).

***Hyphessobrycon poecilioides*** Eigenmann, 1913

Known only from the Cauca and Magdalena River drainages (García-Alzate and Román-Valencia 2008, García-Alzate et al. 2015b).

***Hyphessobrycon sweglesi*** (Géry, 1961)

Restricted to the Orinoco River Basin (Lima et al. 2003). Presence in the Amazon River Basin is not supported by verified lots.

***Moenkhausia melogramma*** Eigenmann, 1908

Restricted to the Amazon River Basin (Lima et al. 2003). Lasso et al. (2005) listed the Vichada River and a single Venezuelan collection (MBUCV), but this collection lacks of records of this species not only from the Vichada River, but in general.

***Grundulus cochae*** Román-Valencia, Paepke & Pantoja, 2003

Described for Laguna de La Cocha (upper Putumayo River drainage, Amazon system) and indicated as endemic to this drainage (Román-Valencia et al. 2003).

***Nematobrycon lacortei*** Weitzman & Fink, 1971

Originally described from the río Calima (Pacific versant). Inclusion in the Caribbean system is not supported by lots.

***Eretmobrycon emperador*** (Eigenmann & Ogle, 1907)

Presence in the Magdalena-Cauca system is not supported by verified lots in collections.

***Eretmobrycon guaytarae*** (Eigenmann & Henn, 1914)

Presence in the Magdalena-Cauca system is not supported by verified lots in collections.

***Eretmobrycon miraensis*** (Fowler, 1945)

Presence in the Magdalena-Cauca system is not supported by verified lots in collections.

***Eretmobrycon scleroparius*** (Regan, 1908)

Presence in the Pacific system is not supported by verified lots in collections.

***Gephyrocharax caucanus*** Eigenmann, 1912

Distribution extended to include the Pacific system (Lago Calima) (Vanegas-Ríos 2016).

***Boehlkea fredcochui*** Géry, 1966

Known only from the Amazon River Basin (Lima et al. 2003). Presence in the Pacific and Caribbean systems is not supported by verified lots in collections.

***Hemibrycon carrilloi*** Dahl, 1960

Known only from the upper Atrato River Basin (Lima et al. 2003). Inclusion in the Pacific and Magdalena-Cauca systems is not supported by verified lots in collections.

***Hemibrycon jabonero*** Schultz, 1944

Endemic to the Lago de Maracaibo Basin (Lima et al. 2003). Not present in the cis-Andean drainages (Bertaco and Malbarba 2010).

***Hemibrycon metae*** Myers, 1930

Not endemic to Colombia, since it is also found in the Orinoco River Basin and Caribbean coastal basins from Venezuela (Bertaco and Malabarba 2010). Removed from the Amazon system.

***Creagrutus cochui*** Géry, 1964

Maldonado-Ocampo et al. (2008) inadvertently omitted its inclusion in the Orinoco River Basin, based on Vari and Harold (2001), who listed one record from the Guaviare River in Colombia.

***Creagrutus nigrostigmatus*** Dahl, 1960

Restricted to the Caribbean system (Harold and Vari 1994). Presence in the Magdalena-Cauca system is not supported by verified lots in collections.

***Knodus deuterodonoides*** (Eigenmann, 1914)

Restricted to the Lago de Maracaibo and Orinoco River basins (Lima et al. 2003). Presence in the Amazon River Basin is not supported by verified lots.

***Othonocheirodus***

Distribution extended to include the Amazon basin (CZUT-IC, MPUJ).

***Bryconamericus andresoi*** Román-Valencia, 2003

Known only from the upper Patia River Basin (Pacific slope) (Román-Valencia 2003a). Presence in the Magdalena-Cauca system is not supported by verified lots in collections.

***Bryconamericus guizae*** Román-Valencia, 2003

Known only from the upper Mira River Basin (Pacific slope) (Román-Valencia 2003a). Presence in the Magdalena-Cauca system is not supported by verified lots in collections.

***Astyanax integer*** Myers, 1930

Restricted to the Orinoco River Basin in Colombia and Venezuela, not being found in the Amazon Basin in Colombia (Taphorn 1993, Lima et al. 2003).

***Astyanax maximus*** (Steindachner, 1876)

Restricted in Colombia to the Amazon River Basin (Flávio C. T. Lima, pers. com.).

***Astyanax microlepis*** Eigenmann, 1913

Restricted to the upper Cauca River drainage (Madgalena-Cauca system) in Colombia (Lima et al. 2003).

***Astyanax ruberrimus*** Eigenmann, 1913

Presence in the Magdalena-Cauca system is not supported by verified lots in collections.

**Gasteropelecidae**

***Engraulisoma taeniatum*** Castro, 1981

Originally included in Ortega et al. (2006) for the Amazon basin, but without supporting records from Colombia.

**Bryconidae**

***Brycon melanopterus*** (Cope, 1871)

Restricted to the Amazon system (Lima 2017). Removed from the Orinoco system.

***Brycon rubricauda*** Steindachner, 1879

Restricted to the Magdalena-Cauca system (Lima 2003). Presence in the Caribbean system is not supported by records in collections.

***Salminus affinis*** Steindachner, 1880

Restricted to trans-Andean drainages (Lima et al. 2003). Bogotá-Gregory and Maldonado-Ocampo et al. (2006a) listed IAvH-P, where the following lots: 1801, 5801, coming from the Putumayo and Caquetá Rivers, respectively, were found to be identified as this species. However, these specimens correspond to an undescribed species, strictly Cis-Andean (Lima et al. 2013).

**Tripotheidae**

***Triportheus auritus*** (Valenciennes, 1850)

Presence in the Amazon River Basin was inadvertently omitted, based on Galvis et al (2007b).

**GYMNOTIFORMES**

**Hypopomidae**

***Brachyhypopomus brevirostris*** (Steindachner, 1868)

Restricted to cis-Andean drainages (Crampton et al. 2016). Removed from the Magdalena-Cauca system.

**Sternopygidae**

***Sternopygus aequilabiatus*** (Humboldt, 1805)

Distributed also in the Atrato River Basin, Caribbean system (Maldonado-Ocampo et al. 2013b).

**Apteronotidae**

***Apteronotus rostratus*** (Meek & Hildebrand, 1913)

Distributed also in the Magdalena and Cauca River basins (de Santana and Vari 2013).

***Sternarchella schotti*** (Steindachner, 1868)

Distributed also in the Orinoco River Basin (río Meta) (Evans et al. 2017).

***Sternarchorhynchus oxyrhynchus*** (Müller & Troschel, 1848)

Restricted to the Orinoco River Basin (de Santana and Vari 2010). Removed from the Amazon River Basin.

**SILURIFORMES**

**Trichomycteridae**

***Malacoglanis gelatinosus*** Myers & Weitzman, 1966

Distribution significantly extended to include the Orinoco River Basin, based on two specimens collected in a tributary of the Meta River drainage (CZUT-IC 13816, IAvH-P 13640).

***Megalocentor echthrus*** de Pinna, 1991

Lasso et al. (2005) listed a single Venezuelan collection (MBUCV), thus its presence in the Colombian Orinoco is not supported by lots in collections.

***Trichomycterus striatus*** (Meek & Hildebrand, 1913)

Records of the species have been corroborated only from the Magdalena-Cauca system.

***Tridensimilis venezuelae*** Schultz, 1944

Removed from the Orinoco River Basin, given that the record in Galvis et al. (2007a) corresponds to an undescribed species of the genus *Tridens* (DoNascimiento 2013).

**Callichthyidae**

***Corydoras axelrodi*** Rössel, 1962

Restricted to the Meta River drainage, Orinoco system in Colombia (Reis 2003).

***Corydoras delphax*** Nijssen & Isbrücker, 1983

Restricted to the Inírida River drainage, Orinoco system in Colombia (Castro 1987b, Reis 2003).

***Corydoras loxozonus*** Nijssen & Isbrücker, 1983

Restricted to the Guaviare River drainage, Orinoco system in Colombia (Castro 1987b, Reis 2003).

***Corydoras melanotaenia*** Regan, 1912

Restricted to the Meta River drainage, Orinoco system in Colombia (Castro 1987b, Reis 2003).

***Corydoras metae*** Eigenmann, 1914

Restricted to the Meta and Guaviare River drainages, Orinoco system in Colombia (Castro 1987b, Reis 2003).

***Corydoras simulatus*** Weitzman & Nijssen, 1970

Restricted to the upper Meta River, Orinoco system (Castro 1987b, Reis 2003).

**Astroblepidae**

***Astroblepus latidens*** Eigenmann, 1918

Maldonado-Ocampo et al. (2008) inadvertently omitted as present in the Orinoco River Basin, which encompasses its type locality.

**Loricariidae**

***Ancistrus centrolepis*** Regan, 1913

Restricted to the Pacific versant and Atrato River in Colombia (Taphorn et al. 2013). Removed from the Magdalena-Cauca system.

***Ancistrus triradiatus*** Eigenmann, 1918

Presence in the Amazon and Caribbean systems is not supported by validated lots in collections.

***Chaetostoma marginatum*** Regan, 1904

Available records in Colombian collections only come from the Pacific system.

***Chaetostoma milesi*** Fowler, 1941

Restricted to the Magdalena-Cauca system (Ballen et al. 2016a). Presence in the Orinoco River Basin was based on the long history of confusion with the recently named *Chaetostoma joropo* Ballen, Urbano-Bonilla & Maldonado-Ocampo, 2016.

***Chaetostoma platyrhynchus*** (Fowler, 1943)

Distributed also in the Orinoco River Basin (Ballen et al. 2016a).

***Chaetostoma setosus*** Boulenger, 1887

Distributed in the Cesar River drainage (Magdalena River drainage), its type locality also likely corresponds to this region (Ballen and Vari 2012).

***Cordylancistrus daguae*** (Eigenmann, 1912)

Restricted to the Pacific system (Dagua River basin) (Fisch-Muller 2003).

***Dolichancistrus cobrensis*** (Schultz, 1944)

Distributed in the Orinoco system in Colombia, based on the lot ICN-MHN 18009.

***Farlowella colombiensis*** Retzer & Page, 1997

Known only from the Meta River drainage (Retzer and Page 1997).

***Farlowella gracilis*** Retzer & Page, 1997

Known only from the Caquetá River drainage (Retzer and Page 1997).

***Farlowella oxyrryncha*** (Kner, 1853)

Maldonado-Ocampo et al. (2008) listed Lasso et al. (2005) as supporting its presence in the Orinoco. However, all collections therein cited are from Venezuela.

***Hypoptopoma steindachneri*** Boulenger, 1895

Restricted to the Amazon River Basin (Aquino and Schaefer 2010). Removed from the Orinoco River Basin.

***Hypostomus argus*** (Fowler, 1943)

Known only from the Orinoco River Basin (Ferraris 2007); also present in western tributaries of the Orinoco River in the llanos of Venezuela (Lasso et al. 2004, 2005).

***Hypostomus niceforoi*** (Fowler, 1943)

Widely distributed in the Amazon and Orinoco River basins in Colombia, and also found in the Napo River drainage (Amazon River Basin) in Ecuador (Jonathan W. Armbruster pers. com.).

***Isorineloricaria villarsi*** (Lütken, 1874)

Endemic to the Lago de Maracaibo Basin (Ray and Armbruster 2016). In Colombia it is only known from the Catatumbo River, a tributary of the Lago de Maracaibo Basin.

***Oxyropsis acutirostra*** Miranda Ribeiro, 1951

Contrary to Bogotá-Gregory and Maldonado-Ocampo (2006a) who listed Miranda Ribeiro (1951) as supporting its presence in the Colombian Amazon, this last reference does not list localities from this region.

***Pseudorinelepis genibarbis*** (Valenciennes, 1840)

Found also in the Amazon system (Ortega-Lara 2016).

***Pterygoplichthys undecimalis*** (Steindachner, 1878)

Known only from the Magdalena-Cauca system (Weber 2003) and is thus considered as endemic to this drainage.

***Rhadinoloricaria laani*** (Nijssen & Isbrücker, 1988)

Distributed only in the Orinoco River Basin, both in Colombia and Venezuela (Lasso et al. 2005, Ferraris 2007).

***Rhadinoloricaria*** ***listrorhinos*** (Nijssen & Isbrücker, 1988)

Distributed only in the Meta River drainage, Orinoco River Basin (Ferraris 2007).

***Rineloricaria formosa*** Isbrücker & Nijssen, 1979

Available records in Colombian collections come only from the Orinoco River Basin.

***Rineloricaria magdalenae*** (Steindachner, 1879)

Presence in the Pacific system is not supported by records in collections.

***Sturisomatichthys aureum*** (Steindachner, 1900)

Presence in the Pacific system is not supported by records in collections.

***Sturisomatichthys tamanae*** (Regan, 1912)

Presence in the Magdalena-Cauca and Caribbean systems is not supported by records in collections.

**Aspredinidae**

***Dupouyichthys sapito*** Schultz, 1944

Recorded from the Catatumbo River drainage (Caribbean system) (Ortega-Lara et al. 2012).

***Xyliphius lepturus*** Orcés V., 1962

Presence in the Caribbean system is not supported by references or lots.

**Auchenipteridae**

***Ageneiosus ucayalensis*** Castelnau, 1855

Distributed in the Amazon River Basin, lower Tocantins and Corantijn Rivers (Ribeiro et al. 2017).

***Ageneiosus vittatus*** Steindachner, 1908

Lasso et al. (2005) listed only Venezuelan collections to support its presence in the Orinoco River Basin. All available records in Colombian collections come from the Amazon River Basin.

***Auchenipterichthys punctatus*** (Valenciennes, 1840)

Distributed also in the Orinoco River Basin (Caño matavén): IAvH-P 10008-10019.

***Centromochlus altae*** Fowler, 1945

Distribution includes the Orinoco River Basin, based on a record from the Meta River (IAvH-P 551).

***Centromochlus reticulatus*** (Mees, 1974)

Presence in the Orinoco River Basin recorded in Galvis et al. (2007a) was inadvertently omitted in Maldonado-Ocampo et al. (2008).

***Tatia aulopygia*** (Kner, 1858)

The record from the Orinoco River Basin (IAvH-P 5634) that supports its inclusion in this basin in Maldonado-Ocampo et al. (2006a), actually corresponds to *T. strigata*. On the other hand, inclusion of *Tatia aulopygia* in Lasso et al. (2005) and Bogotá-Gregory and Maldonado-Ocampo (2006a) is not supported by specimens validated in Colombian collections. Likewise the species was not listed in Mojica et al. (2005), as supporting its presence in the Amazon system as indicated in Maldonado-Ocampo et al. (2008). Thus the presence of the species in the Amazon system is validated by the lots IAvH-P 9108 and 9134.

***Trachelyopterus fisheri*** (Eigenmann, 1916)

Presence in the Magdalena-Cauca system is not supported by references or verified lots in collections.

**Doradidae**

***Agamyxis albomaculatus*** (Peters, 1877)

Restricted to the Orinoco River Basin (Sabaj 2003).

***Amblydoras gonzalezi*** (Fernández-Yépez, 1968)

Restricted to the Orinoco River Basin (Sabaj 2003). The species pictured in Galvis et al. (2007b, figs. 338a, b, p. 431) seems to correspond instead to *Amblydoras affinis* (Kner, 1855).

***Pterodoras rivasi*** (Fernández-Yépez, 1950)

Restricted to the Orinoco River basin (Sabaj 2003). Bogotá-Gregory and Maldonado-Ocampo (2006a) listed ICN-MHN, but this collection has a single lot coming from the Orinoco River Basin.

***Tenellus leporhinus*** (Eigenmann, 1912)

Restricted in Colombia to the Orinoco River Basin (Sabaj Pérez et al. 2014).

**Heptapteridae**

***Cetopsorhamdia picklei*** Schultz, 1944

Endemic to the Lago de Maracaibo Basin. Records from the Orinoco River Basin correspond to an undescribed species, similar in general appareance to *C. picklei* (Armando Ortega-Lara pers. obs.).

***Pimelodella eutaenia*** Regan, 1913

Presence in the Magdalena-Cauca system is not supported by verified lots in collections.

**Pimelodidae**

***Hypophthalmus oremaculatus*** Nani & Fuster, 1947

Distributed in the Paraná, Amazon, and Orinoco basins (Littmann et al. 2015).

***Megalonema platycephalum*** Eigenmann, 1912

Presence in the Amazon system is not supported by records in collections. Contrary to Maldonado-Ocampo et al. (2008), the species is not listed in Mojica et al. (2005).

***Perrunichthys perruno*** Schultz, 1944

Distribution corrected to include the Caribbean system that encompasses its type locality (Lago de Maracaibo Basin). Removed from the Amazon River Basin.

***Pimelodus albofasciatus*** Mees, 1974

Present in the Amazon system (IAvH-P 256, 503, 6023, 8754, 8821, 8850, 8913).

***Pimelodus grosskopfii*** Steindachner, 1879

Presence in the Caribbean and Pacific systems is not yet supported by verified lots in collections.

***Pimelodus punctatus*** (Meek & Hildebrand, 1913)

Presence in the Pacific system is not yet supported by verified lots in collections.

**Pseudopimelodidae**

***Batrochoglanis acanthochiroides*** (Güntert, 1942)

Restricted to the Lago de Maracaibo Basin (Shibatta 2003, Armando Ortega-Lara pers. obs.), which corresponds to its type locality, and inadvertently omitted in Maldonado-Ocampo et al. (2008). Instead, the species was indicated for the Pacific system.

***Batrochoglanis raninus*** (Valenciennes, 1840)

Restricted in Colombia to the Amazon River Basin. The species found in the Orinoco River Basin corresponds to an undescribed species (Armando Ortega-Lara pers. obs.).

***Batrochoglanis transmontanus*** (Regan, 1913)

Presence in the Magdalena-Cauca system is not supported by verified lots.

***Batrochoglanis villosus*** (Eigenmann, 1912)

Bogotá-Gregory and Maldonado-Ocampo (2006a) listed USNM, but searches in the online database of this collection do not show records of this species from Colombia. In addition, similar searches in the Colombian collections failed to locate any record from the Amazon system. Contrarily all available records come from the Orinoco River Basin which was not included in Maldonado-Ocampo et al. (2008).

***Microglanis secundus*** Mees, 1974

Presence in the Caribbean system (Catatumbo River drainage) is based on a misidentified juvenile specimen of *Batrochoglanis acanthochiroides* (Güntert, 1942) (Ortega-Lara et al. 2012).

***Pseudopimelodus bufonius*** (Valenciennes, 1840)

Distributed in the cis-Andean basins of northeastern South America and in the Lago de Maracaibo Basin (Shibatta 2003). Records from the Magdalena-Cauca system actually correspond to *Pseudopimelodus schultzi* (Dahl, 1955).

***Pseudopimelodus schultzi*** (Dahl, 1955)

Restricted to the trans-Andean basins of the Caribbean and Magdalena-Cauca systems (Shibatta 2003, Armando Ortega-Lara pers. obs.). Presence in the Orinoco River Basin is erroneous.

**BATRACHOIDIFORMES**

**Batrachoididae**

***Daector quadrizonatus*** (Eigenmann, 1922)

Presence in the Pacific system is not supported by verified lots.

**CICHLIFORMES**

**Cichlidae**

***Acaronia vultuosa*** Kullander, 1989

Restricted to the Orinoco River Basin and upper Rio Negro (Kullander 2003). Inclusion in the Amazon Basin by Bogotá-Gregory and Maldonado-Ocampo (2006a) based on its original description is not valid, since all the Colombian localities therein listed correspond to the Orinoco River Basin of Colombia (Inírida and Vichada rivers).

***Aequidens metae*** Eigenmann, 1922

Known only from the Orinoco River Basin in Colombia (Kullander 2003). Removed from the Amazon system.

***Apistogramma hongsloi*** Kullander, 1979

Restricted to the Orinoco River Basin (Kullander 2003). Removed from the Amazon system.

***Bujurquina mariae*** (Eigenmann, 1922)

Restricted to the Orinoco River Basin (Kullander 2003). Removed from the Amazon system.

***Caquetaia kraussii*** (Steindachner, 1878)

Presence in the Pacific system is not supported by verified lots in collections.

***Crenicichla reticulata*** (Heckel, 1840)

Lasso et al. (2005) listed a single Venezuelan collection (MBUCV). Colombian collections lack of records from the Orinoco River Basin and instead all available records of this species come from the Amazon system. On the other hand, Maldonado-Ocampo et al. (2008) inadvertently omitted its presence in the Amazon River Basin based on Kullander (1986).

**CYPRINODONTIFORMES**

**Cynolebiidae**

*Cynodonichthys elegans* (Steindachner, 1880)

Originally described from the Cauca River drainage, its distribution is restricted here to the Magdalena-Cauca system, discarding its presence in the cis-Andean Orinoco Basin.

Poeciliidae

*Fluviphylax pygmaeus* (Myers & Carvalho, 1955)

Distributed also in the Orinoco River Basin (Inírida River) based on the lot NRM 26248.

*Gambusia lemaitrei* Fowler, 1950

Originally described from Ciénaga del Totumo (Totumo Lake), which is part of the Caribbean system and not from the Magdalena-Cauca as implied in Maldonado-Ocampo et al. (2008).

*Priapichthys caliensis* (Eigenmann & Henn, 1916)

Presence in the Pacific system is not supported by records in Colombian collections (Maldonado-Ocampo et al. 2013b).
