# Supplementary material for: Checklist of the freshwater fishes of Colombia: a Darwin Core alternative to the updating problem
Source: Zookeys. 2017 Oct 13;(708):25–138. doi: 10.3897/zookeys.708.13897 (PMC5674168; doi:10.3897/zookeys.708.13897)
Supplement: Supplementary material 5 — Species deleted from the Colombian continental ichthyofauna [file zookeys-708-025-s005.docx]

SUPPLEMENTARY FILE 5

Species deleted from the Colombian continental ichthyofauna.

**Myliobatiformes**

**Potamotrygonidae**

***Potamotrygon castexi*** Castello & Yagolkowski, 1969

Junior synonym of *Potamotrygon falkneri* Castex & Maciel, 1963, which is distributed in the basins of the Paraná-Paraguay River, La Plata River, and upper basin of the Amazon river in Bolivia, Peru, and Brazil (de Silva and Carvalho 2011).

***Potamotrygon hystrix*** (Müller & Henle, 1834)

Restricted to the Parana-Paraguay River Basin and La Plata River (Lasso et al. 2014).

**Clupeiformes**

**Engraulidae**

***Lycengraulis grossidens*** (Spix & Agassiz, 1829)

Not listed in Maldonado-Ocampo (2004) neither in Maldonado-Ocampo et al. (2006a), which were cited in Maldonado-Ocampo et al. (2008) as supporting references for the inclusion of this species. The other reference provided (Lasso et al. 2005) only list this species for the Orinoco delta and consequently the collections cited therein are from Venezuela (MBUCV, MHNLS).

**Characiformes**

**Crenuchidae**

***Characidium fasciatum*** Reinhardt, 1867

Distributed in Das Velhas River of the São Francisco River Basin and Paranaíba River, Grande River and Tietê River of the upper Paraná River Basin (Buckup 2003). The populations recorded from Colombian trans-Andean systems correspond to undescribed species and are informally cited as *Characidium* gr. *fasciatum* (Paulo Andreas Buckup pers. com.), while the cis-Andean populations correspond to *C. zebra* Eigenmann, 1909 (Buckup, 1992).

***Melanocharacidium depressum*** Buckup, 1993

Bogotá-Gregory and Maldonado-Ocampo (2006a) listed Buckup (1993) as a supporting reference for the presence of the species in the Colombian Amazon, however no Colombian localities are listed for this species in Buckup (1993).

**Eythrinidae**

***Hoplias microlepis*** (Günther, 1864)

We opt to remove this species given its apparent absence in the Colombian Pacific slope (Mattox et al. 2014) is supplemented by the observation of the lack of records in the Colombian collections accessed.

**Serrasalmidae**

***Metynnis maculatus*** (Kner, 1858)

Based only on CP-IIAP (Ortega et al. 2006), corresponding to collections in the Peruvian part of the Putumayo River, Amazon system. Confirmed records are lacking in Colombian collections.

***Mylesinus schomburgkii*** Valenciennes, 1850

Lasso et al. (2005) does not list Colombian collections. Recent searching attempts in IAvH-P or ICN-MHN, the two main Colombian collections consulted by Lasso et al. (2005), failed to located any lot of this species. This nominal species has been frequently confused with the similar named *Myloplus schomburgkii* (Jardine, 1841), a very different looking species, characterized by a conspicuous dark vertical band crossing the side of the body.

***Myleus pacu*** (Jardine & Schomburgk, 1841)

*Species inquirenda* as *Myletes pacu* Jardine & Schomburgk in Schomburgk, 1841, and described from the Essequibo River in Guyana (Jégu 2003). The single record of this species for Colombia (Bejarano and Blanco 2001, cited in Bogotá-Gregory and Maldonado-Ocampo 2006) probably consists in a misidentified Amazonian species of serrasalmine.

***Myleus ternetzi*** (Norman, 1929)

Supposedly listed in Galvis et al. (2007b) according to Maldonado-Ocampo et al. (2008), but it was not included in that work, leaving the presence status of the species in Colombia as uncertain. In addition, the species is distributed in the rivers of the east and northeast of the Guiana shield (Jégu 2003), therefore its presence in Colombia is doubtful.

**Hemiodontidae**

***Hemiodus goeldii*** Steindachner, 1908

Restricted to the Amapá River Basin in Brasil (Langeani 2003). The Amazon record (IAvH-P 2389) that supports this species in Bogotá Gregory and Maldonado-Ocampo (2005), corresponds to *Hemiodus atranalis* (Fowler, 1940), while the Orinoco record (IAvH-P 4060) in Maldonado-Ocampo et al. (2006a) corresponds to *H. semitaeniatus* Kner, 1858.

**Anostomidae**

***Leporinus arcus*** Eigenmann, 1912

Lasso et al. (2005) only listed Venezuelan collections. Not supported by lots in collections.

***Leporinus desmotes*** Fowler 1914

Distributed in Essequibo and Tocantins rivers (Burns et al. 2017). Populations from the Orinoco River Basin correspond to *Leporinus enyae* Burns, Chatfield, Birindelli & Sidlauskas 2017.

***Leporinus leschenaulti*** Valenciennes, 1850

Distributed in coastal rivers of French Guiana and northern Brazil (Garavello and Britski 2003). Inclusion of this species in the unpublished dissertation of Bejarano and Blanco (2001) was probably based on the lot ICN-MHN 4252, coming from the Caquetá, and identified by I. Bejarano. However this identification is unreliable. The presence of this species in the Colombian Orinoco River Basin (Maldonado-Ocampo et al. 2006a) is also doubtful, since the species has not been recorded anywhere in the basin (Lasso et al. 2005).

***Leporinus maculatus*** Müller & Troschel, 1844

Distributed in the coastal rivers of the Guianas and in the São Francisco River Basin (Garavello and Britski 2003). The lot IAvH-1822 which is the basis for the inclusion of the species in Bogotá-Gregory and Maldonado-Ocampo (2006a) corresponds to *Leporinus parae* Eigenmann, 1907.

***Leporinus melanopleura*** Günther, 1864

Recorded only for the Cipó River Basin in the Brazilian state of Bahia (Garavello and Britski 2003). The lot IAvH-P 4016 that supports this species in Maldonado-Ocampo et al. (2006a) actually corresponds to *Leporinus brunneus* Myers, 1950.

***Leporinus melanostictus*** Norman, 1926

Distributed in the Approuague River and Oyapock River basins (Brazil and French Guiana). Originally listed in Mojica (1999) and probably based on misidentifications of other *Leporinus* species occurring in the Amazon Basin.

***Leporinus octofasciatus*** Steindachner, 1915

Distributed in the Cubatão River in the Brazilian state of Santa Catarina and in the upper Paraná River Basin (Garavello and Britski 2003). Originally listed in Mojica (1999) and probably based on misidentifications of other *Leporinus* species occurring in the Amazon Basin.

***Leporinus piau*** Fowler, 1941

Recorded only for the Salgado River, a coastal Atlantic river of Ceará state in northeastern Brazil (Garavello and Britski 2003). The two references listing this species for the Colombian Amazon (Santos 2000 and Vejarano 2000, cited in Bogotá-Gregory and Maldonado-Ocampo 2006) are probably based on misidentifications of other species occurring in this river basin.

***Leporinus steyermarki*** Inger, 1956

The lot IAvH-P 2368, which is the base for the inclusion of the species in the Colombian Amazon (Bogotá-Gregory and Maldonado-Ocampo, 2005), actually corresponds to *Leporinus subniger* Fowler, 1943, while the single lot from the Orinoco River Basin (IAvH-P 2726), inferred to be the basis for the species inclusion in the Colombian Orinoco by Lasso et al. (2005), is in fact *Leporinus boehlkei* Garavello, 1988.

***Petulanos spiloclistron*** (Winterbottom, 1974)

Restricted to the Nickerie River in Suriname (Garavello and Britski 2003). Originally listed in Mojica (1999) but not in Mojica et al. (2005) nor Ortega et al. (2006), as indicated in Maldonado-Ocampo et al. (2008). The record in Mojica (1999) as *Anostomus spiloclistron* is probably based on a misidentification.

***Pseudanos gracilis*** Myers, 1930

Distributed in the middle basin of the Amazon, including the Negro, Branco, Uatumã and Madeira rivers (Birindelli et al. 2012). The single record supporting its presence in the Amazon River Basin in Colombia (ICN-MHN 7190) in Mojica et al. (2005) and illustrated in Galvis et al. (2006: fig. 32, p. 133; pl. 13a, p. 438) corresponds to a juvenile of *Schizodon fasciatus* Spix & Agassiz, 1829 (Flávio C. T. Lima, pers. com.).

***Pseudanos irinae*** Winterbottom, 1980

Junior synonym of *Pseudanos trimaculatus* (Kner, 1858) (Birindelli et al. 2012).

***Schizodon dissimilis*** (Garman, 1890)

Restricted to the Poti River Basin in the state of Piauí, Brazil (Garavello and Britski 2003).

***Synaptolaemus cingulatus*** (Myers & Fernández-Yépez, 1950)

Junior synonym of *Synaptolaemus latofasciatus* (Steindachner, 1910) (Britski et al. 2011).

**Chilodontidae**

***Caenotropus maculosus*** (Eigenmann, 1912)

Restricted to the Essequibo River Basin in Guyana and its tributary the Cuyuní River in Venezuela, Corantijn River in Surinam and Guyana, and Marowijne-Maroni River in Surinam and French Guiana (Vari and Raredon 2003). The two lots from the Tomo River, Orinoco system (IAvH-P 4098 and 4101) cited in Maldonado-Ocampo et al. (2006a) actually correspond to *Caenotropus mestomorgmatos* Vari, Castro & Raredon, 1995. On the other hand, Ortega et al. (2006) listed ICN-MHN as supporting the presence of the species in the Colombian side of the Putumayo River, but this collection lacks of records of this species.

**Curimatidae**

***Cyphocharax gillii*** (Eigenmann & Kennedy, 1903)

Distributed in the Paraguay River Basin in Brazil and Paraguay (Vari 2003). The single record from the Colombian Amazon (Calderón and Hincapié 2001 cited in Bogotá-Gregory and Maldonado-Ocampo 2006a) is probably based on the misidentification of another species occurring in the Amazon Basin.

***Cyphocharax*** ***stilbolepis*** Vari, 1992

Recorded only from the Tocantins and Xingú River basins in Brazil (Vari 2003). Listed for the Colombian Amazon in Bogotá-Gregory and Maldonado-Ocampo (2006a) and Maldonado-Ocampo et al. (2008), but lacking of taxonomically validated records in collections.

**Lebiasinidae**

***Lebiasina aureoguttata*** (Fowler, 1911)

Listed for the Mira River (Mojica et al. 2004) and San Juan River (Maldonado-Ocampo et al. 2013b). However, the last authors recognized that there are no confirmed records in Colombian collections.

***Lebiasina pleurotaenia*** (Regan, 1903)

Junior synonym of *Lebiasina erythrinoides* (Valenciennes, 1850) (as *Piabucina erythrinoides* in Taphorn and Lilyestrom 1980).

***Copeina osgoodi*** Eigenmann, 1922

No confimed records in Colombian collections. The only apparent record of the species is in UF (Bogotá-Gregory and Maldonado-Ocampo 2006a), although it is not indicated the corresponding catalog number of the lot. Besides, the species was not listed in Mojica *et al*. (2005) provided as a support reference in Maldonado-Ocampo et al. (2008).

***Copella metae*** (Eigenmann, 1914)

*Copella metae* is treated as a junior synonym of *Copella eigenmanni* (Regan, 1912) in Zarske (2011). This last nominal species was considered as a junior synonym of *Copella arnoldi* (Regan, 1912) (Mol et al. 2012), based on a personal communication of Manoela Marinho, but Marinho (2014) retracted this synonymy proposal and treated *C. eigenmanni* as a valid species.

***Pyrrhulina stoli*** Boeseman, 1953

The taxonomic identity of the lot IAvH-P 4997, listed in Maldonado-Ocampo et al. (2008), as supporting the presence of the species in the Orinoco River Basin, actually corresponds to *Pyrrhulina* cf. *eleanorae*. Inclusion in the Caquetá River drainage, Amazon system by Castro and Arboleda (1988) is probably based on a misidentification.

***Nannostomus espei*** (Meinken, 1956)

Known only for the Mazaruni River in Guyana (Weitzman and Weitzman 2003). No confirmed records in Colombian collections.

**Acestrorhynchidae**

***Acestrorhynchus lacustris*** (Lütken, 1875)

Distributed in the São Francisco River Basin and upper basin of the Paraná River (Menezes 2003). The lots identified as this species (ICN-MHN 3864, ICN-MHN 5130, ICN-MHN 6189, ICN-MHN 6269, ICN-MHN 6541, ICN-MHN 6565, ICN-MHN 6959, ICN-MHN 7101), and listed in Arbeláez et al. (2004), Mojica et al. (2005), and Galvis et al. (2007b), and also illustrated in the latter reference (fig. 77, p. 182; pl. 29c, p. 454), actually correspond to *Acestrorhynchus falcatus* (Bloch, 1794), a species broadly distributed in the Amazon and Orinoco River basins and rivers of northern South America (Menezes 2003). Distinguished from *A. lacustris* by having a vertically elongated humeral mark (Toledo-Piza 2007), shaped as an inverted teardrop and occupying at least one third of the body height (López-Fernández and Winemiller 2003).

**Characidae**

***Acanthocharax microlepis*** Eigenmann, 1912

Specimen illustrated in Galvis et al. (2007a: fig. 168, p. 333) and forming part of the lot ICN-MHN 12531 is in fact *Heterocharax macrolepis* Eigenmann, 1912 (Flávio C. T. Lima, pers. com.).

***Charax gibbosus*** (Linnaeus, 1758)

Restricted to the Essequibo River, coastal rivers of Surinam and Guyana, and Surumu River, a tributary of the Branco River in Brazil (Menezes and Lucena 2014).

***Charax leticiae*** Lucena, 1987

Distributed in the Tocantins, Araguaia, Paraguay and Aripuanã River basins (Menezes and Lucena 2014).

***Phenacogaster megalosticta*** Eigenmann, 1909

Restricted to the Essequibo River in Guyana (Lucena and Malabarba 2010).

***Phenacogaster microsticta*** Eigenmann, 1909

Distributed in the Essequibo River and Demerara River basins in Guyana and Suriname, respectively (Lucena and Menezes 2003). Listed for Colombia in Lasso et al. (2005), but no reference lots are indicated in this work. The basis for its inclusion in the Colombian Orinoco is probably based on misidentifications of other *Phenacogaster* species ocurrying in this system (e.g. *P. maculoblonga*, *P. prolata*).

***Deuterodon potaroensis*** Eigenmann, 1909

The lot IAvH-P 3295 upon which this record was based corresponds to *Knodus deuterodonoides* (Eigenmann, 1914).

***Hemigrammus brevis*** Ellis, 1911

Restricted to the São Francisco River Basin in Brazil (Lima et al. 2003).

***Hemigrammus cylindricus*** Durbin, 1909

The single supporting record is the lot IAvH-P 2555 from the río Inírida (Orinoco system), which corresponds to a different *Hemigramus* species, close to *H. microstomus* Durbin, 1918, though differing by lacking maxillary teeth.

***Hemigrammus elegans*** (Steindachner, 1882)

Records of this species in the Colombian Orinoco basin correspond to *Hyphessobrycon dorsalis* Zarske, 2014 (Flávio C. T. Lima pers. com.).

***Hemigrammus erythrozonus*** Durbin, 1909

Known only from the Essequibo River Basin in Guyana (Lima et al. 2003). Record based on Mojica et al. (2005), which consists in the lots ICN-MHN 10478 and ICN-MHN 10549, and illustrated in Galvis et al. (2006: fig. c, pl. 38, p. 463) is a misidentification (Flávio C. T. Lima pers comm.).

***Hemigrammus guyanensis*** Géry, 1959

Known only from the Maroni, Mana, Approuague, and Oyapock Rivers in French Guiana (Lima et al. 2003) and northeastern Brazil (Flávio C. T. Lima, pers. comm.). Records from other areas are likely incorrect.

***Hemigrammus iota*** Durbin, 1909

Known only from the Essequibo River Basin in Guyana (Lima et al. 2003). Inclusion of this species in Bogotá-Gregory and Maldonado-Ocampo (2006a) is based on the unpublished dissertation of Bejarano and Blanco (2001) and probably consists in a misidentification.

***Hemigrammus marginatus*** Ellis, 1911

Restricted to rivers from northeastern Brazil (including the rio São Francisco), and the upper rio Paraná (Ota et al. 2015). Records of this species from the Amazon River basin seem to be based on misidentifications of *Hyphessobrycon diancistrus* Weitzman, 1977, while specimens from the Orinoco River Basin correspond to *H. otrynus* Benine & Lopes, 2008 (Flávio C. T. Lima pers. com.).

***Hemigrammus mimus*** Böhlke, 1955

Records of this species in examined Colombian collections corresponds to *Cyanogaster noctivaga* Mattox, Britz, Toledo-Piza & Marinho, 2013 (Flávio C. T. Lima pers. com.).

***Hemigrammus tridens*** Eigenmann, 1907

Endemic from the Paraguay River Basin (Ota, 2010). The lot IAvH-P 2419 listed in Bogotá-Gregory and Maldonado-Ocampo (2005) as the single basis for inclusion of *Hemigrammus tridens* in the Colombian Amazon basin (Bogotá-Gregory and Maldonado-Ocampo 2006a), actually corresponds to two species of undetermined taxonomic status, due to the badly preservation of the specimens (Flávio C. T. Lima pers. com.).

***Hyphessobrycon albolineatum*** Fernández-Yépez, 1950

Maldonado-Ocampo et al. (2008) listed this species in the Orinoco basin, based on the lot IAvH-P 760 which actually corresponds to *Hemigrammus microstomus* Durbin, 1908 (Flávio C. T. Lima pers. com.). Besides taxonomic status of this nominal species is based on a spurious identification (Flávio C. T. Lima pers. com.).

***Hyphessobrycon ecuadoriensis*** Eigenmann & Henn, 1914

Known only from the type locality in Ecuador (Lima et al. 2003).

***Hyphessobrycon eos*** Durbin, 1909

Known only from Guyana (Lima et al. 2003).

***Hyphessobrycon eques*** (Steindachner, 1882)

Specimen identified as this species (Galvis et al. 2007b: fig. 147, p. 382) corresponds to *Hyphessobrycon bentosi* Durbin, 1908 (Flávio C. T. Lima pers.com.).

***Hyphessobrycon inconstans*** (Eigenmann & Ogle, 1907)

Known only from the type locality in Pará, Brazil (Lima et al. 2003).

***Hyphessobrycon minor*** Durbin, 1909

Lasso et al. (2005) listed a single Venezuelan collection (MBUCV), while the other two references accounted by Maldonado-Ocampo et al. (2008): Maldonado-Ocampo (2004) and Maldonado-Ocampo et al. (2006a), do not list this species.

***Hyphessobrycon panamensis*** Durbin, 1908

Specimens identified as this species in Colombia correspond to *Hyphessobrycon sebastiani* García-Alzate, Román-Valencia & Taphorn, 2010 according to García-Alzate et al. (2013).

***Hyphessobrycon scholzei*** Ahl, 1937

Contrary to what is proposed by Bogotá-Gregory and Maldonado-Ocampo (2006a) with the inclusion of ICN-MHN, this collection has no records of this species. The other supporting source provided consists in a bachelor’s dissertation (Calderón and Hincapié 2001), which by its unpublished condition is treated here as an unreliable record, given that there are no verified lots of this species in the accessed Colombian collections.

***Hyphessobrycon tropis*** Géry, 1963

Distributed in the middle drainage of the Negro River, Brazil (Lima et al. 2003).

***Moenkhausia browni*** Eigenmann, 1909

Endemic from the Potaro River drainage in Guyana (Lima et al. 2003). Specimen illustrated in Galvis et al. (2007b: fig. 154, p. 383) correspond to an undetermined species (Flávio C. T. Lima pers. com.).

***Moenkhausia doceana*** (Steindachner, 1877)

Distributed in the Doce and Mucuri river basins, Brazil (Lima et al. 2003).

***Moenkhausia georgiae*** Géry, 1965

Lasso et al. (2005) only listed Venezuelan collections supporting its presence in the Orinoco, however records corresponding to Colombian localities are lacking.

***Moenkhausia miangi*** Steindachner, 1915

Distributed in the Branco River drainage and upper drainage of the Caroní River of the Orinoco river system (Lima et al. 2003). The lot IAvH-P 2367 from the Putumayo River drainage (Amazon system) that constitutes the basis of this species record in Bogotá-Gregory and Maldonado-Ocampo (2005, 2006a), corresponds to *Moenkhausia melogramma* Eigenmann, 1908.

***Moenkhausia naponis*** Böhlke, 1958

ICN-MHN lots listed in Arbeláez et al. (2004), Mojica et al. (2005), Galvis et al. (2006, 2007b), and specimens illustrated in Galvis et al. (2006: fig. 114, p. 222; pl. 43c, p. 468) and Galvis et al. (2007b: fig. 161, p. 385), actually correspond to *Moenkhausia chrysargyrea* (Günther, 1864) (Flávio C. T. Lima pers. com.). Besides *M. naponis* is distributed in piedmont rivers, while ICN-MHN lots are from lowland localities.

***Moenkhausia newtoni*** Travassos, 1964

Known only from the Amazon River Basin in Pará State, Brazil (Lima et al. 2003). The single lot supporting this record in Bogotá-Gregory and Maldonado-Ocampo (2005), IAvH-P2362, actually corresponds to two mixed species, *Moenkhausia oligolepis* (Günther, 1864), and an undetermined *Jupiaba* species (recataloged at IAvH-P 16004), which is close in pigmentation pattern and meristics to *J. abramoides* (Eigenmann, 1909), but have a slender body.

***Moenkhausia ovalis*** (Günther, 1868)

Taxonomic status of nominal species uncertain (Flávio C. T. Lima pers. com.).

***Moenkhausia sanctaefilomenae*** (Steindachner, 1907)

Distributed in the Parnaíba, São Francisco, upper Parana, Paraguay, and Uruguay River basins (Lima et al. 2003). The single record identified as this species in IAvH-P and coming from the Putumayo River drainage (IAvH-P 3167), actually corresponds to *Moenkhausia cotinho* Eigenmann, 1908, so it could be expected that those records supporting its inclusion in Mojica (1999) and Arbeláez et al. (2004) are also misidentifications.

***Moenkhausia takasei*** Géry, 1964

Ortega et al. (2006) only listed MUSM, accounting only for the Peruvian localities of the Putumayo River, while in the Colombian side, there are no records in the accessed collections.

***Pristella maxillaris*** (Ulrey, 1894)

Inclusion in Lasso et al. (2005) is not supported by lots available in Colombian collections and coming from the Colombian Orinoco (Atabapo and Meta rivers).

***Thayeria boehlkei*** Weitzman, 1957

Restricted to Brazil. Specimens from Colombian Amazon identified as *Thayeria obliqua* Eigenmann, 1908 correspond to an undescribed species (Cristiano R. Moreira and Flávio C. T. Lima, work in progress).

***Leptagoniates steindachneri*** Boulenger, 1887

Inclusion in Ortega *et al*. (2006) is not supported by verified lots in collections.

***Aphyodite grammica*** Eigenmann, 1912

Known only from the Essequibo River Basin (Lima et al. 2003), and has not even been recorded in Venezuela (Lasso et al. 2004). The source for its inclusion in Bogotá-Gregory and Maldonado-Ocampo (2006a) is based on unverified lots in FMNH.

***Hemibrycon orcesi*** Böhlke, 1958

The lot IAvH-P 6187 identified as this species in Bogotá-Gregory and Maldonado-Ocampo (2006a) actually corresponds to an undetermined species of *Astyanax* (Vinicius A. Bertaco pers. com.).

***Creagrutus beni*** Eigenmann, 1911

Recorded only for the upper Madeira River drainage in northeastern Bolivia (Lima et al. 2003). Inclusion in Mojica (1999) is probably based on misidentified specimens of species occurring in the Amazon Basin and later described as new in Vari and Harold (2001).

***Creagrutus magoi*** Vari & Harold, 2001

Originally listed in Maldonado-Ocampo et al. (2006a), but the collections cited therein are from Venezuela (MBUCV, MCNG).

***Creagrutus melanzonus*** Eigenmann, 1909

Known only from the Guiana region, from the Cuyuní River drainage in Venezuela to the Sinnamary River Basin in French Guiana (Lima et al. 2003). In Venezuela is restricted to the Cuyuní River, a tributary of the Essequibo River (Lasso et al. 2004). Inclusion of this species for Colombia was based on the misidentification of 10 specimens comprising the lot IAvH-P 3233, and coming from the Meta River drainage (Orinoco system), which were here reidentified as *Creagrutus* cf. *taphorni*. Albeit these specimens broadly agree in the characters provided in the diagnosis of *C. taphorni* by Vari and Harold (2001), differ in the fewer number of gill rakers on the lower limb of the first gill arch (9 vs. 13-14) and by the absence of a conspicuous humeral mark (vs. conspicuous and well defined). Therefore verification of the taxonomic status of this population is pending for the analysis of additional samples.

***Knodus caquetae*** Fowler, 1945

Junior synonym of *Bryconamericus hypopterus* Fowler, 1943 (Román-Valencia 2003b).

***Bryconamericus pachacuti*** Eigenmann, 1927

Not included in the most recent taxonomic revision of the genus in Colombia (Román-Valencia et al. 2008).

***Astyanax abramis*** (Jenyns, 1842)

Restricted to southeastern South America. Records from Colombia refer to another species of the *Astyanax bimaculatus* group (Flávio C. T. Lima, pers. com.).

***Astyanax cordovae*** (Günther, 1880)

Restricted to the Primero River Basin in Argentina (Lima et al. 2003).

***Astyanax schubarti*** Britski, 1964

Distributed in the upper basin of the Parana River in Brazil (Lima et al. 2003).

***Astyanax validus*** Géry, Planquette & Le Bail, 1991

Recorded only from the French Guiana (Lima et al. 2003).

***Ctenobrycon hauxwellianus*** (Cope, 1870)

Junior synonym of *Ctenobrycon spilurus* (Valenciennes, 1850) (Benine et al. 2010).

***Jupiaba mucronata*** (Eigenmann, 1909)

Lasso et al. (2005) listed a single Venezuelan collection (MBUCV) supporting its presence in the Orinoco, however records corresponding to Colombian localities are lacking.

***Jupiaba pinnata*** (Eigenmann, 1909)

Lasso et al. (2005) only listed Venezuelan collections supporting its presence in the Orinoco, however records corresponding to Colombian localities are lacking.

***Jupiaba poekotero*** Zanata & Lima, 2005

Bogotá-Gregory and Maldonado-Ocampo (2006a) listed its original description, however there are no Colombian localities cited in Zanata and Lima (2005).

***Serrabrycon magoi*** Vari, 1986

Inclusion in Lasso et al. (2005) is not supported by lots available in Colombian collections, besides, the species was not listed in Maldonado-Ocampo (2004).

**Bryconidae**

***Brycon bicolor*** Pellegrin, 1909

Junior synonym of *Brycon falcatus* Müller & Troschel, 1844 (Lima 2017).

***Brycon cephalus*** (Günther, 1869)

Junior synonym of *Brycon amazonicus* (Agassiz, 1829) (Lima 2017).

***Brycon opalinus*** (Cuvier, 1819)

Restricted to headwater tributaries from the rio Paraíba do Sul and rio Doce basins, eastern Brazil (Lima 2017). The single lot identified as this species in IAvH-P and coming from the Caquetá River (IAvH-P 2063) actually corresponds to *Brycon pesu* Müller & Troschel, 1845 (identified by Flávio C. T. Lima, 2015), hence is expected that its inclusion in Bogotá-Gregory and Maldonado-Ocampo (2006a), is equally based on misidentifications resulting from the use of Gery’s identification key (1977).

**Triportheidae**

***Clupeacharax anchoveoides*** Pearson, 1924

Ortega et al. (2006) only listed MUSM. Confirmed records are lacking in Colombian collections.

***Triportheus elongatus*** (Günther, 1864)

Junior synonym of *Triportheus auritus* (Valenciennes, 1850) (Malabarba 2004).

**Iguanodectidae**

***Bryconops melanurus*** (Bloch, 1794)

Known only from the coastal rivers of the Guiana shield (Lima et al. 2003), to the east of the Essequibo River Basin to the Oyapock River in the border of French Guiana and Brazil (Chernoff et al. 1994). The only three records from the Orinoco River Basin in IAvH-P, correspond to *Bryconops giacopinii* (Fernández-Yépez, 1950). Additionally, from the figures provided in Galvis et al. (2007b: fig. 88, p. 192; pl. 33b. p. 458), which are based on the records included in Mojica et al. (2005): ICN-MHN 4999, 5140, 6298, 6400, 6535, 6728, 6890, 6978, 7031, 7097, 7227, 7228, 7229 y 7230, the species illustrated seems to corresponds to an undescribed species closer to *Bryconops* sp. cf. *melanurus*, from the Brazilian Pantanal, and studied by Sidlauskas et al. (2006). This species is recognized by having the caudal-fin stripe extending up onto the caudal-fin dorsal lobe, while the stripe is completely horizontal and extends only along the central rays of the caudal fin in *B. melanurus* (Chernoff et al. 1994, fig. 2, p. 241).

**GYMNOTIFORMES**

**Gymnotidae**

***Gymnotus arapaima*** Albert & Crampton, 2001

Maldonado-Ocampo et al. (2008) listed IAvH-P as support for its inclusion, but this collection does not have specimens of this species.

***Gymnotus curupira*** Crampton, Thorsen & Albert, 2005

Maldonado-Ocampo et al. (2008) listed IAvH-P as support for its inclusion, but this collection does not have specimens of this species.

**Hypopomidae**

***Brachyhypopomus pinnicaudatus*** (Hopkins, 1991)

Distributed only in Bolivia, Brazil, Peru, and French Guiana (Crampton et al. 2016).

***Hypopomus artedi*** (Kaup, 1856)

Distributed exclusively in the Guianas region (Albert and Crampton 2003).

**Rhamphichthyidae**

***Hypopygus*** ***cryptogenes*** (Triques, 1997)

Distributed in small terra firme streams and rivers of the middle and lower basin of the Negro River and in the Preto da Eva River of the Amazon River Basin in Brasil (de Santana and Crampton 2011).

**Apteronotidae**

***Apteronotus leptorhynchus*** (Ellis, 1912)

Known only from the Essequibo River in Guyana (de Santana and Vari 2013).

***Sternarchorhynchus curvirostris*** (Boulenger, 1887)

Known only from the Bobonaza River in western Ecuador and perhaps from north-eastern Peru (de Santana and Vari 2010). The single lot supporting its inclusion in the Orinoco River Basin (IAvH-P 2981 in Maldonado-Ocampo and Albert 2003) actually corresponds to *Sternarchorhynchus roseni* Mago-Leccia, 1994 (reidentified by Carlos David de Santana, 2016).

***Sternarchella sima*** Starks, 1913

Distributed in the Amazon River Basin (Evans et al. 2017).

**SILURIFORMES**

**Trichomycteridae**

***Ituglanis amazonicus*** (Steindachner, 1882)

Distributed in the Amazon River Basin in Brasil (de Pinna and Wosiacki 2003, de Pinna and Keith 2003). The species was not listed in Lasso et al. 2005 for the Orinoco, being absent from this basin (Carlos DoNascimiento, pers. obs.).

***Trichomycterus venulosus*** (Steindachner, 1915)

Junior synonym of *Eremophilus mutisii* Humboldt, 1805 (DoNascimiento et al. 2014b).

***Tridens melanops*** Eigenmann & Eigenmann, 1889

The IAvH-P lots from the Amazon Basin and cited in Bogotá-Gregory and Maldonado-Ocampo (2006a) correspond to an undescribed species of the genus, which was analyzed and diagnosed in a phylogenetic framework in DoNascimiento (2013). Identity of the USNM lots also cited by Bogotá-Gregory and Maldonado-Ocampo (2006a) has not been verified, thus the presence of this nominal species in Colombia is not confirmed.

***Tridensimilis brevis*** (Eigenmann & Eigenmann, 1889)

The lots ICN-MHN 10101 and 10330 forming the basis for the inclusion of the species in Mojica et al. (2005), correspond to an undescribed species of the genus *Tridens*, characterized by lacking pelvic fins, also phylogenetically analyzed and diagnosed in DoNascimiento (2013).

***Plectrochilus machadoi*** Miranda Ribeiro, 1917

The lots ICN-MHN 6403 and 6709 that constitute the basis for its inclusion in Mojica et al. (2005) and Bogotá-Gregory and Maldonado-Ocampo (2006a) are cited also in Galvis et al. (2007b), who provided an illustration (Fig. 174, p. 316) and a photo (pl. 84b, p. 509), leaving no doubt that the specimens are in fact *Vandellia cirrhosa* Valenciennes, 1846.

***Plectrochilus wieneri*** (Pellegrin, 1909)

The record of this species is based on the lot ICN-MHN 6404 (Mojica et al. 2005, Bogotá-Gregory and Maldonado-Ocampo 2006a), which is also cited and illustrated in Galvis et al. (2007b: fig. 175, p. 317; pl. 84c, p. 509). From the picture in p. 509 we can confidently identify that specimen as an undescribed genus and species of Vandelliinae (Mário C. C. de Pinna pers. com., DoNascimiento 2015).

**Callichthyidae**

***Corydoras armatus*** (Günther, 1868)

Not listed in Galvis et al. (2007b), the single source provided by Maldonado-Ocampo et al. (2008) to support its inclusion in the checklist.

***Corydoras bondi*** Gosline, 1940

There are no records supporting its presence in Colombia in Lasso et al. (2005).

***Corydoras napoensis*** Nijssen & Isbrücker, 1986

The lot MM 1029 that supports this record in Castro (1987b), actually corresponds to *Corydoras elegans* Steindachner, 1876 and is now cataloged as IAvH-P 5964.

**Astroblepidae**

***Astroblepus cyclopus*** (Humboldt, 1805)

Described from the Esmeraldas River Basin in the Ecuadorian Pacific (Schaefer 2003).

***Astroblepus longifilis*** (Steindachner, 1882)

Described from the upper basin of the Hualaga River in Perú (Schaefer 2003), which is a tributary of the Amazon River Basin. Records in Colombia mainly come from the trans-Andean systems of the Cauca-Magdalena system and the Pacific versant rivers. In particular IAvH-P records correspond to localities of the Magdalena River drainage. One of these records is assignable to *Astroblepus* marmoratus (Regan, 1904) (IAvH-P 5591), while the remaining three records correspond to two undetermined species (species 1: IAvH-P 5609-5610; species 2: IAvH-P 10929). These two species do not agree with the morphological pattern of the described species from the Magdalena-Cauca system, and at least one of them (species 2) ostensibly differs in the adipose-fin shape from that described for *A. longifilis*. The lot IAvH-P 4612 corresponds to an undetermined species of the genus. Therefore, the actual taxonomic identity of the remaining lots assigned to *A. longifilis* in Maldonado-Ocampo *et al*. (2005), Mojica *et al*. (2006a), Ortega-Lara *et al*. (2006), and Villa Navarro *et al*. (2006) is pending of confirmation: CZUT-IC 318, CZUT-IC 335, CZUT-IC 351, CZUT-IC 377, CZUT-IC 413, CZUT-IC 432, CZUT-IC 438, CZUT-IC 460, CZUT-IC 1153, CZUT-IC 1166, CZUT-IC 1315, CZUT-IC 1399, CZUT-IC 1406. ICN-MHN 1843, ICN-MHN 2093-2098, ICN-MHN 2100, ICN-MHN 2102-2104, ICN-MHN 2290, ICN-MHN 2299-2300, ICN-MHN 7706, IMCN 267, IMCN 3246, IMCN 3294, IMCN 3305, MLS 411, MLS 413-414, MLS 416, MLS 419-420, MLS 431, MLS 434, MLS 437, MLS 439, MLS 446, MLS 456.

***Astroblepus rosei*** Eigenmann, 1922

Distributed in the Pacific rivers in Cajamarca, Peru (Schaefer 2003). The only record of IAvH-P identified as this species actually corresponds to *Astroblepus marmoratus* (IAvH-P 517). Therefore, the actual taxonomic identity of the remaining lots assigned to *A. rosei* in Maldonado-Ocampo et al. (2005) is pending of confirmation: IMCN 268, IMCN 312, ICNMHN 2294, MLS 417-418, MLS 445, MLS 447, MLS 449.

**Loricariidae**

***Hypoptopoma psilogaster*** Fowler, 1915

Restricted to Peru (Aquino and Schaefer 2010).

***Farlowella platorynchus*** Retzer & Page, 1997

Junior synonym of *Farlowella amazona* (Günther, 1864) (Covain et al. 2016).

***Pterosturisoma microps*** (Eigenmann & Allen, 1942)

There are no verified records in Colombian collections. The source for its inclusion (Ortega et al. 2006), only cited specimens from MUSM.

***Dasyloricaria capetensis*** (Meek & Hildebrand, 1913)

Junior synonym of *Dasyloricaria latiura* (Eigenmann & Vance, 1912) (Londoño-Burbano and Reis 2016).

***Dasyloricaria seminuda*** Eigenmann & Vance, 1912

Junior synonym of *Dasyloricaria filamentosa* (Steindachner, 1878) (Londoño-Burbano and Reis 2016).

***Loricariichthys maculatus*** (Bloch, 1794)

Described from Surinam (Ferraris 2003). The lot IAvH-P 3253 supporting its inclusion in Maldonado-Ocampo et al. (2008) corresponds to *Loricariichthys brunneus* (Hancock, 1828).

***Planiloricaria cryptodon*** (Isbrücker, 1971)

There are no verified records in Colombian collections. The source for its inclusion (Ortega et al. 2006), only cited specimens from MUSM.

***Rineloricaria nigricauda*** (Regan, 1904)

Known only from the Sipaliwini River, Surinam (Ferraris 2007). Mojica et al. (2005) did not include this species for the Colombian Amazon as indicated in Maldonado-Ocampo et al. (2008). Bogotá-Gregory and Maldonado-Ocampo (2006a) incorporated this species based on FMNH holdings (FMNH 105722 collected in the Amazon River near Leticia), but we presumed that this lot must be misidentified.

***Chaetostoma alternifasciatum*** Fowler, 1945

Junior synonym of *Chaetostoma vagum* Fowler, 1943 (Lujan et al. 2015b).

***Chaetostoma dupouii*** Fernández-Yépez, 1945

Restricted to the Tuy River in northern Venezuela (Fisch-Muller 2003). The lot IAvH-P 3369 that supports this record for the Colombian Orinoco Basin actually corresponds to two species mixed in the same lot: *Chaetostoma dorsale* Eigenmann, 1922 and an undescribed species informally named in Colombian collections as *Chaetostoma* “ojo grande” (large eye) (Alexander Urbano-Bonilla pers. com.). Ballen et al. (2016a) also concluded that this species is not distributed in the Orinoco River basin in Colombia, based on the examination of specimens in Colombian collections identified as this species, corresponding instead to misidentifications of *C. dorsale*.

***Chaetostoma nudirostre*** Lütken, 1874

Known only from the Lago de Valencia Basin, Venezuela (Fisch-Muller 2003). The lot IAvH-P 1984 that supports the inclusion of the species in the Colombian Orinoco corresponds to an undescribed species.

***Chaetostoma sovichthys*** Schultz, 1944

Known only from the Lago de Maracaibo Basin in Venezuela (Fisch-Muller 2003). Three different species are found in the Catatumbo River drainage in Colombia, one corresponding to an undescribed species (Armando Ortega-Lara pers. obs.).

***Dolichancistrus pediculatus*** (Eigenmann, 1918)

Junior synonym of *Dolichancistrus fuesslii* (Steindachner, 1911) (Ballen and Vari 2012).

***Peckoltia braueri*** (Eigenmann, 1912)

Distributed in the Takutu, Pirara, and Branco Rivers and Uraricoera River drainage in Brazil (Armbruster 2008). Contrary to what is implicitly indicated by Maldonado-Ocampo et al. (2008), Armbruster (2008) did not list records from Colombia.

***Pseudacanthicus spinosus*** (Castelnau, 1855)

Originally included in an unpublished dissertation (Calderón and Hincapié 2001) and then cited in Bogotá-Gregory and Maldonado-Ocampo (2006a), but no confirmed records are provided for Colombian collections.

***Hypostomus watwata*** Hancock, 1828

Distributed in coastal drainages of northern South America from Oyapock River to Demerara River, and Venezuela (Ferraris 2007). Lasso et al. (2005) listed the species in the Orinoco River Basin only for the delta in Venezuela.

***Hypostomus winzi*** (Fowler, 1945)

Junior synonym of *Isorineloricaria tenuicauda* (Steindachner, 1878) (Ray and Armbruster 2016).

***Pterygoplichthys punctatus*** (Kner, 1854)

Recently redescribed (Armbruster and Page 2006) and its distribution was restricted to the Madeira River drainage and the Urubu River in Brazil. Additionally, these authors described the new species *Pterygopichthys weberi* for the Caquetá River and upper Amazon River drainages of Colombia, which may constitute the basis for the records of *P. punctatus* in Colombia. Bogotá-Gregory and Maldonado-Ocampo (2006a) cited as sources its inclusion, Santos (2000) and Vejarano (2000). However, considering that these unpublished dissertations precede the recent reappraisal of the species (Armbruster and Page 2006), it is probable that their records were based on specimens of *P. weberi*. Contrary to what is cited in Maldonado-Ocampo et al. (2008), *P. punctatus* was not listed in Mojica et al. (2005).

***Squaliforma squalina*** (Jardine, 1841)

Junior synonym of *Aphanotorulus emarginatus* (Valenciennes, 1840) (Ray and Armbruster 2016).

***Ancistrus brevifilis*** Eigenmann, 1920

Known only from the coastal basins of northern Venezuela (Caribe and Golfo de Paria) and Lago de Valencia Basin (Weber 2003, Lasso et al. 2004). The species was not listed for the Orinoco in Lasso et al. (2005) or for the Amazon in Mojica et al. (2005) as cited by Maldonado-Ocampo et al. (2008). Record of this species for the Amazon Basin in Arbeláez et al. (2004) and cited by Bogotá-Gregory and Maldonado-Ocampo (2006a) seems to correspond to a misidentification.

***Ancistrus eustictus*** (Fowler, 1945)

Junior synonym of *Ancistrus centrolepis* Regan, 1913 (Taphorn et al. 2013).

***Ancistrus gymnorhynchus*** Kner, 1854

Restricted to the coastal rivers of Falcón, Lara, Yaracuy, and Carabobo states, and Chirgua River and tributaries (upper drainage of Pao River) of the Orinoco system in Cojedes state, northern Venezuela (Taphorn et al. 2010).

***Ancistrus hoplogenys*** (Günther, 1864)

Originally listed in Lasso et al. (2005), but all collections therein cited are from Venezuela.

***Ancistrus latifrons*** (Günther, 1869)

Distributed in the upper Amazon River Basin and Solimões River in Brazil and Peru (Fisch-Muller 2003). Inclusion in Maldonado-Ocampo et al. (2008) for the Orinoco was based on Lasso et al. (2005), however, this species was not listed there.

***Lasiancistrus heteracanthus*** (Günther, 1869)

The only source for the inclusion of this species in Bogotá-Gregory and Maldonado-Ocampo (2006a) is an unpublished dissertation (Cipamocha 2002), as the other reference listed (Armbruster 2005) does not mention any specimen coming from Colombia. The lot ICN-MHN 6119 cited in Cipamocha (2002) was collected in Peru.

**Cetopsidae**

***Cetopsis oliveirai*** (Lundberg & Rapp Py-Daniel, 1994)

This record was based on a MUSM lot from the Peruvian part of the Putumayo River, Amazon Basin (Ortega et al. 2006). There are no confirmed records of this species in Colombia.

***Cetopsis plumbea*** Steindachner, 1882

Distributed in the western portions of the Amazon Basin in eastern Ecuador, southeastern Peru, and northeastern Bolivia (Vari et al. 2005). Lasso et al. (2005) listed IAvH-P and ICN-MHN supporting its presence in the Aracuca and Meta Rivers (Orinoco system). However, IAvH-P lacks of records of this species and records at ICN-MHN still identified as this species must correspond to outdated identifications of *C. orinoco* (Schultz, 1944), a species formerly described as a subspecies of *C. plumbea*.

***Denticetopsis macilenta*** (Eigenmann, 1912)

Known only from the Potaro River, Essequibo River Basin in Guyana (Vari et al. 2005). Records in Venezuelan collections (MBUCV, MCNG) cited in Lasso et al. (2005) are probably based on misidentified specimens of other species occurring in the Orinoco, since the revisionary work of Vari et al. (2005) was not available yet. In addition we failed to locate any mention in this last work recording the species for Colombia.

***Denticetopsis praecox*** (Ferraris & Brown, 1991)

Known only from the Baria River, upper Negro River drainage in Venezuela. Records identified as this species at IAvH-P from the Amazon Basin, actually corresponds to *Denticetopsis seducta* Vari, Ferraris & de Pinna, 2005. Thus we supposed that the ICN-MHN records (5193, 6216, 6276, 7753, 7754, 10447, 10675) listed in Mojica et al. (2005) are misidentified specimens of *D. seducta*, as evidenced from the pictured specimen (pl. 80a, p. 505) in Galvis et al. (2007b), which corresponds to one of the above referred lots. These lots also form the basis for citations in Prieto (2000) and Arbeláez et al. (2004), included in Bogotá-Gregory and Maldonado-Ocampo (2006a).

**Aspredinidae**

***Bunocephalus amaurus*** Eigenmann, 1912

Recorded only for the coastal rivers of northern South America, between the mouths of the Orinoco and Amazon rivers (Friel 2003). The records from the Orinoco River Basin are provisionally assigned to *Bunocephalus aloikae* Hoedeman, 1961, following Cardoso (2008).

***Bunocephalus chamaizelus*** Eigenmann, 1912

Known only from the Essequibo River Basin (Friel 2003). Originally listed in Mojica (1999), its presence in the Colombian Amazon (Bogotá-Gregory and Maldonado-Ocampo 2006a) is probably based on a misidentification.

**Auchenipteridae**

***Centromochlus megalops*** Kner, 1858

Junior synonym of *Centromochlus heckelii* (De Filippi, 1853) (Mees 1974). *Species inquirenda* in *Centromochlus* (Ferraris 2007).

***Auchenipterichthys thoracatus*** (Kner, 1857)

Restricted to the upper basin of the Madeira River in Bolivia and Brazil (Ferraris et al. 2005). The samples coming from the Amazon River Basin actually correspond to *Auchenipterichthys coracoideus* Eigenmann & Allen, 1942, which was not listed in Maldonado-Ocampo et al. (2008).

***Auchenipterus brachyurus*** (Cope, 1878)

Not included in Galvis et al. (2007b), listed as the only supporting reference in Maldonado-Ocampo et al. (2008).

***Tetranematichthys quadrifilis*** (Kner, 1857)

Restricted to the Guaporé River Basin, upper basin of Madeira River (Vari and Ferraris 2006).

**Doradidae**

***Doras carinatus*** (Linnaeus, 1766)

Recorded only for rivers draining the north side of the Guiana shield: Amapá state of Brazil, French Guiana, Guyana, and Suriname (Sabaj Pérez and Birindelli 2008). Contrary to Maldonado-Ocampo et al. (2008), this species was not listed in Mojica et al. (2005) neither in Ortega et al. (2006). Records in Santos (2000) and Vejarano (2000) listed in Bogotá-Gregory and Maldonado-Ocampo et al. (2005, 2006a) are probably based on misidentified specimens of *Doras phlyzakion* Sabaj Pérez & Birindelli, 2008, name not yet available for the cited authors in 2000.

***Hemidoras morei*** (Steindachner, 1881)

The lot IAvH-P 2112 that forms the basis for its inclusion in Bogotá-Gregory and Maldonado-Ocampo (2006a) actually corresponds to *Ossancora punctata* (Kner, 1855) (identified by Mark H. Sabaj).

***Leptodoras copei*** (Fernández-Yépez, 1968)

Lasso et al. (2005) listed this species for Venezuelan tributaries of the Orinoco River, while Bogotá-Gregory and Maldonado-Ocampo (2006a) supported its presence in the Amazon Basin in Sabaj (2005); however, Colombian records are not provided in this last reference.

***Platydoras costatus*** (Linnaeus, 1758)

Restricted to the coastal river basins of Surinam and French Guiana (i.e. Corantijn, Surinam, Maroni) (Piorski et al. 2008). *Platydoras armatulus* (Valenciennes, 1840) (Amazon and Orinoco River basins) and *P. hancockii* (Valenciennes, 1840) (Orinoco River Basin) are the only species found in Colombia (Piorski et al. 2008; pers. obs.).

**Heptapteridae**

***Cetopsorhamdia phantasia*** Stewart, 1985

This record is based on a MUSM lot from the Peruvian part of the Putumayo River, Amazon system (Ortega et al. 2006). Confirmed records are lacking in Colombian collections.

***Heptapterus mustelinus*** (Valenciennes, 1835)

Restricted to La Plata and Uruguay River basins and coastal basins of southern Brazil (Bockmann and Guazzelli 2003).

***Leptorhamdia marmorata*** Myers, 1928

Lasso et al. (2005) listed only Venezuelan collections, thus its presence in Colombia is not supported by verified lots.

***Pimelodella altipinnis*** (Steindachner, 1864)

Restricted to the Essequibo River Basin (Bockmann and Guazzelli 2003). The lot ICN-MHN 7217, which is the base for its inclusion for the Colombian Amazon in Mojica et al. (2005), and later illustrated in Galvis et al. (2006, pl. 71c, p. 496) actually corresponds to an undescribed pimelodid catfish species of the genus *Propimelodus* (Lundberg et al. in prep.).

***Pimelodella hasemani*** Eigenmann, 1917

The bibliographic reference listed in Maldonado-Ocampo et al. (2008) as supporting its presence in the Colombian Amazon (Ortega et al. 2006) does not provide records from the Colombian collections.

***Rhamdella* sp.**

Not listed in Galvis et al. (2007b), the single supporting reference for its inclusion in Maldonado-Ocampo et al. (2008). Likewise, the species belonging to *Rhamdella* are restricted to the Beni River in Bolivia, Itiyuro River drainage in northern Argentina, Uruguay and Jacuí River basins in southern Brazil and coastal rivers of southern Santa Catarina state and northeastern Rio Grande do Sul state, Brazil (Bockmann and Miquelarena 2008, Reis et al. 2014).

***Rhamdia humilis*** (Günther, 1864)

Restricted to the Tuy River Basin in the north central coast of Venezuela (Silfvergrip 1996, Carlos DoNascimiento pers. obs.).

**Pimelodidae**

***Brachyplatystoma capapretum*** Lundberg & Akama, 2005

There are no records in collections that confirm its presence in the Colombian Amazon. Bogotá-Gregory and Maldonado-Ocampo (2006a) do not provide lots or bibliographic references to support its presence in Colombia.

***Bergiaria westermanni*** (Lütken, 1874)

Known only from Das Velhas River in the São Francisco River Basin, Brazil (Lundberg and Littmann 2003). Bogotá-Gregory and Maldonado-Ocampo (2006a) cited an IAvH-P lot supporting its presence in the Colombian Amazon; however, an exhaustive search of all pimelodid records in this collection was unsuccessful in locating this species.

***Cheirocerus eques*** Eigenmann, 1917

Ortega et al. (2006) listed only MUSM, thus its presence in Colombia is not supported by verified lots.

***Pimelodus maculatus*** La Cepède, 1803

Restricted to the Parana and São Francisco River basins (Lundberg and Littmann 2003, Carlos DoNascimiento pers. obs.).

**GOBIIFORMES**

**Eleotridae**

***Microphilypnus amazonicus*** Myers, 1927

Junior synonym of *Microphilypnus ternetzi* Myers, 1927 (Caires and Figueiredo 2011).

**PLEURONECTIFORMES**

**Achiridae**

***Achirus achirus*** (Linnaeus, 1758)

Predominantly marine, highland Amazonian records (e.g. Peru) are based on misidentifications of *Hypoclinemus mentalis* (Günther, 1862) (Ramos 2003a). Effectively, lots from the Amazon that were identified as *Achirus achirus*, actually correspond to *Hypoclinemus mentalis* (Günther 1862) (IAvH-P 2051, 4843) or even *Apionichthys nattereri* (Steindachner, 1876) (IAvH-P 3994). On the other hand a lot coming from the Atrato River (IAvH-P 11144) proved to be a misidentification of *Trinectes paulistanus* (Miranda Ribeiro, 1915). Thus, records of *A. achirus* in Castro and Arboleda (1988), Castro (1994), Mojica (1999), and Calderón and Hincapié (2001) are probably based on diverse misidentified achirids (mainly *Hypoclinemus mentalis*).

***Apionichthys dumerili*** Kaup, 1858

Distributed in estuarine enviroments of the Orinoco, Corantjin, Oiapoque, Amazon, and Grajaú (Ramos 2003b). The record of Castro and Arboleda (1988) is probably based on a misidentification.

**CICHLIFORMES**

**Cichlidae**

***Chaetobranchus semifasciatus*** Steindachner, 1875

The single lot (IAvH-P 5662) supporting this record in Maldonado-Ocampo et al. (2008) actually corresponds to *Chaetobranchus flavescens* Heckel, 1840.

***Crenicichla proteus*** Cope, 1872

No verified records in Colombian collections. The source for its inclusion (Ortega et al. 2006), only cited specimens from MUSM.

***Crenicichla saxatilis*** (Linnaeus, 1758)

Distributed in Barima and Corantijn Rivers (Guyana), Suriname and Marowijne Rivers (Surinam), Mana, Cayenne, Approuague and Oyapock Rivers (French Guiana), and Rio Araguari (Brazil) (Ploeg, 1991), as well as in Atlantic coastal drainages of Venezuela and Trinidad Island (Kullander, 2003).

***Crenicichla vittata*** Heckel, 1840

Distributed in the Parana, Paraguay, and Uruguay Rivers (Kullander 2003). Originally reported in Calderón and Hincapié (2001) for the Amazon Basin and listed in Bogotá-Gregory and Maldonado-Ocampo (2006a). This record is probably based on a misidentification.

***Crenicichla wallacii*** Regan, 1905

Restricted to the Essequibo River (Guyana) and the Rio Branco (Brazil) (Ploeg, 1991).

***Dicrossus maculatus*** Steindachner, 1875

The specimens listed in Galvis et al. (2007a) as this species correspond to *Dicrossus gladicauda* Schindler and Staeck, 2008, distinguished from *D. maculatus* by the squared or rectangular shape of the dark blotches on the sides of the body (Schindler and Staeck 2008), being also described in this way in Galvis et al. (2007a), and also apparent from the pictured specimen in figure 346 (p. 397) of this same work.

***Apistogramma commbrae*** (Regan, 1906)

Distributed in the Paraguay River Basin and middle basin of the Parana River in Argentina and Uruguay (Kullander 2003). Not listed in Mojica et al. (2005), thus the only source that supports its inclusion in the Colombian Amazon is Calderón and Hincapié (2001), cited in Bogotá-Gregory and Maldonado-Ocampo (2006a), and is probably based on a misidentification.

***Apistogramma geisleri*** Meinken, 1971

Bogotá-Gregory and Maldonado-Ocampo (2006a) supported its inclusion for the Colombian Amazon, based on a reference to an unpublished dissertation (Santos 2000) and one lot (ICN-MHN 5229). However identity of this record has not been confirmed, and since the species is only known from the lower Trombetas River drainage in Brazil (Kullander 2003), we opt to remove this species until the identification of the above cited lot is confirmed.

***Apistogramma inconspicua*** Kullander, 1983

The only source that supports its inclusion in the Colombian Amazon is the unpublished dissertation of Calderón and Hincapié (2001), cited in Bogotá-Gregory and Maldonado-Ocampo (2006a), which is not followed here as a reliable reference.

***Apistogramma ortmanni*** (Eigenmann, 1912)

Distributed in the Essequibo River Basin, including the Cuyuní, Potaro, and Rupununi, and in the Corantijn River Basin (Kullander 2003). In Venezuela is restricted to the Cuyuní River drainage (Lasso et al. 2004). The single record cited in Arbeláez et al. (2004): ICN-MHN 4936, was identified as *Apistogramma* cf. *ortmanni*, while the species was not listed in Mojica et al. (2005), as implied in Maldonado-Ocampo et al. (2008).

***Apistogramma pertensis*** (Haseman, 1911)

The lot IAvH-P 2346 (Bogotá-Gregory and Maldonado-Ocampo 2005) that is the basis for inclusion of this species in the Colombian Amazon, actually corresponds to *Apistogramma bitaeniata* (Sven O. Kullander pers. com.).

***Apistogramma regani*** Kullander, 1980

Not known from Colombia (Sven O. Kullander pers. com.)

***Apistogramma uaupesi*** Kullander, 1980

Contrary to what is indicated in Maldonado-Ocampo et al. (2008), no mention of the species is found in Maldonado-Ocampo (2004), Maldonado-Ocampo et al. (2006a) or Galvis et al. (2007b). In addition, the presence of the species in the Orinoco River Basin (Lasso et al. 2005) is based only in Venezuelan records (MBUCV, MCNG).

***Geophagus brasiliensis*** (Quoy & Gaimard, 1824)

Distributed in the coastal basins of east and south Brazil and Uruguay (Kullander 2003). A single record (ICN-MHN 4346) supports its inclusion for the Amazon Basin in Correa (2003) as *Geophagus* cf. *brasiliensis*, and then cited in Bogotá-Gregory and Maldonado-Ocampo (2006a). Given the uncertainty on the actual taxonomic identity of the above mentioned record and the disjunt geographic distribution of the species we prefer to exclude the species from Colombia.

***Mikrogeophagus altispinosus*** (Haseman, 1911)

Not included in Galvis et al. (2007b) as indicated in Maldonado-Ocampo et al. (2008), and lacking of confirmed records in Colombian collections.

***Satanoperca acuticeps*** (Heckel, 1840)

There are no records in the Colombian collections accessed. The record in Castro and Arboleda (1988) is probably based on a misidentification.

***Satanoperca leucosticta*** (Müller & Troschel, 1849)

The lots listed in Lasso et al. (2005) as supporting its presence in the Colombian Orinoco, actually corresponds to *Satanoperca mapiritensis* (IAvH-P 1079, 1563) and *S. daemon* (IAvH-P 2813). Species not known from Colombia (Sven O. Kullander pers. com.).

***Satanoperca pappaterra*** (Heckel, 1840)

Known only from the Amazon Basin in the Guaporé River in Bolivia and Brazil, and is also distributed in the Paraguay River Basin in Brazil and north of Paraguay (Kullander 2003). Originally reported in Calderón and Hincapié (2001) for the Colombian Amazon, although this record is probably based on a misidentification.

***Aequidens pallidus*** (Heckel, 1840)

Only included in an unpublished dissertation (Calderón and Hincapié 2001), given no mention of the species is provided in Mojica et al. (2005), being both works cited in Bogotá-Gregory and Maldonado-Ocampo (2006a). No confirmed records are provided for Colombian collections.

***Aequidens potaroensis*** Eigenmann, 1912

Recorded only from the Essequibo River Basin in Guyana (Kullander 2003). The records from the Putumayo River in IAvH-P correspond to *Aequidens diadema* (Heckel, 1840) (IAvH-P 1739) and *A. tetramerus* (Heckel, 1840) (IAvH-P 286, 1737, 1765, 1825, 2350, 3159). The following lots (ICN-MHN 1507, ICN-MHN 5040, ICN-MHN 6146, ICN-MHN 6250, ICN-MHN 6712) identified as this species in Mojica *et al*. (2005) must probably correspond to misidentified specimens.

***Andinoacara*** ***coeruleopunctatus*** (Kner & Steindachner, 1863)

The species has not records in Colombian collections (Maldonado-Ocampo et al. 2013b).

***Andinoacara pulcher*** (Gill, 1858)

Naturally distributed in Trinidad and Tobago and Venezuela (Kullander 2003). In Venezuela is found in the rivers of northern Venezuela from the Caribe and Golfo de Paria basins and in the Lago de Valencia Basin (Lasso et al. 2004). The species has been introduced elsewhere.

***Bujurquina huallagae*** Kullander, 1986

No verified records in Colombian collections. The source for its inclusion (Ortega et al. 2006), only cited specimens from MUSM.

***Bujurquina ortegai*** Kullander, 1986

No verified records in Colombian collections. The source for its inclusion (Ortega et al. 2006), only referred specimens from MUSM.

***Bujurquina vittata*** (Heckel, 1840)

Distributed in the Parana and Paraguay River basins (Kullander 2003). Originally reported for the Amazon Basin in the following references accounted in Bogotá-Gregory and Maldonado-Ocampo (2006a): Castro and Arboleda (1988), Mojica (1999), and Calderón and Hincapié (2001). However, these records are probably based on misidentifications, since the species has not been included in more recent revisions on the Amazonian ichthyofauna (Mojica et al. 2005, Galvis et al. 2006, Galvis et al. 2007b).

***Cichlasoma bimaculatum*** (Linnaeus, 1758)

Distributed in the Cottica, Suriname, Nickerie, Corantijn, Lucie, upper Cuyuní, Moruka, and Branco rivers in Surinam, French Guiana, Guyana, Venezuela, and Brazil (Kullander 1983).

***Cichlasoma dimerus*** (Heckel, 1840)

Distributed in the Parana and Paraguay River basins (Kullander 2003). Originally reported in Calderón and Hincapié (2001) for the Amazon Basin and listed in Bogotá-Gregory and Maldonado-Ocampo (2006a). This record is probably based on a misidentification.

***Cichlasoma taenia*** (Bennett, 1831)

Distributed in Trinidad and Tobago and northeastern Venezuela (Kullander 2003). Listed for the Tomo River in Lasso et al. (2005), but there are no confirmed records in Colombian collections.

***Mesonauta festivus*** (Heckel, 1840)

Distributed in the Bolivian Amazon, including the Madre de Dios in Peru, and the Guapore-Mamore drainage, the Paraguay drainage, Rio Jamari and lower Rio Tapajos (Kullander and Silfvergrip 1991).

***Pterophyllum leopoldi*** (Gosse, 1963)

The single lot (IAvH-P 504) supporting this record in Bogotá-Gregory and Maldonado-Ocampo (2005) actually corresponds to *Pterophyllum scalare* (Schultze, 1823).

**BELONIFORMES**

**Belonidae**

***Belonion apodion*** Collette, 1966

Known only from the Guaporé and Madeira River basins in Bolivia and Brazil (Lovejoy and Collette 2003). There are no confirmed records in Colombian collections. Besides the species is not listed in Maldonado-Ocampo et al. (2006a) as indicated in Maldonado-Ocampo et al. (2008).

**CYPRINODONTIFORMES**

**Cynolebiidae**

***Anablepsoides limoncochae*** (Hoedeman, 1962)

Known only from the Ecuadorian Amazon (Costa 2003). Record in Galvis et al (2007a) is probably based on a misidentification, given this species has not been recorded in the Venezuelan Orinoco River Basin (Lasso et al. 2005).

***Anablepsoides urophthalmus*** (Günther, 1866)

This species has not verified records in Colombian collections. The source for the inclusion of this species (Ortega et al. 2006), only cited specimens from MUSM.

**Poeciliidae**

***Poecilia cuneata*** Garman, 1895

Junior synonym of *Poecilia gillii* (Kner, 1863) (Poeser 2003a).
